# Clinical protocol: Feasibility of evaluating abemaciclib neuropharmacokinetics of diffuse midline glioma using intratumoral microdialysis

**DOI:** 10.1371/journal.pone.0291068

**Published:** 2023-09-08

**Authors:** Edjah K. Nduom, John Glod, Desmond A. Brown, Margaret Fagan, Mahalia Dalmage, John Heiss, Seth M. Steinberg, Cody Peer, William D. Figg, Sadhana Jackson

**Affiliations:** 1 Department of Neurosurgery, Emory University School of Medicine, Atlanta, GA, United States of America; 2 Pediatric Oncology Branch, National Cancer Institute, National Institutes of Health, Bethesda, MD, United States of America; 3 Surgical Neurology Branch, National Institute of Neurological Disorders and Stroke, National Institutes of Health, Bethesda, MD, United States of America; 4 Biostatistics and Data Management Section, Office of the Clinical Director, Center for Cancer Research, National Cancer Institute, National Institutes of Health, Bethesda, MD, United States of America; 5 Clinical Pharmacology, National Cancer Institute, National Institutes of Health, Bethesda, MD, United States of America; Goethe University Hospital Frankfurt, GERMANY

## Abstract

Diffuse midline gliomas (DMG) are the most aggressive brain tumors of childhood and young adults, with documented 2-year survival rates <10%. Treatment failure is due in part to the function of the BBB. Intratumoral microdialysis sampling is an effective tool to determine brain entry of varied agents and could help to provide a better understanding of the relationship of drug permeability to DMG treatment responsivity. This is a non-randomized, single-center, phase 1 clinical trial. Up to seven young adult (18–39 years) patients with recurrent high-grade or diffuse midline glioma will be enrolled with the goal of 5 patients completing the trial over an anticipated 24 months. All patients will take abemaciclib pre-operatively for 4.5 days at twice daily dosing. Patients will undergo resection or biopsy, placement of a microdialysis catheter, and 48 hours of dialysate sampling coupled with timed plasma collections. If intratumoral tumor or brain dialysate sampling concentrations are >10nmol/L, or tumor tissue studies demonstrate CDK inhibition, then restart of abemaciclib therapy along with temozolomide will be administered for maintenance therapy and discontinued with evidence of radiologic or clinical disease progression. The poor survival associated with diffuse midline gliomas underscore the need for improved means to evaluate efficacy of drug delivery to tumor and peritumoral tissue. The findings of this novel study, will provide real-time measurements of BBB function which have the potential to influence future prognostic and diagnostic decisions in such a lethal disease with limited treatment options.

**Trial registration:** Clinicaltrials.gov, NCT05413304. Registered June 10, 2022, Abemaciclib Neuropharmacokinetics of Diffuse Midline Glioma Using Intratumoral Microdialysis.

## Introduction

Diffuse midline gliomas (DMGs) compose a rare subset of adult diffuse gliomas that often harbor histone H3 mutations (H3 K27M mutations). Recent studies have demonstrated median overall survival to be 19.6 months and 25 months in patients who received radiotherapy alone or with temozolomide (TMZ). Adults harboring H3 K27M-mutant gliomas predominate in patients aged < 40 years, and while these gliomas are rare in the entire population of adult IDH wild-type diffuse gliomas, they are frequent among the subset of tumors found within the midline [1–4]. DMGs tumors are often pathologically distinguished as infiltrative high-grade (malignant) gliomas. Treatment failure is due in part to the presence of the blood-brain barrier (BBB), which limits permeability of many otherwise well-selected chemotherapeutic agents [5–8]. Midline gliomas grow diffusely and infiltrate critical midline structures including but not limited to the brainstem and thalamus; often rendering complete surgical resection unsafe. Despite several investigational trials for adults with midline gliomas, only radiation therapy (RT) has ever demonstrated any anti-tumor effect or significant improvement in outcome and the beneficial anti-tumor effects of RT are short-lived. Over the past three decades, many clinical trials have explored the use of various chemotherapeutic agents for pontine based DMGs, employing conventional cytotoxic agents, high-dose chemotherapy strategies, chemo-radiotherapy, and molecularly targeted agents. However, no chemotherapeutic agent has *ever* demonstrated significant efficacy against pontine DMGs with or without H3K27M mutations [9, 10].

One of the main attributes of disease progression/proliferation in DMGs is associated with dysregulation of the cell cycle via cyclins and cyclin dependent kinases [11]. Dysregulation of this ordered progression is a common feature of many cancers and frequent somatic alteration of cyclin D, CDKN2A and CDK4 are common features in multiple tumor types including breast, head and neck tumors, non-small cell lung cancer, melanoma and glioblastoma. Abemaciclib (Verzenio®) is a CDK4/6 inhibitor that is FDA approved for the treatment of metastatic breast cancer [12]. Abemaciclib has a distinct toxicity profile, likely due to its increased specificity for CDK4 [13]. Previous studies evaluating overall response rates post treatment in brain stem gliomas have demonstrated some efficacy in inhibition of the CDK pathway. Studies using glioblastoma xenograft models demonstrated the ability of abemaciclib to cross the blood-brain barrier, increase survival, and decrease tumor growth when given as a single agent or in combination with temozolomide. Recent studies demonstrated disease stabilization in 3/17 glioblastoma patients for up to 23 cycles [14].

While it has shown efficacy in a preclinical brainstem glioma model alone and with combination therapy, details of brainstem drug entry pharmacokinetics and pharmacodynamics are still needed [13, 15, 16]. Evaluations of abemaciclib brain permeability have been limited to cerebrospinal fluid (CSF) sampling in metastatic brain tumor patients [14]. This study found CSF concentrations approached plasma concentrations with a CSF concentration range of 2.2–14.7 nmol/L. There is currently one open-enrolling Phase I clinical trial using abemaciclib with escalating dosing in children with DIPG or recurrent/refractory solid tumors (NCT02644460). The NCT02644460 study is being performed to assess the maximum tolerated dosing of abemaciclib in combination with radiation therapy and then for continuous treatment thereafter for a maximum duration of two years. There are currently five open-enrolling clinical trials using abemaciclib alone or in combination with other agents (NCT02644460, NCT03220646, NCT04391595, NCT04074785, NCT02981940) in patients with malignant brain tumors. None of these studies utilize microdialysis combined with genomic profiling to determine further treatment allowances. Alexander et al., through the INSIGhT collaborative study designed an adult newly diagnosed grade IV glioma precision medicine trial involving Abemaciclib (150-200mg po BID) post radiation/temozolomide treatment and demonstrated favorable PFS (p = 0.03, log-rank test) with abemaciclib (median 6.31 months; 95% CI: 5.29–8.18) compared to the control arm (5.16 months; 95% CI: 4.37–6.28). Abemaciclib was generally well-tolerated with any new drug-related toxicities [17].

Efforts to evaluate drug delivery across the BBB in midline gliomas have been restricted to post biopsy specimens. In comparison, intracerebral microdialysis sampling of cortical tissue has been shown to be a highly effective tool in determining cortical neuropharmacokinetics (brain extracellular fluid penetration, accumulation and excretion) intratumorally and peritumorally in adult brain tumor patients [18–26]. Intratumorally implanted microdialysis catheter sampling is a means to measure varied drugs and solutes within brain interstitial fluid versus plasma concentrations; with limitations in evaluating intracellular concentrations. A clear advantage to surgical resection/biopsy tissue exploration is that dialysate sampling can provide measurements of drugs, solutes, cytokine, etc. dynamic shifts over time (up to 2 weeks); allowing the clinical team to use this data to make diagnostic and prognostic decisions [23, 27–30]. To date, this technology has been used widely for traumatic brain injury, and neurologic disorders to monitor metabolites, proteins and CNS drug levels [21, 22, 24–26, 31–34]. This surgical procedure has been successfully and safely performed at the NIH in adult high grade glioma patients with cortical lesions [20, 29]. However, it is underutilized in adults and has *never* been utilized in the midline glioma setting to evaluate chemotherapy or targeted therapy permeability.

The tumor biology and drug distribution pattern within the midline region is largely unknown [35, 36]. One of the main challenges of effectively treating DMG includes the ability to reach therapeutic drug exposure at the target site. Drug concentrations in the midline region are often extrapolated from cortical measurements, due to ease of sampling, in an effort to correlate proposed drug entry to treatment responsivity. However, previous studies demonstrate that brain pharmacology properties including drug half-life, hydrophobicity, metabolism, active compound distribution, multi-drug resistance protein substrate qualities, charge and molecular weight collectively make it difficult to understand tissue concentration at one specified time point [37]. To date, all phase 0 DMG studies specifically evaluating brainstem drug concentrations have evaluated biopsied tissue samples, which evaluates only drug entry at the surgical time based on drug protein binding, distribution patterns and characteristics of permeability. However, microdialysis sampling is an exquisite tool that can account for the same pharmacologic attributes while also allowing for extrapolation of intratumoral shifts over time based on drug metabolism and post surgical healing. Our study will combine data of drug pharmacokinetics from biopsied or resected tissue along with extracellular fluid concentrations to garner a more detailed display of drug concentrations over time, clearance and distribution.

This proposal will specifically measure biopsied tissue and brain interstitial fluid to compare both intracellular and extracellular abemaciclib concentrations. Microdialysis studies will afford the clinical team with information regarding BBB function as it relates to abemaciclib CNS entry and clearance. Additional information afforded from the biopsied tumor tissue will be genomic profiling (whole exome sequencing) to determine molecular targets for treatment and pharmacodynamic evaluations to assess response to abemaciclib treatment. These studies aim to better understand the complex relationship between DMG tumor biology and CNS drug distribution, to more intelligently select therapies for future clinical studies.

## Materials and methods

### Study design (Figs 1 and 2)

**Fig 1:**
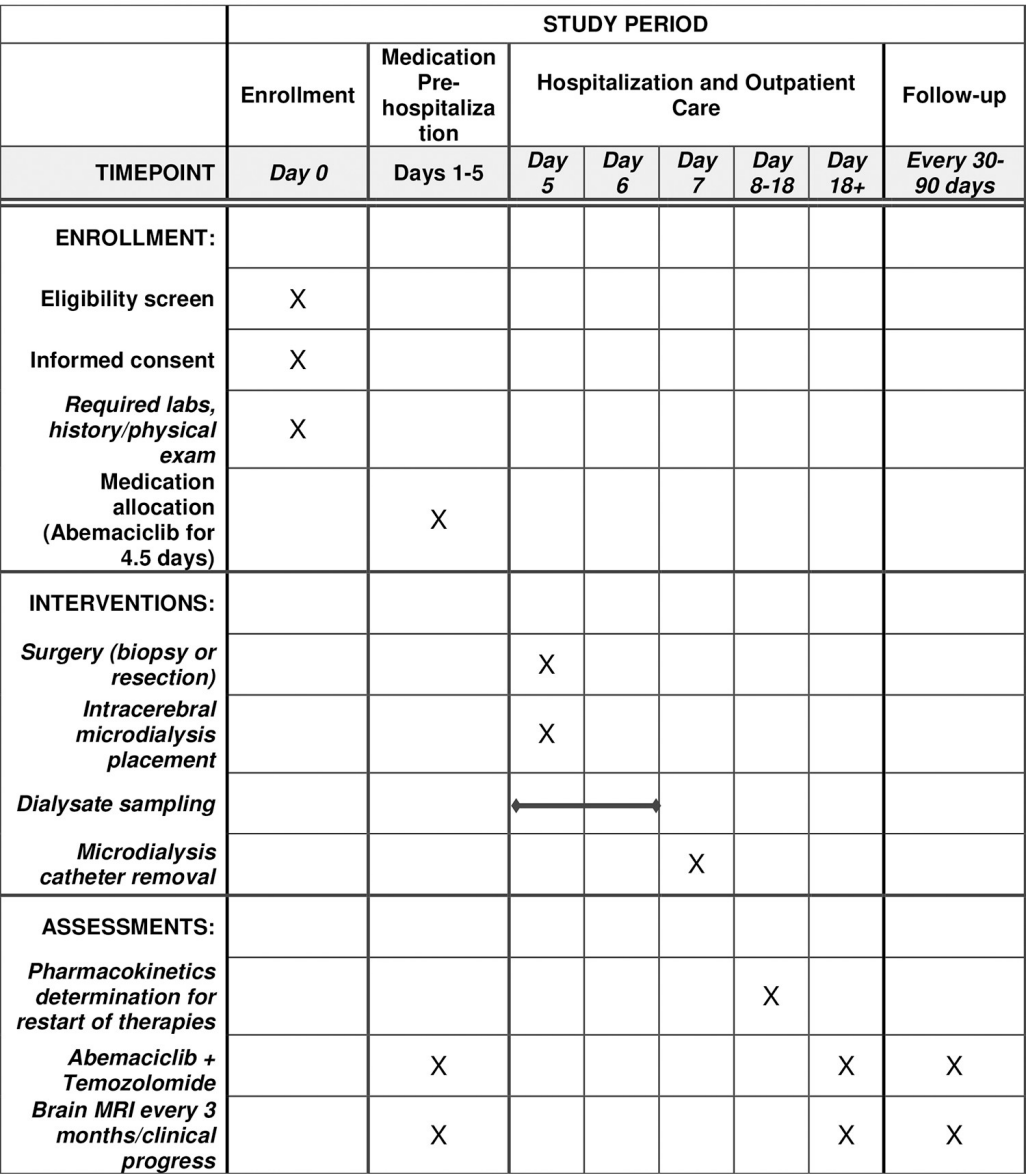
Schedule of enrollments, interventions and assessments.

**Fig 2:**
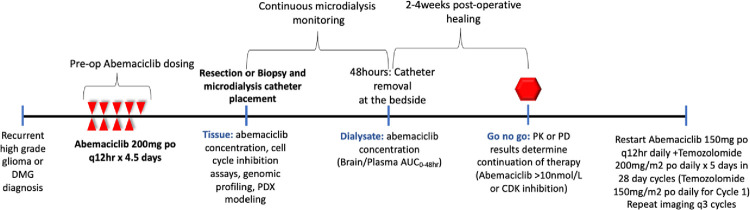
Design schema for clinical trial.

This protocol is an open-label single-armed study of the investigational off-label use of abemaciclib in recurrent high-grade and diffuse midline glioma and on-label use of temozolomide in recurrent high-grade and diffuse midline glioma for maintenance therapy. This is a safety and feasibility study to evaluate tumor pharmacokinetics (PK) and pharmacodynamics (PD) of abemaciclib in recurrent high-grade glioma and DMG participants in need of surgical resection or biopsy respectively. As such, two cohorts will be evaluated, 2 participants with cortical high-grade glioma and 3 participants with DMG.

### Study population (Table 1)

- Participants must be ≥ 18 and ≤ 39 years old at the time of enrollment
- Participants must have recurrent high-grade glioma or midline glioma based on clinical and/or radiologic findings
- Patients will be recruited to the NIH Clinical Center in Bethesda, MD.
- Target of 5 completed patients.
- Withdrawals which do not complete the initial study procedures will be replaced up to the accrual ceiling of 7 patients

**Table 1:** Inclusion/exclusion criteria for trial participation.

Inclusion Criteria	Exclusion Criteria
Participants must have recurrent high-grade glioma or midline glioma based on clinical and/or radiologic findings	Participants who cannot safely undergo a biopsy due to contraindications
Participants with cortical high-grade gliomas must have previous intra-operative pathology confirming disease	Pregnant women, or women who intend to become pregnant during the study, are excluded from this study because of the teratogenic effects of abemaciclib. Because there is an unknown but potential risk for adverse events in nursing infants secondary to treatment of the mother with these agents, breastfeeding should be discontinued if the mother is treated on study.
Participants must be ≥ 18 and ≤ 39 years old at the time of enrollment	Serious preexisting medical condition(s) that would preclude participation in this study
Ability to swallow tablets/pills	Uncontrolled intercurrent illness
Karnofsky Performance Scale ≥50% within 14 days prior to enrollment	Personal history of any of the following conditions: syncope of cardiovascular etiology, ventricular arrhythmia of pathological origin (including, but not limited to, ventricular tachycardia and ventricular fibrillation), or sudden cardiac arrest.
At least 4 weeks must have elapsed since any major surgeries, with no evidence of infections. Minimally invasive biopsies (outside of the brainstem) and central line placements are not considered major surgeries	Active systemic bacterial infection (requiring intravenous [IV] antibiotics at time of initiating study treatment), fungal infection, or detectable viral infection
Abemaciclib administration must be able to begin no later than 14 days after the date of radiographic diagnosis (by T2 or FLAIR imaging)	Requires treatment with strong/moderate CYP3A inhibitors or inducers. Participants receiving any medications or substances that are inducers or strong/moderate inhibitors of CYP3A4 are ineligible.
Participants who received chemotherapy must have recovered (Common Terminology Criteria for Adverse Events [CTCAE] Grade ≤1) from the acute effects of chemotherapy except for residual alopecia or Grade 2 peripheral neuropathy prior to randomization. A washout period of at least 21 days is required between last chemotherapy dose and randomization (provided the participant did not receive radiotherapy).	Inability to undergo MRI and/or contraindication for MRI examinations following the MRI protocol (see Study Procedure Manual). Prosthesis or orthopedic or dental braces that would interfere with MRI.
Participants who received radiotherapy must have completed and fully recovered from the acute effects of radiotherapy. A washout period of at least 14 days is required between end of radiotherapy and randomization.	Refractory nausea and vomiting that would limit drug administration
Adequate organ function within 14 days prior to enrollment	Known severe hypersensitivity to abemaciclib, temozolomide or any excipient of abemaciclib or temozolomide or history of allergic reactions attributed to compounds of similar chemical or biologic composition to abemaciclib and temozolomide.
	Participants who have received prior treatment with abemaciclib or another specific CDK4/6 inhibitor (E.g., Ribociclib, Palbociclib–list is not all inclusive)

This is an approved protocol at the National Cancer Institute, National Institutes of Health through the Scientific Review Board and Institutional Review Board (accepted 4/15/2022). Subsequently approved by ethics committees and NCI CTEP within NIH. Formal consent includes oral and written provided by the patient and/or their legal guardian by proxy, when appropriate.

### Treatment of subjects

#### Initial therapy

All participants will take abemaciclib pre-operatively for 4.5 days (9 total doses of abemaciclib) at twice daily dosing. A maximally safe surgical resection for cortical high-grade glioma or stereotactic needle biopsy for midline glioma will be performed in the operating room. Intraoperative pathology will confirm recurrent disease in order to proceed with microdialysis placement. Microdialysis insertion (based on participant safety, surgical feasibility and pathology confirmation of disease) will be performed post-biopsy in the operating room and placement will be verified by brain CT. Continuous microdialysis sampling will be obtained over the course of the next 48 hours, with subsequent removal of the catheter at the bedside. After discharge from NIH inpatient, PK and PD findings will assist in determination of whether the participant will continue to receive abemaciclib therapy. If intratumoral or PK brain dialysate sampling concentrations are >10nmol/L, or PD findings suggest CDK inhibition (decreased expression of Rb and/or topoIIα), then abemaciclib therapy will be resumed in combination with temozolomide for maintenance therapy post resection or biopsy.

#### Maintenance therapy

Maintenance therapy will be abemaciclib 150mg po BID together with temozolomide 200mg/m^2^ po daily x 5 days in 28 day cycles (temozolomide 150mg/m2 po daily x 5 days for cycle 1) [14, 16]. Additional plasma pharmacology studies will be performed on day 5 of every other cycle to evaluate effect of combination therapy compared to abemaciclib alone and temozolomide only (historical controls). After every 3 cycles, repeat brain MRI’s will be obtained to evaluate treatment response and disease progression. If a participant starts to exhibit signs of clinical deterioration or radiographic progression, the participant will discontinue use of study treatment.

The study team will maintain active contact via phone (every six months) with patients who do not go onto maintenance therapy, or with their home clinical team as part of long-term follow-up to collect information on his/her management of disease and survival post-trial. Once patients on maintenance therapy exhibit disease progression, they will continue long-term follow-up via phone (every six months) as part of long-term follow-up for survival.

#### Dose adjustments

The pre-operative dose of abemaciclib will not be adjusted for lower dosing for drug-related toxicities on this study due to the short duration of therapy (detailed PK findings of abemaciclib based on standard dosing). Any grade 3 or higher toxicity that is deemed attributable to abemaciclib within the first 4.5 days will require drug discontinuation and removal from study. Those participants will be replaced. Any other toxicities seen prior to surgery that make surgery not feasible or unsafe will also require discontinuation of therapy, removal from study and replacement. If grade 3 or higher toxicity is noted during maintenance therapy (abemaciclib combined with temozolomide), then both therapies will be held until symptoms resolve to grade 1–2. After resolution, will restart temozolomide dosing at 150mg/m^2^ (or 100mg/m^2^ if during cycle 1) with no change in abemaciclib dosing. The participant will be taken off treatment and followed until death if they experience any two Grade 4 adverse events or one Grade 5 adverse event that can be attributed to abemaciclib.

If the toxicity is likely or potentially related to abemaciclib, the appropriate dose reductions for abemaciclib must occur. If it is likely or potentially related to temozolomide, then appropriate dose reductions for temozolomide should be made. If it is not possible to attribute the toxicity to either drug, consider reducing both. If a participant must stop one of the study drugs, then they will be taken off treatment and followed until death.

Management of drug holds, and supportive care will be at the discretion of the PI. If the participant requires discontinuation of the drug due to prolonged grade 3 or higher toxicity, they will be removed from the study protocol directed therapy and monitored closely until toxicities resolve; and then every 6 months until death.

#### Blood and dialysate sampling for abemaciclib, abemaciclib metabolites and urea

Whole blood for pharmacokinetic abemaciclib mi will be obtained pre-dose, time of last dose pre-surgery (less than 14 hours from time of surgery), and then approximately 1, 2, 4, 6, 8, 10, 24, 48 and 72 hours post catheter insertion. Whole blood for urea analysis will be obtained with PK studies at approximately 2, 6, 10, 14, 18, 22, 26, 30, 34, 38, 40, 44, and 48 hours post catheter insertion. At each sample time point a discard blood volume appropriate for the IV access device must be drawn prior to the sample. Blood (3 mL) will be drawn from a peripheral vein in the participant’s arm and collected in green top tubes containing sodium heparin anticoagulant. Tubes will be promptly mixed by gently inverting 6-times, then placed on ice, until centrifuged at 1,300 x g for 10 min at 4°C. Samples will be centrifuged for harvesting plasma within 2 hours. Upon centrifugation, the plasma will be separated from the blood cells using a pipette and transferred into an appropriately labeled polypropylene freezer vial. The samples should be processed to plasma within 30 minutes from centrifugation. Plasma will then be stored frozen at -80°C until subsequent batch analysis. Urea concentration will be evaluated on both blood and dialysate collections (1–2μL/sample minimum).

#### Drug accountability

NCI-supplied agents may be requested by eligible participating Investigators (or their authorized designee) at each participating institution. The CTEP-assigned protocol number must be used for ordering all CTEP-supplied investigational agents. The eligible participating investigators at each participating institution must be registered with CTEP, DCTD through an annual submission of FDA Form 1572 (Statement of Investigator), NCI Biosketch, Agent Shipment Form, and Financial Disclosure Form (FDF). If there are several participating investigators at one institution, CTEP-supplied investigational agents for the study should be ordered under the name of one lead participating investigator at that institution. Starter supplies will not be provided. Participants must be registered prior to agent ordering.

### Main study endpoint

- To evaluate safety and feasibility of intratumoral microdialysis placement post high-grade glioma resection or midline glioma biopsy
- To evaluate safety and feasibility of brain interstitial dialysate sampling in glioma participants post abemaciclib administration
- To measure intratumoral vs. systemic concentrations of abemaciclib in glioma participants post abemaciclib administration

### Secondary study endpoints

- To determine the impact of results from abemaciclib PK and PD studies on subsequent treatment and participant outcomes

### Other study parameters

- To measure abemaciclib phosphorylated RB (pRB) and topoisomerase II alpha (TopoIIα, specific for S phase) pharmacodynamic assays to assess CDK4/6 inhibition and cell cycle progression in biopsied tumor tissue
- To measure dexamethasone concentrations in brain interstitial fluid to evaluate passive diffusion of known BBB permeable compound
- To measure urea concentrations in blood and brain interstitial fluid to evaluate in microdialysis catheter performance
- To conduct genomic sequencing of biopsied tumor tissue identifying driver mutations linked to targeted therapies
- To establish participant derived xenograft modeling in rodents using biopsied tumor tissue
- To evaluate median progression free survival with continued abemaciclib with temozolomide therapy
- To evaluate standard pharmacokinetic parameters comparing combined abemaciclib and temozolomide vs. abemaciclib alone vs. historical controls of temozolomide only

#### Study procedures

Blood samples will be taken as described. Dialysate samples will be taken as described. Events (recurrence, progression, adverse, toxicity, etc.) will be registered prospectively.

### Withdrawal of individual subjects

Participants who meet the following criteria should be discontinued from the study:

- Inability to maintain eligibility post-enrollment and prior to start of therapy, in the opinion of the Principal Investigator
- Screen failure
- Participant requests to be withdrawn from study
- Death
- Study is discontinued
- Lost to follow-upCTCAE–common terminolo
- AE

#### Replacement of individual subjects after withdrawal

Patients will be replaced after withdrawal.

#### Premature termination of study

The clinical study will be prematurely terminated if the safety board recommends, based on toxicity, recurrence rates or other in participants.

### Safety reporting

#### Safety considerations

While microdialysis placement in midline region has not been reported to date, there have been several studies utilizing convection enhanced delivery (CED) catheters specifically within the brainstem to provide direct therapies; mostly in pediatric diffuse midline glioma patients [8, 38, 39]. Specifically, while placement of CED and microdialysis catheters are matched, CED uses a higher flow rate (7.5uL/min vs. 0.3-1uL/min) with a larger volume of solute delivery (over a short duration, approximately 1 hour) [40]. Multiple DMG studies have been performed using intratumoral drug infusion via CED catheters. The most common adverse effect of CED use is grade 1 or 2 headaches. Additionally, CED has been shown to cause transient toxicities of mild motor weakness, cranial neuropathies, and cerebral edema; which were attributed to drug infusion and resolved with the use of dexamethasone therapy [38, 39, 41]. Thus, we propose while there do exist risks in placement and sampling of microdialysis catheters, the toxicity profile would likely be minimal and not sustained with the use of concomitant steroid therapy; similarly to CED catheters/infusion.

#### Temporary halt for reasons of subject safety

This study may be temporarily suspended or prematurely terminated if there is sufficient reasonable cause. Written notification, documenting the reason for study suspension or termination, will be provided by the suspending or terminating party to study participants, investigator, funding agency, the Investigational New Drug (IND) or Investigational Device Exemption (IDE) sponsor and regulatory authorities. If the study is prematurely terminated or suspended, the Principal Investigator (PI) will promptly inform study participants, the Institutional Review Board (IRB), and sponsor and will provide the reason(s) for the termination or suspension. Study participants will be contacted, as applicable, and be informed of changes to study visit schedule.

Circumstances that may warrant termination or suspension include, but are not limited to:

- Determination of unexpected, significant, or unacceptable risk to participants
- Demonstration of efficacy that would warrant stopping
- Insufficient compliance to protocol requirements
- Data that are not sufficiently complete and/or evaluable
- Determination that the primary endpoint has been met
- Determination of futility

Study may resume once concerns about safety, protocol compliance, and data quality are addressed, and satisfy the sponsor, IRB and as applicable, Food and Drug Administration (FDA).

#### Adverse events

The following adverse event management guidelines are intended to ensure the safety of each participant while on the study. The descriptions and grading scales found in the revised NCI Common Terminology Criteria for Adverse Events (CTCAE) version 5.0 will be utilized for AE reporting. All appropriate treatment areas should have access to a copy of the CTCAE version 5.0.

Most likely adverse events (>20%) associated with abemaciclib include anemia, diarrhea, nausea, vomiting, fatigue, anorexia, leukopenia, and thrombocytopenia. Abemaciclib (LY2835219) in combination with other agents could cause an exacerbation of any adverse event currently known to be caused by the other agent, or the combination may result in events never previously associated with either agent. The most common adverse events associated with temozolomide (>10%) include alopecia, fatigue, nausea, vomiting, headache, and diarrhea.

### Statistical analysis

#### Sample size calculation

The primary aim of this study is to assess the safety and feasibility of dialysis catheter placement and use. This is intended to be a preliminary trial to investigate safety and feasibility. As such, this trial will initially be limited to 5 evaluable participants, with possible expansion by a later amendment. Initially 2 participants with recurrent cortical high-grade glioma will be enrolled on study to assess safety and feasibility of microdialysis placement and evaluation with real-time pharmacokinetic measurements conducted. Then 3 participants with recurrent diffuse midline glioma will be enrolled on study.

For purposes of this preliminary evaluation, the intra-tumoral microdialysis placement will be considered feasible for a given participant if we are able to place the catheter and obtain analyzable dialysate samples in at least 7 but preferably 10 (80%) or more of the 13 intended time points to evaluate the concentration of agents (participants who are not evaluable by having only 6 or fewer analyzable samples, or for other reasons, will be replaced) with no participants experiencing serious complications with microdialysis insertion/maintenance (no Grade 3 or higher bleeding, infection or neurologic adverse events in any participant). The timing of the usable dialysate samples will also determine if the results are adequately spread out over the intended time interval to demonstrate a feasible outcome for that participant. If so, this will be considered sufficient for purposes of this preliminary, exploratory study. If the placement was done with no serious complications in any participant, and if adequate, usable samples are able to be obtained from the microdialysis catheter in at least 4 of the 5 participants, then further use of this device may be considered in this trial by an amendment, or in a subsequent study, either of which would include a formal power analysis to justify the number of participants to enroll on the basis of a defined primary endpoint.

Exploratory aims of the study are to conduct genomic analysis from tissue and pharmacokinetic studies during maintenance therapy. Descriptive statistics will be obtained (no interventions yielding comparison of participant groups). As such, no formal power analysis will be performed related to the exploratory endpoints. Priority for tissue studies 1) pathology, 2) molecular profiling, 3) pharmacology, 4) PDX modeling. Given the small study population, clinical data will be presented as median with range and categorical variables presented as proportions (%). All PK and PD samples will be batched until further analysis. It is expected that up to 1 year may be required to enroll up to 5 evaluable participants. To allow for a small number of inevaluable participants to be replaced, the accrual ceiling will be set at 7 participants.

#### Outcome assumptions

For purposes of this preliminary evaluation, the intra-tumoral microdialysis placement will be considered feasible for a given participant if we are able to place the catheter and obtain analyzable dialysate samples in at least 7 but preferably 10 (80%) or more of the 13 intended time points to evaluate the concentration of agents (participants who are not evaluable by having only 6 or fewer analyzable samples, or for other reasons, will be replaced) with no participants experiencing serious complications with microdialysis insertion/maintenance (no Grade 3 or higher bleeding, infection or neurologic adverse events in any participant). The timing of the usable dialysate samples will also determine if the results are adequately spread out over the intended time interval to demonstrate a feasible outcome for that participant. If so, this will be considered sufficient for purposes of this preliminary, exploratory study. If the placement was done with no serious complications in any participant, and if adequate, usable samples are able to be obtained from the microdialysis catheter in at least 4 of the 5 participants, then further use of this device may be considered in this trial by an amendment, or in a subsequent study, either of which would include a formal power analysis to justify the number of participants to enroll on the basis of a defined primary endpoint.

#### Analysis of endpoints

Exploratory aims of the study are to conduct genomic analysis from tissue and pharmacokinetic studies during maintenance therapy. Descriptive statistics will be obtained (no interventions yielding comparison of participant groups). As such, no formal power analysis will be performed related to the exploratory endpoints. Priority for tissue studies 1) pathology, 2) molecular profiling, 3) pharmacology, 4) PDX modeling. Given the small study population, clinical data will be presented as median with range and categorical variables presented as proportions (%). All PK and PD samples will be batched until further analysis.

## Ethical considerations

### Regulation statement

All Clinical Data and Results and Raw Data will be collected, used and disclosed consistent with all applicable federal statutes and regulations for the protection of human subjects, including, if applicable, the *Standards for Privacy of Individually Identifiable Health Information* set forth in 45 C.F.R. Part 164. Data will be stored according to HHS, FDA regulations and NIH Intramural Records Retention Schedule as applicable.

### Recruitment and consent

This protocol may be abstracted into a plain language announcement posted on NIH websites and on NIH social media platforms. Participant retention is completed through good relationships between the treating physician, research team, participant and family.

Participants will be offered co-enrollment on protocol 10-C-0086: Comprehensive Omics Analysis of Pediatric Solid Tumors and Establishment of a Repository for Related Biological Studies.

Participants will be offered co-enrollment on protocol NCI-11-C-0242: Collection of Blood from Patients with Cancer for Analysis of Genetic Differences in Drug Disposition.

The informed consent document will be provided as a physical or electronic document to the participant or consent designee(s) as applicable for review prior to consenting. A designated study investigator will carefully explain the procedures and tests involved in this study, and the associated risks, discomforts and benefits. In order to minimize potential coercion, as much time as is needed to review the document will be given, including an opportunity to discuss it with friends, family members and/or other advisors, and to ask questions of any designated study investigator. A signed informed consent document will be obtained prior to entry onto the study. Consent will be documented with required signatures on the physical document (which includes the printout of an electronic document sent to participant) or as described below, with a manual (non-electronic) signature on the electronic document. When required, witness signature will be obtained similarly as described for the investigator and participant.

### Benefits and risk assessment

Previous studies evaluating overall response rates post treatment in brain stem gliomas have demonstrated some efficacy in inhibition of the CDK pathway. Abemaciclib (Verzenio®) is a CDK4/6 inhibitor drug that is FDA approved for advanced or metastatic breast cancer. Previous studies using high grade glioma xenograft models treated demonstrated the ability of abemaciclib to cross the BBB, increase survival, and decrease tumor growth when given as a single agent or in combination with temozolomide. The potential benefits from this therapy are identification of targetable genetic alterations which can be matched with potential therapeutics for maintenance therapy post radiation. These therapies have the potential to stabilize or reduce tumor burden post radiation.

There are risks associated with abemaciclib administration and surgical biopsy/resection and microdialysis placement, however we believe the potential benefits of treating targetable tumor PK and PD findings outweigh the risks.

#### Risk of biopsy

Risks associated with cortical/midline biopsy include: bleeding, brain swelling, seizures, stroke, infection, blood clots, and reactions to anesthesia. Routine stereotactic needle biopsy will be performed, using neuronavigation. Over the past 5 years, surgical biopsies of midline gliomas were shown to be performed safely with acceptable risks [42, 43]. Additionally a recent study by Mueller et al, through the Pediatric Neuro-Oncology Consortium (PNOC003), examined whole exome sequencing and RNA sequencing in newly diagnosed DIPG participants to incorporate personalized treatments [44]. In their study of 17 DIPG participants receiving pontine biopsies, nine adverse events were reported to be related to surgery. Of these eight were grade 1 and one participant had worsening of his baseline grade 2 nystagmus to grade 3, that recovered back to baseline within 2 days from the biopsy.

#### Risk of microdialysis catheter placement and sampling measures

Risks of microdialysis will be discussed with the participants, but the research literature indicates this procedure does not present more than minimal additional theoretical risk. Immediate theoretical risk of the surgery for placement would include bleeding, seizure, tissue scarring or infection. A recent study by Badie et al., with microdialysis catheters being implanted in the peritumoral area in brain tumor patients after resection, reported no morbidity from microdialysis catheter placement or sample collection [45]. A study of the use of the CMA 70 catheter in 174 patients revealed no incidents of hemorrhage or infection attributable to the microdialysis catheters. A study of microdialysis in recurrent glioblastoma patients prior to resection of their lesion also showed that in the 8 patients that had catheters implanted, there were no observed complications [18]. A recent article, using the MD 71 catheter, to investigate the cytokine changes in glioblastoma tissue and surrounding brain to radiotherapy [46]. In this study, 11 patients had biopsies, and then microdialysis catheters were placed directly into the tumor, and another catheter was placed 10mm away from the contrast-enhancing tissue. No morbidity was noted from placement of these catheters. In another study, 7 patients with malignant glioma or lymphoma had resections of their tumor, and then two MD 71 catheters placed–one at the tumor resection margin, and one 20mm away from the resection cavity [47]. No morbidity from placement of these catheters was noted in this study. The stereotactic biopsy is being done as a part of the biopsy and microdialysis implantation surgery, and it carries a risk of symptomatic hemorrhage of 1% [48]. Stereotactic biopsy with a frameless neuronavigation system carries a risk of about 1% of inaccurate biopsy [49]. In the event of a serious complication attributed to catheter placement or sampling, no further participants will be recruited/enrolled on study.

#### Risk of abemaciclib

The most common adverse reactions to abemaciclib (≥ 20%) in clinical studies were diarrhea, neutropenia, fatigue, infections, nausea, abdominal pain, anemia, vomiting, alopecia, decreased appetite and leukopenia. The protocol provides for detailed and careful monitoring of all participants to assess for toxicity and the dose escalation scheme is very conservative. Toxicity data from the current dose level will be collected and reviewed to ensure that there were no severe (dose-limiting) toxicities. Furthermore, by just entering the study, participants will be followed very carefully, in an organized coherent manner, which may also benefit their overall healthcare.

#### Risk of temozolomide

The most common adverse reactions (≥10% incidence) are alopecia, fatigue, nausea, vomiting, headache, constipation, anorexia, convulsions, rash, hemiparesis, diarrhea, asthenia, fever, dizziness, coordination abnormal, viral infection, amnesia, and insomnia. The most common Grade 3 to 4 hematologic laboratory abnormalities (≥10% incidence) that have developed during treatment with temozolomide are: lymphopenia, thrombocytopenia, neutropenia, and leukopenia. Allergic reactions have also been reported. See package insert for full details.

#### Risk of microdialysis catheter sampling

There is minimal risk to microdialysis sample retrieval. Microdialysis catheters have been used in clinical patient care for periods of time up to 10 days without increased risk of infection [50]. A recently completed study with the CMA 70 catheter in 12 patients with recurrent glioblastoma patients [30]. These patients received either a biopsy or a surgical resection, and then microdialysis catheters were placed into the wall of the resection cavity or tumor tissue. Levels of 5-FU, the expected metabolite of the initial drug, 5-FC, were measured by microdialysis for a minimum of 8 days, and these measurements continued up to 11 days. No infections (or any other morbidity) were reported in this trial. Given the evidence, we do not believe that there is an increased risk of infection for microdialysis sampling for 2 days.

### Incentives

Enrolled patients will not receive any financial compensation for trial participation.

### Administrative aspects, monitoring and publication

#### Handling and storage of data and documents

Samples will be ordered in a computerized clinical system and tracked through a Clinical Trial Data Management system. Samples will not be sent outside NIH without appropriate approvals and/or agreements, if required. All specimens obtained in the protocol are used as defined in the protocol. Any specimens that are remaining at the completion of the protocol will be stored in the conditions described below. The study will remain open so long as sample or data analysis continues. Samples from consenting participants will be stored until they are no longer of scientific value or if a participant withdraws consent for their continued use, at which time they will be destroyed. If the participant withdraws consent his/her data will be excluded from future distributions, but data that have already been distributed for approved research use will not be able to be retrieved. The PI will record any loss or unanticipated destruction of samples as a deviation.

It is expected that up to 1 year may be required to enroll up to 5 evaluable participants. To allow for a small number of in-evaluable participants to be replaced, the accrual ceiling will be set at 7 participants.

#### Monitoring and quality assurance

The clinical site will perform internal quality management of study conduct, data and biological specimen collection, documentation and completion. An individualized quality management plan will be developed to describe a site’s quality management. Quality control (QC) procedures will be implemented beginning with the data entry system and data QC checks that will be run on the database will be generated. Any enrollment data or data anomalies will be communicated to the site(s) for clarification/resolution.

Following written Standard Operating Procedures (SOPs), the monitors will verify that the clinical trial is conducted and data are generated and biological specimens are collected, documented (recorded), and reported in compliance with the protocol, International Conference on Harmonisation Good Clinical Practice (ICH GCP), and applicable regulatory requirements (e.g., Good Laboratory Practices (GLP), Good Manufacturing Practices (GMP)).

The investigational site will provide direct access to all trial related sites, source data/documents, and reports for the purpose of monitoring and auditing by the sponsor, and inspection by local and regulatory authorities.

The clinical research team will meet on a regular basis (approximately weekly) when participants are being actively treated on the trial to discuss each participant. Decisions about dose level enrollment and dose de-escalation, if applicable, will be made based on the toxicity data profile Adverse events, deviations, response evaluations, and all other study related issues will be reviewed at this time.

All data will be collected in a timely manner and reviewed by the principal investigator. The principal investigator will review adverse event and response data on each participant to ensure safety and data accuracy. The principal investigator will personally conduct or supervise the investigation and provide appropriate delegation of responsibilities to other members of the research staff.

The agents will be supplied by CTEP (Cancer Therapy Evaluation Program), NCI (National Cancer Institute) and used in this protocol is/are provided to the NCI under a Collaborative Agreement (CRADA) between the Eli Lilly and the NCI Division of Cancer Treatment and Diagnosis.

#### Public disclosure and publication policy

Any manuscripts reporting the results of this clinical trial must be provided to CTEP by the Group office for Cooperative Group studies or by the principal investigator for non-Cooperative Group studies for immediate delivery to Collaborator(s) for advisory review and comment prior to submission for publication. Collaborator(s) will have 30 days from the date of receipt for review. Collaborator shall have the right to request that publication be delayed for up to an additional 30 days in order to ensure that Collaborator’s confidential and proprietary data, in addition to Collaborator(s)’s intellectual property rights, are protected. Copies of abstracts must be provided to CTEP for forwarding to Collaborator(s) for courtesy review as soon as possible and preferably at least three (3) days prior to submission, but in any case, prior to presentation at the meeting or publication in the proceedings. Press releases and other media presentations must also be forwarded to CTEP prior to release.

## Discussion

**Fig 3:**
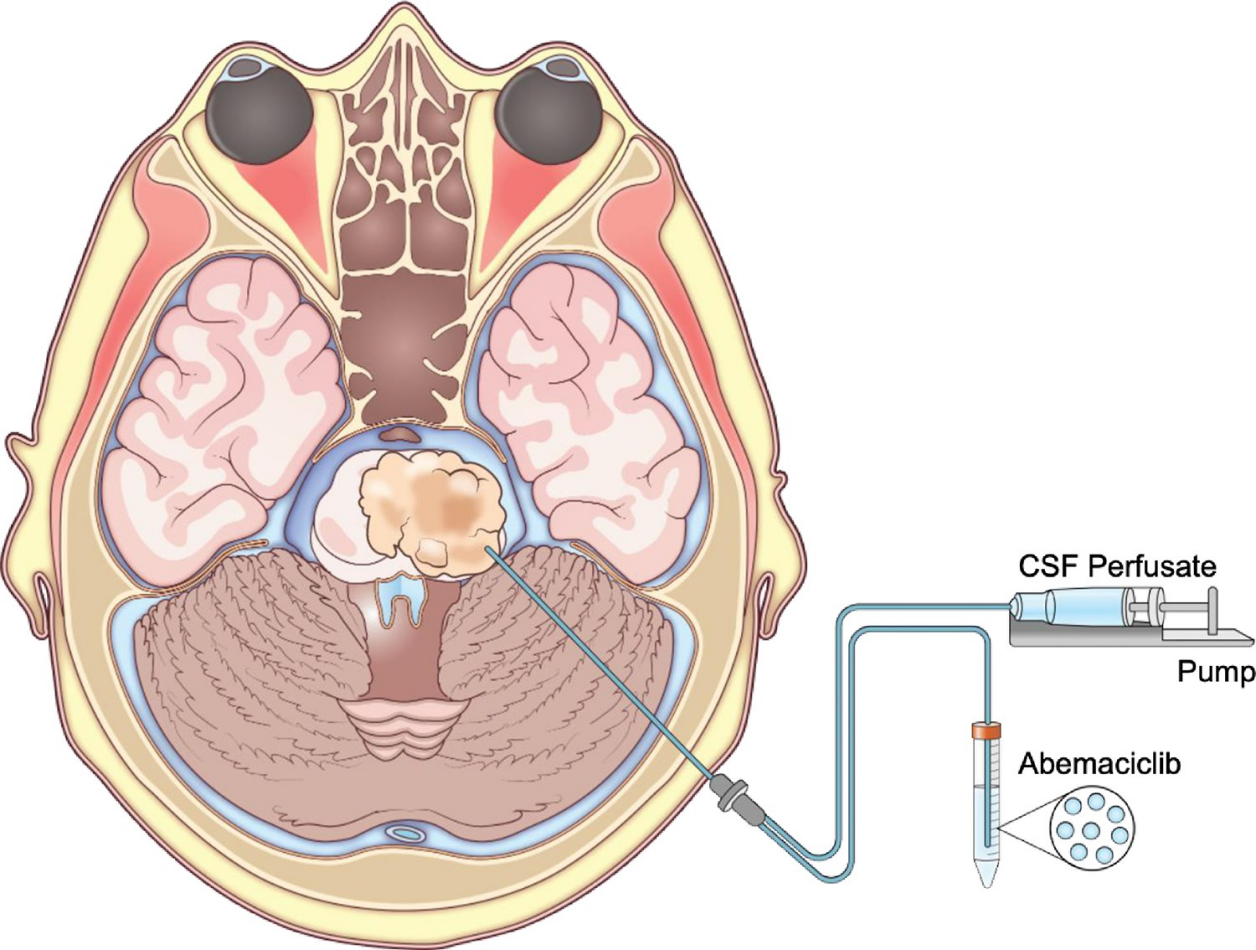
Diffuse midline glioma microdialysis catheter placement. Demonstration of catheter placement in tumor tissue for timed serial extracellular fluid sampling.

We propose to combine findings of neuropharmacokinetics, pharmacodynamics and genomic profiling in high-grade and diffuse midline glioma patients to provide a clearer understanding of the real-time changes within tumoral and peritumoral areas. These results are aimed at more intelligent selection of therapies that take into consideration the tumor biology, tumor microenvironment, and drug properties to measure treatment response. These studies will be the first use of clinical microdialysis as an experimental research tool in DMG patients (Fig 3). The advantage of obtaining tissue in this study will be to have patients receive results of tissue molecular profiling in CLIA certified labs (pharmacology and genomics). Additional benefits of intratumoral microdialysis sampling include data on drug clearance, distribution, and concentration over time. Collectively, these explorations will be used determine efficacy of restarting suitable therapy or recommendation of another agent that is specific to the patients tumoral molecular profile.

Within the US, midline gliomas are not routinely biopsied. However, in the last several years, using modern surgical techniques, biopsy at the time of diagnosis has been performed with acceptable risks (4% mortality rate). This will be a safety and feasibility to evaluate the pharmacokinetic and pharmacodynamic effects following administration of CDK4/6 inhibitor abemaciclib in recurrent midline glioma patients, as measured by a microdialysis catheter. We propose placement of the microdialysis catheter in midline tissue, post biopsy, to 1) assess the neuropharmacokinetic and pharmacodynamic impact of abemaciclib, 2) identify molecularly targetable alterations of tumor tissue and 3) generate PDX models for continued translational studies. Patients will be followed until death to collect information on their clinical course after protocol treatment has ended. The survival rate for these patients is usually less than 2 years from diagnosis; therefore, this is a feasible follow-up period. Detailed genomic studies post mortem have revealed the molecularly heterogeneous landscape of midline gliomas; making the selection of treatment options difficult. Previous studies through the international pediatric brain tumor consortium (PNOC) have been successful in performing biopsies on diffuse midline glioma patients to obtain tumoral genomic data for generation of personalized treatment plans, but failed to provide a survival advantage for such an aggressive disease [44, 51, 52].

The doses of abemaciclib and temozolmide are expected to have an acceptable safety profile based on the experience of abemaciclib with combination dosing (150mg), per Lilly USPI, in various tumor types alone and in combination with temozolamide (200mg/m2). Additionally, there is recently opened (November 2020) Lilly clinical trial examining the combination of abemaciclib (150mg po BID) and temozolomide in young adult participants with solid tumors (including glioblastoma and midline glioma), yet no findings have been reported to date (NCT02644460). Detailed findings of combination studies with abemaciclib and temozolomide have been reported thus far in preclinical models. Raub et al detailed orally dosed abemaciclib (30-50mg/kg) significantly increased survival in a rat orthotopic U87MG xenograft rat models compared with vehicle-treated animals, and efficacy coincided with a dose-dependent increase in unbound plasma and brain exposures in excess of the CDK4 and CDK6 Ki values. Abemaciclib increased survival time of intracranial U87MG tumor-bearing rats similar to TMZ, and the combination of abemaciclib and TMZ was additive or greater than additive [16, 53].

While small participant numbers do not allow for statistical power to detect meaningful differences in pharmacokinetic or genomic profiling, the primary objective of this study is a safety and feasibility. Once these studies demonstrate ease with safely and feasibly sampling brain extracellular fluid in midline gliomas, our intent will be to incorporate and advocate for more use of this microdialysis tool in the larger neuro-oncology field. Intent will be focused on its use in patients with cortical high grade glioma and DMG, to gain this added scientific knowledge regarding drug permeability, metabolite level variations, cytokine expression changes and other CSF biomarker detections in both adults and children with aggressive brain tumors. Too long have clinical teams relied on radiologic imaging and/or clinical exam to determine efficacy of treatment. Alternatively, these study findings will help with linking real-time pharmacokinetic and genomic profiling findings to clinical response, treatment resistance and overall patient outcomes; which will ultimately aid in advancing response predictions related to disease survivorship.

## Supporting information

S1 Checklist SPIRIT-outcomes 2022 checklist (for combined completion of SPIRIT 2013 and SPIRITOutcomes 2022 items)^a^. (PDF)

S2 Checklist TREND checklist. (PDF)

S1 File Clinical protocol. (PDF)

## Abbreviations

DMG: Diffuse Midline Glioma
TMZ: Temozolomide
BBB: Blood-brain barrier
RT: Radiation therapy
CSF: Cerebrospinal Fluid
CNS: Central nervous system
PD: pharmacodynamics
PK: pharmacokinetics
CT: Computed tomography
CDK: cyclin dependent kinase
pRb: phosphorylated Rb
DIPG: diffuse intrinsic pontine glioma
CED: convection enhanced delivery
IND: investigational new drug
IDE: investigational device exemption
IRB: institutional review board
CTCAE: common terminology criteria for adverse event- adverse events
CRADA: Collaborative agreement
CLIA: clinical laboratory improvement amendment
PNOC: pediatric neuro-oncology consortium
AE: adverse events
PDX: patient derived xenograft
HHS: health and human services
PI: principal investigator
QC: quality control
TopoIIα: topoisomerase II alpha
BID: twice a day
Po: per oral
MRI: magnetic resonance imaging
CTEP: Cancer Therapy Evaluation Program
FDA: Food and drug administration
NCI: National cancer institute
NINDS: national institute of neurological disorders and stroke
DCTD: Division of Cancer Treatment and Diagnosis
FDF: financial disclosure form
