# Supplementary material for: Clinical protocol: Feasibility of evaluating abemaciclib neuropharmacokinetics of diffuse midline glioma using intratumoral microdialysis
Source: PLoS One. 2023 Sep 8;18(9):e0291068. doi: 10.1371/journal.pone.0291068 (PMC10490936; doi:10.1371/journal.pone.0291068)
Supplement: S1 File — (PDF) [file pone.0291068.s003.pdf]

Abbreviated Title: Abemaciclib Microdialysis
NCI Protocol #:10444
NIH Protocol #: 22-C-0003
Version Date: 2/6/2023
NCT Number: NCT05413304

Title: Feasibility of Evaluating Abemaciclib Neuropharmacokinetics of Diffuse Midline Glioma Using Intratumoral Microdialysis

NCI Principal Investigator: Sadhana Jackson, M.D. [A-E]
Pediatric Oncology Branch (POB)
Center for Cancer Research (CCR)
National Cancer Institute (NCI)
National Institute of Neurological Disorders and Stroke (NINDS)
Building 10, Room 7D45
National Institutes of Health (NIH)
Bethesda, MD 20892
Phone: 301-594-7037
Email: sadhana.jackson@nih.gov

The study is limited to NCI participation

Drug/Device Name:	Abemaciclib (LY2835219, Verzenio®)	Device for Cerebral Fluid Dialysate Collection	Ashion Analytics GEM ExTra	Temozolomide (Commercial)
NSC#/ IND#:	NSC 783671; IND #158050	NSR Device; IND #158050	NSR Device	Generic
Sponsor:	DCTD, NCI			
Manufacturer:	Eli Lilly and Company	CMA Microdialysis	Ashion Analytics TGEN Clinical Laboratory	NIH CC Pharmacy

Original / Version # 1 / Version Date: 01/28/2022
Amendment / Version # 2 / Version Date: 05/16/2022
Amendment / Version # 3 / Version Date: 08/05/2022
Amendment / Version # 4 / Version Date: 11/07/2022
Amendment / Version # 5 / Version Date: 12/20/2022
Amendment / Version # 6 / Version Date: 02/06/2023

PRÉCIS

Background:

- Diffuse midline gliomas are the most aggressive brain tumors of childhood and young adults, with documented 2 year survival rates of <10%. These tumors are infiltrative midline high-grade gliomas. Treatment failure is due in part to the presence of the blood-brain barrier (BBB), which limits permeability of varied agents.
- Efforts to evaluate drug delivery across the BBB in midline gliomas have been restricted to post biopsy specimens. In comparison, intracerebral microdialysis sampling of cortical tissue has been shown to be a highly effective tool in determining cortical neuropharmacokinetics (brain extracellular fluid penetration, accumulation and excretion) intratumorally and peritumorally in adult brain tumor participants. Microdialysis is underutilized in the adult brain tumor setting to evaluate chemotherapy or targeted therapy permeability.
- Within the US, midline gliomas are not routinely biopsied. However, in the last several years, using modern surgical techniques, biopsy at the time of diagnosis has been performed with acceptable risks (4% mortality rate), with a feasible means to molecularly characterize the tumors; in order to identify potentially druggable targets.
- One of the main attributes of disease progression/proliferation in midline gliomas is associated with dysregulation of the cell cycle. CDKN2A is the primary inhibitory brake on CDK4/6 driven signaling and is commonly deleted in glioblastoma, pancreas, bladder, breast and prostate cancer. The specific CDK4/6 inhibitor, abemaciclib, has FDA approval for the treatment of metastatic breast cancer.
- This is a safety and feasibility study to evaluate pharmacokinetic and pharmacodynamic effects post abemaciclib administration in recurrent high grade glioma participants (cortical and midline tumors). We propose a trial using clinical microdialysis, placed in diffuse midline glioma tissue post biopsy, as an experimental research tool, to assess CNS drug entry and targeted inhibition with abemaciclib. These studies are focused with the overall intent to inform future clinical therapies and preclinical modeling.

Objectives:

- To evaluate safety and feasibility of intratumoral microdialysis placement post high grade glioma resection or midline glioma biopsy
- To evaluate safety and feasibility of brain interstitial dialysate sampling in glioma participants post abemaciclib administration
- To measure intratumoral vs. systemic concentrations of abemaciclib in glioma participants post abemaciclib administration

Eligibility:

- Participants must have recurrent high grade glioma or diffuse midline glioma based on clinical and/or radiologic findings
- Participants with cortical high grade gliomas must have previous intra-operative pathology confirming disease
- Participants must be $\geq$18 and $\leq$ 39 years of age, at time of enrollment
- Ability to swallow tablets/pills
- Must have adequate organ function as per laboratory testing parameters

- Abemaciclib administration must be able to begin no later than 14 days after the date of radiographic diagnosis (by T2 or FLAIR imaging)

Design:

- This is a safety and feasibility study to evaluate tumor pharmacokinetics (PK) and pharmacodynamics (PD) of abemaciclib in recurrent high grade glioma and midline glioma participants in need of surgical resection or biopsy, respectively.
- All participants will take abemaciclib pre-operatively for 4.5 days (9 total doses of abemaciclib) at twice daily dosing.
- A maximally safe surgical resection for cortical high grade glioma or stereotactic needle biopsy for midline glioma will be performed in the OR. Microdialysis insertion (based on participant safety and surgical feasibility) will be performed post-biopsy in the OR and placement will be verified by brain CT.
- Continuous microdialysis sampling will be obtained over the course of the next 48 hours, with subsequent removal of the catheter at the bedside.
- After discharge from NIH inpatient, PK and PD findings will assist in determination of whether the participant will continue to receive abemaciclib therapy. If intratumoral or PK brain dialysate sampling concentrations are >10nmol/L, or PD findings suggest CDK inhibition (decreased expression of Rb and/or topoIIα), then restart of abemaciclib therapy along with temozolomide will be administered for maintenance therapy post resection or biopsy.
- Maintenance therapy will be abemaciclib 150mg po BID x 28 days together with temozolomide 200mg/m^2 po daily x 5 days in 28 day cycles (temozolomide 150mg/m^2 po daily x 5 days for cycle 1). After every 3 cycles, repeat brain MRI's will be obtained to evaluate treatment response and disease progression.
- If a participant starts to exhibit signs of clinical deterioration or radiographic progression, the participant will discontinue maintenance therapy, with ongoing contact for survival approximately every 6 months (+/- 14 days) until death.
- Results of the PK measurements and molecular testing will be provided to the participant's home oncologist to provide for assistance with directed therapy decisions (outside of this investigational trial).
- We propose to evaluate 5 participants. The accrual ceiling will be set at 7 participants.

SCHEMA

Figure 1.

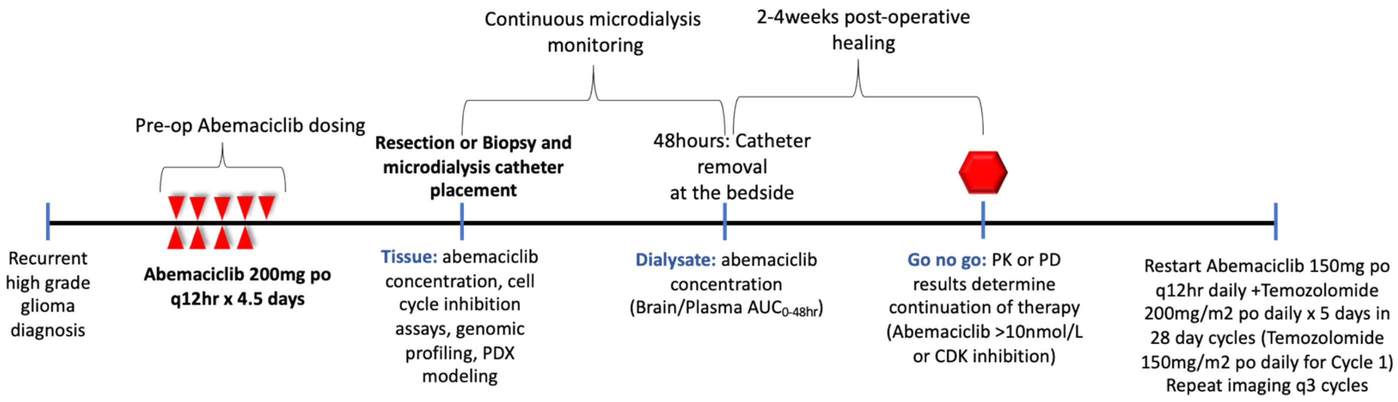

TABLE OF CONTENTS

PRÉCIS 2

SCHEMA 4

TABLE OF CONTENTS 5

1 OBJECTIVES 8
- **1.1** Primary Objectives 8
- **1.2** Secondary Objectives 8
- **1.3** Exploratory Objectives 8

2 BACKGROUND 8
- **2.1** Diffuse Midline Glioma and Intracerebral Microdialysis 8
- **2.2** Abemaciclib 11
- **2.3** Rationale 15
- **2.4** Correlative Studies Background 16

3 PARTICIPANT SELECTION 17
- **3.1** Eligibility Criteria 17
- **3.2** Exclusion Criteria 19
- **3.3** Inclusion of Women and Minorities 20
- **3.4** Recruitment Strategies 20
- **3.5** Screening Evaluation 20

4 PARTICIPANT REGISTRATION AND STATUS UPDATE PROCEDURES 21
- **4.1** Investigator and Research Associate Registration with CTEP 21
- **4.2** Site Registration 23
- **4.3** Participant Enrollment 25
- **4.4** Participant Registration 25
- **4.5** General Guidelines 26
- **4.6** Treatment Assignment Procedures 26

5 BIOMARKER, CORRELATIVE, AND SPECIAL STUDIES 26
- **5.1** Summary Table for Specimen Collection 26
- **5.2** Summary Table for Research Biopsies 28
- **5.3** Specimen Procurement Kits and Scheduling 29
- **5.4** Specimen Storage, Tracking and Disposition 29
- **5.5** Specimen Tacking System Instructions 29
- **5.6** Specimen Collection 32
- **5.7** Shipping of Specimens from Clinical Site to Other Laboratories 33
- **5.8** Biomarker Plan 34
- **5.9** Integral Laboratory or Imaging Studies 37
- **5.10** Investigational Device Information 37
- **5.11** Integrated Correlative Studies 39
- **5.12** Exploratory/Ancillary Correlative Studies 39

6 TREATMENT PLAN 39
6.1 Agent Administration 40
6.2 General Concomitant Medication and Supportive Care 43
6.3 Duration of Therapy/Off treatment Criteria 44
6.4 Duration of Follow-Up 44

7 Dosing Delay/Dose Modifications 45
7.1 Gastrointestinal Toxicity 45
7.2 Hepatotoxicity 46
7.3 Renal Toxicity 49
7.4 Hematologic Toxicity 49
7.5 Nonhematologic Toxicities 50
7.6 Interstitial Lung Disease/ Pneumonitis Events 50
7.7 Venous Thromboembolic Events 51
7.8 Cost and Compensation 51

8 CRITERIA FOR REMOVAL FROM PROTOCOL THERAPY AND OFF STUDY CRITERIA 52
8.2 Lost to Follow-up 52

9 Pharmaceutical Information 52
9.1 CTEP IND Agent(s) 52
9.2 Commercial Agent 56

10 STATISTICAL CONSIDERATIONS 56
10.1 Statistical Design/Endpoints 56
10.2 Sample Size/Accrual Rate 58

11 ADVERSE EVENTS: LIST AND REPORTING REQUIREMENTS 59
11.1 Comprehensive Adverse Events and Potential Risks Lists(s) (CAEPRs) 59

12 Study Calendar 61

13 Measurement of Effect 61
13.1 Toxicity Criteria 61

14 NIH REPORTING REQUIREMENTS/DATA AND SAFETY MONITORING PLAN 61
14.1 Definitions 61
14.2 OHSRP Office of Compliance and Training/ IRB Reporting 62
14.3 NCI Clinical Director Reporting 62

15 STUDY OVERSIGHT AND DATA REPORTING/REGULATORY REQUIREMENTS 62
15.1 Study Oversight 62
15.2 Data Reporting 63
15.3 Data Quality Portal 64
15.4 NIH Required Data and Safety Monitoring Plan 65

15.5 Collaborative Agreements Language 65

16 DATA COLLECTION AND EVALUATION 67
16.1 Data Collection 67
16.2 Genomic Data Sharing Plan....68
16.3 Incidental/Secondary Findings Disclosure Procedure 68
16.4 Adverse Event Characteristics 68
16.5 Expedited Adverse Event Reporting 69
16.6 Routine Adverse Reporting 71
16.7 Pregnancy 71
16.8 Secondary Malignancy 72
16.9 Second Malignancy 72

17 HUMAN SUBJECTS PROTECTIONS 72
17.1 Rationale For Participant Selection 72
17.2 Participation of Children....72
17.3 Participation of Participants Unable to Give Consent 72
17.4 Risk/Benefits Assessment....73
17.5 Consent Process and Documentation....76

18 REGULATORY AND OPERATIONAL CONIDERATIONS 77
18.1 Study Discontinuation and Closure 77
18.2 Quality Assurance and Quality Control....77
18.3 Conflict of Interest Policy....77
18.4 Confidentiality and Privacy 78

19 REFERENCES 78

20 APPENDICES 84
20.1 APPENDIX A: Performance Status Criteria 84
20.2 APPENDIX B: Participant Clinical Trial Wallet Card....85
20.3 APPENDIX C: PK Collection Form 86
20.4 APPENDIX D: Ashion Analytics Sample Genomics Report....89
20.5 APPENDIX E: Study Calendar 91

1 OBJECTIVES

1.1 Primary Objectives

1.1.1 To evaluate the safety and feasibility of intra-tumoral microdialysis placement post high grade glioma resection or midline glioma biopsy

1.1.2 To evaluate the safety and feasibility of brain interstitial dialysate sampling in glioma participants post abemaciclib administration

1.1.3 To measure intratumoral vs. systemic concentrations of abemaciclib in glioma participants post abemaciclib administration

1.2 Secondary Objectives

1.2.1 To conduct long term follow-up with respect to impact of abemaciclib treatment, pharmacokinetic (PK) and pharmacodynamic (PD) studies on subsequent treatment and survival

1.3 Exploratory Objectives

1.3.1 Measure abemaciclib phosphorylated RB (pRB) and topoisomerase II alpha (TopoIIα, specific for S phase) pharmacodynamic assays to assess CDK4/6 inhibition and cell cycle progression in biopsied tumor tissue

1.3.2 Measure dexamethasone concentrations in brain interstitial fluid to evaluate passive diffusion of known blood-brain barrier (BBB) permeable compound

1.3.3 Measure urea concentrations in blood and brain interstitial fluid to evaluate in microdialysis catheter performance

1.3.4 Conduct genomic sequencing of biopsied tumor tissue identifying driver mutations linked to targeted therapies

1.3.5 Establish participant derived xenograft modeling in rodents using biopsied tumor tissue

1.3.6 Evaluate median progression free survival with continued abemaciclib with temozolomide therapy

1.3.7 Evaluate standard pharmacokinetic parameters comparing combined abemaciclib and temozolomide vs. abemaciclib alone vs. historical controls of temozolomide only

2 BACKGROUND

2.1 Diffuse Midline Glioma and Intracerebral Microdialysis

Diffuse midline gliomas compose a rare subset of adult diffuse gliomas that often harbor histone H3 mutations (H3 K27M mutations). Recent studies have demonstrated median survival to be 19.6 months and 25 months in patients who received radiotherapy with or without temozolomide. Adults harboring H3 K27M-mutant gliomas predominate in patients aged < 40 years, and while these gliomas are rare in the entire population of adult IDH wild-type diffuse gliomas they are frequent among the subset of tumors found within the midline.(1-4) These midline tumors are often pathologically distinguished, infiltrative high-grade (malignant) gliomas. Treatment failure is due in part to the presence of the blood-brain barrier (BBB), which limits permeability of varied agents.(5-8) Sadly, midline gliomas grow diffusely and infiltrate critical midline features including

but not limited to the brainstem and thalamus, making complete surgical resection impossible. Standard therapy is radiation therapy, administered over approximately 6 weeks. Despite a number of investigational trials for adults with midline gliomas, no therapy except radiation therapy has ever demonstrated any anti-tumor effect or significant improvement in outcome; additionally, the beneficial anti-tumor effects of radiation therapy are short-lived. Over the past three decades, many clinical trials have explored the use of various chemotherapeutic agents for midline gliomas (including diffuse intrinsic pontine glioma), employing conventional cytotoxic agents, high-dose chemotherapy strategies, chemo-radiotherapy, and molecularly targeted agents. However, no chemotherapeutic agent has *ever* demonstrated significant efficacy against midline glioma with or without H3K27M mutations.(9, 10)

Efforts to evaluate drug delivery across the BBB in midline gliomas have been restricted to post biopsy specimens. In comparison, intracerebral microdialysis sampling of cortical tissue has been shown to be a highly effective tool in determining cortical neuropharmacokinetics (brain extracellular fluid penetration, accumulation and excretion) intratumorally and peritumorally in adult brain tumor patients.(11-19) Microdialysis is a means to measure varied drugs and solutes within brain interstitial fluid versus plasma concentrations; with limitations in evaluating intracellular concentrations. This surgical procedure has been successfully and safely performed at the NIH in adult glioblastoma patients with cortical lesions.(20, 21) To date, this technology has been used widely for traumatic brain injury, and neurologic disorders to monitor metabolites, proteins and CNS drug levels.(17, 22-29) However, it is underutilized in adults and has *never* been utilized in the midline glioma setting to evaluate chemotherapy or targeted therapy permeability.

While microdialysis placement in midline region has not been reported to date, there have been several studies utilizing convection enhanced delivery (CED) catheters specifically within the brainstem to provide direct therapies; mostly in pediatric diffuse midline glioma patients.(8, 30, 31) Specifically, while placement of CED and microdialysis catheters are matched, CED uses a higher flow rate (7.5uL/min vs. 0.3-1uL/min) with a larger volume of solute delivery (over a short duration, approximately 1 hour).(32) Multiple DMG studies have been performed using intratumoral drug infusion via CED catheters.. The most common adverse effect of CED use is grade 1 or 2 headaches. Additionally, CED has been shown to cause transient toxicities of mild motor weakness, cranial neuropathies, and cerebral edema; which were attributed to drug infusion and resolved with the use of dexamethasone therapy.(30, 31, 33) Thus, we propose while there do exist risks in placement and sampling of microdialysis catheters, the toxicity profile would likely be minimal and not sustained with the use of concomitant steroid therapy; similarly to CED catheters/infusion.

The tumor biology and drug distribution pattern within the midline region is largely unknown.(34, 35) One of the main challenges of effectively treating DMG includes the ability to reach therapeutic drug exposure at the target site. Drug concentrations in the midline region are often extrapolated from cortical measurements, due to ease of sampling, in an effort to correlate proposed drug entry to treatment responsivity. However, previous studies demonstrate that brain pharmacology properties including drug half-life, hydrophobicity, metabolism, active compound distribution, multi-drug resistance protein substrate qualities, charge and molecular weight collectively make it difficult to understand tissue concentration at one specified time point.(36) To date, all phase 0 DMG studies specifically evaluating brainstem drug concentrations have evaluated biopsied tissue samples, which evaluates only drug entry at one given time and does not account for multiple pharmacologic attributes. Thus, microdialysis is an exquisite tool to evaluate

extracellular fluid concentrations; allowing for extrapolation of intratumoral concentrations. This proposal will specifically measure biopsied tissue and brain interstitial fluid to compare both intracellular and extracellular concentrations over time. Additional information afforded from biopsied tumor tissue for genomic profiling and pharmacodynamic profiling with this pharmacokinetic data, will serve as useful tools in selecting further precision therapies for such an aggressive disease. These studies aim to better understand the complex relationship between DMG tumor biology and CNS drug distribution, in an effort to more intelligently select therapies for future clinical studies.

2.1.1 CDK4/6 inhibitor Abemaciclib in Diffuse Midline Gliomas

One of the main attributes of disease progression/proliferation in DMG gliomas is associated with dysregulation of the cell cycle.(37) The cell cycle is regulated by an ordered progression of reversible phosphorylation events regulated by cyclins and cyclin dependent kinases. Dysregulation of this ordered progression through the cell cycle is a common feature of many cancers and frequent somatic alteration of cyclin D, CDKN2A and CDK4 are common features in multiple tumor types including breast, head and neck tumors, non-small cell lung cancer, melanoma and glioblastoma. CDKN2A is the primary inhibitory brake on CDK4/6 driven signaling and is commonly deleted in glioblastoma, pancreas, bladder, breast and prostate cancer. Given the prevalence of alteration of this core pathway, multiple specific CDK4/6 inhibitors have made rapid progress in clinical trials including palbociclib, ribociclib, and abemaciclib.(38)

Abemaciclib (Verzenio®) is a CDK4/6 inhibitor that is FDA approved for the treatment of metastatic breast cancer.(39) Abemaciclib has a distinct toxicity profile, likely due to its increased specificity for CDK4.(40) Whereas the primary dose limiting toxicity (DLT) of palbociclib and ribociclib is neutropenia, the DLT of abemaciclib was fatigue. In a study of 225 patients with multiple types of cancer, the most common adverse events caused by abemaciclib included fatigue, diarrhea, and renal and bone marrow toxicity.(41) Overall this medication has been safely tolerated with all toxicities reverting to baseline upon discontinuation of the agent. Clinical trials testing CDK4 inhibitors in multiple combinations are currently underway.(38)

Previous studies evaluating overall response rates post treatment in brain stem gliomas have demonstrated some efficacy in inhibition of the CDK pathway. Studies using glioblastoma xenograft models demonstrated the ability of abemaciclib to cross the blood-brain barrier, increase survival, and decrease tumor growth when given as a single agent or in combination with temozolomide. Recent studies demonstrated disease stabilization in 3/17 glioblastoma patients for up to 23 cycles.(41) While it has shown efficacy in a preclinical brainstem glioma model alone and with combination therapy, details of brainstem drug entry pharmacokinetics and pharmacodynamics are still needed.(38, 42, 43) Evaluations of abemaciclib brain permeability have been limited to cerebrospinal fluid (CSF) sampling in metastatic brain tumor patients.(41) This study found CSF concentrations approached plasma concentrations with notable a CSF concentration range of 2.2–14.7 nmol/L. There is currently one open-enrolling Phase I clinical trial using abemaciclib with escalating dosing in children with DIPG or recurrent/refractory solid tumors. This study is being performed to assess the maximum tolerated dosing of abemaciclib in combination with radiation therapy and then for continuous treatment thereafter; for a maximum duration of two years (NCT02644460). NCT02644460 will evaluate a maximum of 4 cohorts of research participants with escalating doses of abemaciclib starting with dose level 1 (80% of adult dose). There is currently five open-enrolling clinical trials using abemaciclib alone or in combination with other agents (NCT02644460, NCT03220646, NCT04391595, NCT04074785,

NCT02981940) in patients with malignant brain tumors. None of these studies utilize microdialysis combined with genomic profiling to determine further treatment allowances.

2.2 ABEMACICLIB

During the cell cycle, the G1 restriction point controls entry into the S phase and is essential for maintaining control of cell division.(44, 45) The cyclin-dependent kinases (CDKs), CDK4 and CDK6, participate in a complex with D-type cyclins to initiate the transition through the G1 restriction point by phosphorylating and inactivating the retinoblastoma (Rb) tumor-suppressor protein. Alterations in this pathway occur frequently in human cancers and involve: 1) loss of CDK inhibitors by mutation or epigenetic silencing, 2) mutation/overexpression of either CDK4 and CDK6 or cyclin D, or 3) inactivation of Rb. These alterations render cells less dependent on mitogenic signaling for proliferation. With the possible exception of those tumors with complete inactivation of Rb, which functions downstream of the CDK4 and CDK6–cyclin D complex, all cancers that involve CDK mutations or over-expression are potentially sensitive to pharmacologic inhibition of CDK4 and CDK6. From a therapeutic standpoint, the goal of inhibiting CDK4 and CDK6 with a small-molecule inhibitor is to prevent cell cycle progression through the G1 restriction point, thus arresting tumor growth.

Abemaciclib (Verzenio®/Verzenios® [LY2835219]) is an inhibitor of CDK4 and CDK6 and was most active against cyclin D1/CDK4 in enzymatic assays (Investigator’s Brochure, 2022). In breast cancer, cyclin D1/CDK4 has been shown to promote phosphorylation of the Rb protein, cell proliferation, and tumor growth. Abemaciclib prevents Rb phosphorylation, blocking progression from G1 into S phase of the cell cycle, leading to suppression of tumor growth in preclinical models following short-duration target inhibition. In estrogen receptor-positive breast cancer cell lines, sustained target inhibition by abemaciclib prevents rebound of Rb phosphorylation and cell cycle re-entry, resulting in senescence and apoptosis. In breast cancer xenograft models, abemaciclib dosed daily without interruption at clinically relevant concentrations—as a single agent or in combination with antiestrogens—resulted in reduction of tumor size.

Abemaciclib has been approved by the Food and Drug Administration (FDA) for the following indications:

- In combination with an aromatase inhibitor as initial endocrinebased therapy for the treatment of postmenopausal women with hormone receptor (HR)-positive, human epidermal growth factor receptor 2 (HER2)-negative advanced or metastatic breast cancer.
- In combination with fulvestrant for the treatment of women with hormone receptor (HR)-positive, human epidermal growth factor receptor 2 (HER2)-negative advanced or metastatic breast cancer with disease progression following endocrine therapy.
- As monotherapy for the treatment of adult patients with HRpositive, HER2-negative advanced or metastatic breast cancer with disease progression following endocrine therapy and prior chemotherapy in the metastatic setting.
- In combination with endocrine therapy (tamoxifen or an aromatase inhibitor) for the adjuvant treatment of adult patients with hormone receptor (HR)-positive, human epidermal growth factor receptor 2 (HER2)-negative, node-positive, early breast cancer at high risk of recurrence and a Ki-67 score ≥ 20% as determined by an FDA-approved test.

2.2.1 Mechanism of Action and Nonclinical Data for Abemaciclib

Abemaciclib mesylate is a potent inhibitor of CDK4 and CDK6 that is selective over other CDKs at the enzyme and cellular level (Investigator's Brochure, 2022). This is demonstrated in Colo-205 cells by potent cellular inhibition of Rb phosphorylation (pSer780, 50% inhibition concentration [IC_{50}] = 120±36 nM) and by exclusive G1 cell cycle arrest (indicated by accumulation of cells with 2N DNA content) up to 6 mcM concentration. Other studies have confirmed and demonstrated that abemaciclib mesylate inhibits CDK4 and CDK6 to induce G1 arrest specifically in Rb-proficient tumors. Using *in vitro* kinase panel screening, abemaciclib mesylate also demonstrates inhibition (IC_{50}<0.3 μM) of the human protein kinases hCDK9, hPIM1, hPIM2, hHIPK2, hDYRK2, GSK3β, hCDK5/P35, and CK2; however, the reversible G1 arrest seen *in vitro* and *in vivo* indicates that the inhibition of CDK4 and CDK6 by abemaciclib mesylate predominates over these other activities.

2.2.2 Nonclinical Summary

The evaluation of abemaciclib in animal models shows that the phenotypic selectivity for G1 arrest, which is observed in cell culture studies, is also observed in the *in vivo* xenograft tumors (Investigator's Brochure, 2022). For such studies, direct biochemical inhibition of CDK4 and CDK6 as well as the phenotypic inhibition of cell cycle progression can be assessed in tumor explants by measuring Rb phosphorylation at serine 780 (pRb), since Rb is a direct target of CDK4 and CDK6 activity. Reduced pRb expression is also a phenotypic marker for G1 arrest at the restriction point (R). The inhibition of topoisomerase IIα (TopoIIα) and phospho-histone H3 (pHH3) provides additional measures for the inhibition of cell cycle progression through the S and M phases, respectively. The inhibition of CDK4 and CDK6 and cell cycle progression *in vivo* by abemaciclib mesylate is evident by the dose-dependent reduction of pRb in Colo-205 xenografts. These effects on Rb also correlate with similar dose-dependent reductions in the other cell markers TopoIIα and pHH3, indicating that inhibition of CDK4 and CDK6 activity by abemaciclib mesylate results in the inhibition of cell cycle progression in the Colo-205 xenograft tumors. This inhibition of pRb and the cell cycle also shows a time-dependency, whereby abemaciclib mesylate treatment results in a sustained pharmacodynamic response in a mouse Colo-205 xenograft model. Specifically, a 50-mg/kg oral dose resulted in ≥50% inhibition of Rb phosphorylation for 1 to 24 hours after dosing. This effect also correlated with an inhibition of cell cycle progression as indicated by the potent suppression of pRb, TopoIIα, and pHH3 observed at 24 hours following dosing. A dose response for inhibition was observed in these studies such that the threshold effective doses for 70% inhibition for pRb and TopoIIα inhibition 24 hours after oral dosing were 14.1 and 14.3 mg/kg, respectively.

Nonclinical studies show that abemaciclib can be used effectively in combination with standard cytotoxic or targeted therapies to improve the efficacy of these agents (Investigator's Brochure, 2022). In particular, combination studies conducted in KRAS-mutant NSCLC models such as NCI-H441 or NCI-H2122 indicated the potential for better antitumor efficacy when agents such as gemcitabine, pemetrexed, DC101 (mouse surrogate of the antivascular endothelial growth factor receptor 2 antibody ramucirumab), or everolimus (mammalian target of rapamycin [mTOR] inhibitor) were given in combination with abemaciclib mesylate. In these studies, the combination therapies, compared to the single-agent treatments, resulted in either a greater inhibition of tumor growth during therapy or in a longer duration of growth inhibition following the cessation of treatment. Additionally, studies in ER+ human breast cancer xenograft models show that abemaciclib can combine effectively with endocrine treatments such as tamoxifen or fulvestrant

to attain greater inhibition of tumor growth when compared to monotherapy with any of these agents.

The ability of abemaciclib and its major active metabolites to inhibit the catalytic activities of cytochrome P450 (CYP) enzymes (CYP3A4, CYP2D6, CYP2C19, CYP2C9, CYP2C8, CYP2B6, and CYP1A2) either directly or in a time-dependent manner was examined in human liver microsomes (Investigator's Brochure, 2022). Although no clinically relevant inhibition of CYPs was observed in human liver microsomes, downregulation of mRNA of CYPs including CYP1A2, CYP2B6, CYP2C8, CYP2C9, CYP2D6, and CYP3A by abemaciclib and/or its major active metabolites was observed in cultured human hepatocytes over a concentration range of 0.05 to 10 mcM. This had no clinically meaningful effect of abemaciclib on the PK of CYP substrates in clinical studies. Abemaciclib can be safely coadministered with drugs which are substrates of CYP enzymes.

The effects of abemaciclib on primary reproductive organs in rats and dogs have been assessed in repeat-dose toxicity studies (Investigator's Brochure, 2022). Cytotoxic effects to the male reproductive tract in rats and dogs indicate that abemaciclib may impair fertility in males. No effects on female reproductive organs were observed.

2.2.3 Summary of Clinical Experience

The PK profile of abemaciclib was studied using data available from a total of 222 patients enrolled in Study I3Y-MC-JPBA (JPBA) (Investigator's Brochure, 2022).

2.2.4 Clinical Pharmacokinetics and Recommended Phase 2 Dose

Abemaciclib absorption is slow, with a median time to maximum plasma concentration (T_{max}) of 8.0 hours (Investigator's Brochure, 2022). The absolute bioavailability of abemaciclib is 45% (90% confidence interval [CI]: 40% to 51%). In the therapeutic dose range of 50 to 200 mg, the increase in plasma exposure (area under the concentration-time curve [AUC]) and maximum plasma concentration (C_{max}) is dose-proportional. Steady state was achieved within 5 days following repeated twice-daily dosing, and abemaciclib accumulated with a geometric mean accumulation ratio of 3.3 (80% coefficient of variance [CV]) and 4.6 (88% CV) based on C_{max} and AUC, respectively.

Abemaciclib was highly bound to plasma proteins in humans (mean bound fraction was approximately 96% to 98%), and the binding was independent of concentration from 152 to 5,066 ng/mL (Investigator's Brochure, 2022). Abemaciclib binds to both human serum albumin and alpha-1-acid glycoprotein. The geometric mean systemic volume of distribution is approximately 747 L (68.6% CV). In patients with advanced cancer, concentrations of abemaciclib and its active metabolites in cerebrospinal fluid (CSF) are comparable to unbound plasma concentrations.

Hepatic metabolism is the main route of clearance for abemaciclib (Investigator's Brochure, 2022). Abemaciclib is metabolized to several metabolites primarily by CYP3A, with formation of N-desethyl abemaciclib representing the major metabolism pathway. Additional metabolites include hydroxyabemaciclib, hydroxy-N-desethylabemaciclib, and an oxidative metabolite. Two other metabolites are active with similar potency as abemaciclib.

The geometric mean hepatic clearance (CL) of abemaciclib was 21.8 L/hours (39.8% CV), and the mean plasma half-life ($t_{1/2}$) for abemaciclib in patients was 24.8 hours (52.1% CV) (Investigator's Brochure, 2022). After a single oral dose of [^{14}C]-abemaciclib, approximately 81% of the dose

was excreted in feces, and 3.4% was excreted in urine. The majority of the dose eliminated in feces were metabolites.

Age, sex, and body weight had no effect on the exposure of abemaciclib in a population PK analysis in patients with cancer (135 males and 859 females; age range 24-91 years; and body weight range 36-175 kg) (Investigator's Brochure, 2022).

In a study (I3Y-MC-JPBU [JPBU]) of the effect of a high-fat meal using the 25% w/w capsule formulation, abemaciclib exposure increased by 27% (90% CI: 1.18, 1.36) based on AUC, and by 35% (90% CI: 1.25, 1.46) based on C_{max} (Investigator's Brochure, 2022). Abemaciclib exposure increased by 13% (90% CI 1.05, 1.22) based on AUC, and by 30% (90% CI: 1.20, 1.40) based on C_{max}. Although the change in AUC and C_{max} in the study was statistically significant, it was small relative to the variability in exposure in the cancer patient population and, therefore, not considered to be clinically relevant. A high-fat meal did not change the interindividual variability in PK. Therefore, abemaciclib can be taken without regard to meals.

In patients with severe hepatic impairment, total abemaciclib unbound exposure increased 2.69-fold, and the abemaciclib half-life increased from 24 to 55 hours (Investigator's Brochure, 2022). The abemaciclib dosing frequency should be reduced to once daily in patients with severe hepatic impairment.

Abemaciclib and its metabolites are not significantly cleared renally (Investigator's Brochure, 2022). Dose adjustment is not necessary in patients with mild or moderate renal impairment. There are no data in patients with severe renal impairment, end-stage renal disease, or in patients on dialysis.

Co-administration of abemaciclib with rifampin, a strong CYP3A inducer, decreased the potency-adjusted unbound AUC of abemaciclib plus its active metabolites by 77% and may lead to reduced activity (Investigator's Brochure, 2022). The percent reduction in the potency-adjusted unbound AUC of abemaciclib plus its active metabolites in the presence of moderate CYP3A4 inducers modafinil, efavirenz, and bosentan was predicted to be 29%, 53%, and 41% respectively, using physiologically based PK modeling. Concomitant use of CYP3A inducers should be avoided. Co-administration of a strong CYP3A inhibitor (clarithromycin) resulted in a 1.7-fold increase in the potency-adjusted unbound AUC of abemaciclib plus its active metabolites in patients with advanced and/or metastatic cancer. Patients are recommended to avoid concomitant use of strong CYP3A inhibitors and use caution with co-administered moderate or weak CYP3A inhibitors (see Section **6.2**).

2.2.5 Clinical Safety Summary

Based on clinical experience with abemaciclib, the adverse events of special interest (AESIs) are neutropenia, infections, diarrhea, hepatic events (increases in aspartate aminotransferase [AST] and alanine aminotransferase [ALT]), venous thromboembolic events (VTEs), and interstitial lung disease (ILD)/pneumonitis. (Investigator's Brochure, 2022). The most common (≥5%) AEs in healthy subjects considered related to abemaciclib were nausea (20.8%), diarrhea (14.2%), vomiting (11.9%), headache (10.9%), and abdominal pain (6.6%). Reasonably anticipated serious AEs (SAEs) in cancer patients include VTEs, hypercalcemia, major cardiovascular events, and infections. Reasonably anticipated SAEs in breast cancer patients treated with abemaciclib and trastuzumab include infusion-related reactions and cardiac dysfunction. For breast cancer patients treated with abemaciclib and pembrolizumab, reasonably anticipated SAEs include immune-related events and infusion-related reactions.

2.2.6 Clinical Results Summary

Abemaciclib was first approved on September 28, 2017 by the FDA for the following indications:

- in combination with fulvestrant for the treatment of women with hormone receptor-positive (HR+), HER2- advanced or metastatic breast cancer with disease progression (PD) following endocrine therapy.
- as monotherapy for the treatment of adult patients with HR+, HER2- advanced or metastatic breast cancer with PD following endocrine therapy and prior chemotherapy in the metastatic setting.

Abemaciclib was approved on February 26, 2018 by the FDA for an additional indication:

- in combination with an aromatase inhibitor as initial endocrine-based therapy for the treatment of postmenopausal women with HR+, HER2- advanced or metastatic breast cancer.

2.3 RATIONALE

Within the US, midline gliomas are not routinely biopsied. However, in the last several years, using modern surgical techniques, biopsy at the time of diagnosis has been performed with acceptable risks (4% mortality rate), with a feasible means to assess pharmacokinetics of abemaciclib and molecularly characterize the tumors; in order to recommend abemaciclib therapy post standard of care radiation treatment and prescribe potentially druggable targets.(46)

This will be a safety and feasibility to evaluate the pharmacokinetic and pharmacodynamic effects following administration of CDK4/6 inhibitor abemaciclib in recurrent midline glioma patients, as measured by a microdialysis catheter. We propose placement of the microdialysis catheter in midline tissue, post resection or biopsy, to 1) assess the neuropharmacokinetic and pharmacodynamic impact of abemaciclib, 2) identify molecularly targetable alterations of tumor tissue and 3) generate PDX models for continued translational studies. Patients will be followed until death to collect information on their clinical course after protocol treatment has ended. The survival rate for these patients is usually less than 2 years from diagnosis; therefore, this is a feasible follow-up period. Detailed genomic studies post mortem have revealed the molecularly heterogeneous landscape of midline gliomas; making the selection of treatment options difficult. Thus, current studies enrolling midline glioma patients, with a presumed diagnosis of DMG based on radiographic imaging, often include molecular profiling of biopsied/resection tumor tissue to help in delineating optimal treatment options.(47-49)

These studies will be the first use of clinical microdialysis as an experimental research tool in -midline glioma patients, to assess CNS drug entry and target inhibition with the overall intent to inform future clinical therapies and preclinical modeling. The advantage of obtaining tissue in this study will be to have patients receive results of tissue molecular profiling in CLIA certified labs (pharmacology and genomics), which can be used for continued treatment at the NIH or recommended treatment options by their home oncologist. Previous studies through the international pediatric brain tumor consortium (PNOC) have been successful in performing biopsies on diffuse midline glioma patients to obtain tumoral genomic data for generation of personalized treatment plans.(49, 50)

2.4 CORRELATIVE STUDIES BACKGROUND

2.4.1 Pharmacodynamics post abemaciclib

Using histochemical staining of tumor tissue, expression of phosphorylated RB (pRB) and topoisomerase II alpha (TopoIIα, specific for S phase) will be assessed. These studies will be performed to evaluate CDK4/6 inhibition and cell cycle progression in tumor tissue. Positive control tissue of breast adenocarcinoma and glioblastoma will be used as comparators of expression to protocol obtained tissue post abemaciclib administration.

2.4.2 Dexamethasone concentration in brain dialysate

It is standard therapy for patients to receive steroid therapy post-surgical resection and biopsy. This agent can increase tumor vascular integrity and easily crosses the blood-brain barrier because of its hydrophobic qualities. Thus, in addition to abemaciclib and its active metabolites, dexamethasone concentrations will be assessed in brain interstitial fluid at timed intervals. These measurements will further evaluate passive diffusion of a known blood-brain barrier (BBB) permeable compound to intracerebral microdialysis.

2.4.3 Blood and brain urea concentration

In an interest to evaluate microdialysis catheter performance, urea concentrations in blood and brain interstitial fluid will be measured at timed intervals-correlating with PK evaluation. These findings will further validate the collection efficiency for dialysate specific pharmacokinetic studies (abemaciclib and dexamethasone).

2.4.4 Genomic mutations

Using TGEN Ashion Analytic analysis, genomic sequencing of tumor tissue will assist in identifying driver mutations. The genomic profiling results will be provided to each participant's home oncologist, with plans to link findings to available targeted therapies. Previous studies with this profiling, specifically in pediatric brainstem glioma patients was found to be feasible.(49)

2.4.5 Patient Derived Xenograft (PDX modeling)

Arnulfo Mendoza, D.V.M will be performing all the PDX modeling studies within the Pediatric Oncology Branch. To date, all phase 0 DIPG studies evaluating brainstem drug concentrations have evaluated biopsied tissue samples, which evaluates only drug entry at one given time and does not account for multiple pharmacologic attributes.

If adequate biopsied tumor tissue is available $2x10^6$ cells will be injected into the brainstem of immunodeficient mice (5uL) using stereotaxic frame set-up. These preclinical studies aim to evaluate the use of human derived brainstem glioma cells to create a xenograft mouse model and then evaluate drug concentrations and survival metrics with varied therapies.

- NSG immunodeficient mice (~min 3 mice per biopsied sample) = 9-15 mice
- Midline glioma cells derived from surgical biopsy specimens (3 midline glioma participants to be enrolled on clinical study)

2.4.6 Progression free survival with maintenance therapy

For all participants that meet PK (>10nM abemaciclib+M2+M18+M20 concentration) or PD (inhibition of pRb or TopoIIα) parameters abemaciclib therapy will be restarted with initiation of combination temozolomide. Repeat labs and physical exam will help to evaluate toleration of combination therapy. Brain imaging every 3 months will assist in assessing for disease response and clinical progression.

2.4.7 Pharmacokinetics during maintenance therapy

With interest to evaluate abemaciclib and temozolomide concentrations during combination therapy, whole blood samples will be obtained on day 5 of every other cycle until disease progression/treatment termination. These studies will help to compare to systemic abemaciclib concentrations from initial 5 day course with medication given alone vs. when given in combination with another cytotoxic agent. These findings will additionally be compared with historical PK studies which examined systemic concentration of temozolomide alone in adult high grade glioma patients.(13, 16)

3 PARTICIPANT SELECTION

If found ineligibile, participants may be considered for re-enrollment and would be required to meet all eligibility criteria at the time of re-enrollment.

3.1 Eligibility Criteria

3.1.1 Participants must have recurrent high grade glioma or midline glioma based on clinical and/or radiologic findings

3.1.2 Participants with cortical high grade gliomas must have previous intra-operative pathology confirming disease

3.1.3 Participants must be ≥ 18 and ≤ 39 years old at the time of enrollment

3.1.4 Ability to swallow tablets/pills

3.1.5 Prior Therapies:

- At least 4 weeks must have elapsed since any major surgeries, with no evidence of infections. Minimally invasive biopsies (outside of the brainstem) and central line placements are not considered major surgeries
- Participants who have received prior treatment with abemaciclib or another specific CDK4/6 inhibitor are not eligible for enrollment (ex., Ribociclib, Palbociclib – list is not all inclusive)
- Participants who received chemotherapy must have recovered (Common Terminology Criteria for Adverse Events [CTCAE] Grade ≤1) from the acute effects of chemotherapy except for residual alopecia or Grade 2 peripheral neuropathy prior to randomization. A washout period of at least 21 days is required between last chemotherapy dose and randomization (provided the participant did not receive radiotherapy).
- Participants who received radiotherapy must have completed and fully recovered from the acute effects of radiotherapy. A washout period of at least 14 days is required between end of radiotherapy and randomization.

3.1.6 Adequate performance scale as defined below:

- Karnofsky ≥50% within 14 days prior to enrollment; see **APPENDIX A**

3.1.7 Adequate organ function within 14 days prior to enrollment as defined below:

- Hematologic Function: Participants must have an absolute neutrophil count ≥ 1500/µl, hemoglobin ≥9 g/dL (transfusion independent, defined as not receiving blood transfusion unless related to trauma or surgeries), and platelets ≥100,000/µl

(transfusion independent, defined as not receiving platelet transfusions unless related to trauma or surgeries)

- Hepatic Function: Participants must have bilirubin within 1.5 x the upper limit of normal for age, with the exception of those with Gilbert syndrome, and AST/ALT within ≤ 3 x upper limit of normal.
- Renal Function: Participants must have a creatinine clearance or radioisotope GFR ≥60ml/min/1.73 m^2 or a normal serum creatinine.
- Cardiac Function: Normal ejection fraction (ECHO or cardiac MRI) ≥ 53% (or the institutional normal); QTC or QTcF ≤ 450 msec.

3.1.8 Willingness to avoid grapefruit or grapefruit juice during abemaciclib administration

3.1.9 Informed Consent: Ability of participant or Legally Authorized Representative (LAR) to understand and the willingness to sign a written informed consent document.

3.1.10 Willingness of participant or LAR to sign a written informed consent document and co-enroll in study 03-N-0164

3.1.11 The effects of abemaciclib on the developing human fetus are unknown, however CDK-inhibiting agents are known to be teratogenic. Temozolomide is a cytotoxic chemotherapeutic agent which is known to be teratogenic. For these reasons, women of child-bearing potential must agree to use a highly effective method of contraception prior to study entry, for the duration of study participation, and for 3 weeks after the last dose of abemaciclib and 6 months after temozolomide. Should a woman become pregnant or suspect she is pregnant while she or her partner is participating in this study, she should inform her treating physician immediately. Men treated or enrolled on this protocol must also agree to use adequate contraception prior to the study, for the duration of study participation.

- A woman is considered to be of childbearing potential if she is postmenarcheal, has not reached a postmenopausal state (≥ 12 continuous months of amenorrhea with no identified cause other than menopause), and has not undergone surgical sterilization (removal of ovaries and/or uterus).
- Woman participants of childbearing potential (WOCBP) must have a negative serum or urine pregnancy test with a sensitivity of at least 25 mIU/mL within 7 days of the first dose of abemaciclib.
- Highly effective contraception include intrauterine devices (IUD), hormonal (birth control pills, injections, implants), tubal ligation, or partner’s vasectomy .
- Cases of pregnancy that occur during maternal exposures to abemaciclib should be reported. If a participant or spouse/partner is determined to be pregnant following abemaciclib initiation she must discontinue treatment immediately. Data on fetal outcomes and breastfeeding are to be collected for regulatory reporting and drug safety evaluation.

3.1.12 Abemaciclib administration must be able to begin no later than 14 days after the date of

radiographic diagnosis (by T2 or FLAIR imaging)

3.2 Exclusion Criteria

3.2.1 Participants who cannot safely undergo a biopsy due to contraindications

3.2.2 Pregnant women, or women who intent to become pregnant during the study, are excluded from this study because of the teratogenic effects of abemaciclib. Because there is an unknown but potential risk for adverse events in nursing infants secondary to treatment of the mother with these agents, breastfeeding should be discontinued if the mother is treated on study.

3.2.3 Serious preexisting medical condition(s) that would preclude participation in this study (for example, interstitial lung disease, severe dyspnea at rest requiring oxygen therapy, severe renal impairment [e.g. estimated creatinine clearance <30ml/min], history of major surgical resection involving the stomach or small bowel that would preclude adequate absorption, or preexisting Crohn's disease or ulcerative colitis or a preexisting chronic condition resulting in baseline Grade 2 or higher diarrhea).

3.2.4 Uncontrolled intercurrent illness including, but not limited to, symptomatic congestive heart failure, unstable angina pectoris, cardiac arrhythmia, active bleeding diatheses or renal transplant, or psychiatric illness/social situations that would limit compliance with study requirements.

3.2.5 Personal history of any of the following conditions: syncope of cardiovascular etiology, ventricular arrhythmia of pathological origin (including, but not limited to, ventricular tachycardia and ventricular fibrillation), or sudden cardiac arrest.

3.2.6 Active systemic bacterial infection (requiring intravenous [IV] antibiotics at time of initiating study treatment), fungal infection, or detectable viral infection (such as known human immunodeficiency virus positivity or with known active hepatitis B or C [for example, hepatitis B surface antigen positive]. Participants with HIV who have adequate CD4 counts and who have no requirement for antiviral therapy will be eligible. **NOTE:** Screening is not required for enrollment.

3.2.7 Requires treatment with strong/moderate CYP3A inhibitors or inducers. Participants receiving any medications or substances that are inducers or strong/moderate inhibitors of CYP3A4 are ineligible. Because the lists of these agents are constantly changing, it is important to regularly consult a frequently-updated medical reference. As part of the enrollment/informed consent procedures, the participant will be counseled on the risk of interactions with other agents, and what to do if new medications need to be prescribed or if the participant is considering a new over-the-counter medicine or herbal product.

3.2.8 Inability to undergo MRI and/or contraindication for MRI examinations following the MRI protocol (see Study Procedure Manual). Prosthesis or orthopedic or dental braces that would interfere with MRI.

3.2.9 Refractory nausea and vomiting that would limit drug administration in the opinion of the Principal Investigator

3.2.10 Known severe hypersensitivity to abemaciclib, temozolomide or any excipient of abemaciclib or temozolomide or history of allergic reactions attributed to compounds of

similar chemical or biologic composition to abemaciclib and temozolomide.

3.2.11 Clinical judgment by the investigator that the participant should not participate in the study

3.3 Inclusion of Women and Minorities

NIH policy requires that women and members of minority groups and their subpopulations be included in all NIH-supported biomedical and behavioral research projects involving NIH-defined clinical research unless a clear and compelling rationale and justification establishes to the satisfaction of the funding Institute & Center (IC) Director that inclusion is inappropriate with respect to the health of the participants or the purpose of the research. Exclusion under other circumstances must be designated by the Director, NIH, upon the recommendation of an IC Director based on a compelling rationale and justification. Cost is not an acceptable reason for exclusion except when the study would duplicate data from other sources. Persons of childbearing potential should not be routinely excluded from participation in clinical research.

3.4 Recruitment Strategies

This protocol may be abstracted into a plain language announcement posted on NIH websites and on NIH social media platforms. Participant retention is completed through good relationships between the treating physician, research team, participant and family.

Participants will be offered co-enrollment on protocol 10-C-0086: *Comprehensive Omics Analysis of Pediatric Solid Tumors and Establishment of a Repository for Related Biological Studies.*
Participants will be offered co-enrollment on protocol NCI-11-C-0242: *Collection of Blood from Patients with Cancer for Analysis of Genetic Differences in Drug Disposition*

3.5 Screening Evaluation

3.5.1 Screening activities performed prior to obtaining informed consent by research nursing

Prior to the subject signing the research consent, participants may be contacted by several means (e.g., email, mail, in person or telephone) for consideration as a potential protocol candidate, as follows:

- Email, written, in person or telephone communications with prospective participants
- Review of existing medical records to include H&P, laboratory studies, etc.
- Review of existing MRI, X-ray, or CT images
- Review of existing photographs or videos
- Review of existing pathology specimens/reports from a specimen obtained for diagnostic purposes.

3.5.2 Screening activities performed after a consent for screening has been signed

The following screening activities will be performed only after the participant has signed the study consent OR the consent for study 01-C-0129 (provided the procedure is permitted on that study) on which screening activities may also be performed. Assessments performed at outside facilities or on another NIH protocol within the timeframes below may also be used to determine eligibility once a participant has signed the consent. Participant information must be entered onto a screening log and for participants not enrolled (i.e., screen failures), a brief reason will be entered onto the screening log.

- Clinical and laboratory studies to determine eligibility must be performed within 14 days prior to enrollment unless otherwise indicated.

- Imaging studies are to be done within 14 days prior to enrollment on the trial. See also Study Calendar provided in **APPENDIX E**. Enrollment is defined as the time when the participant has met all eligibility criteria and is registered with the NCI for the study-specific protocol.

3.5.2.1 Clinical Assessments

- Complete history, including prior and concurrent therapy
- Physical Examination including documentation of measurable disease, performance status (using the Karnofsky scale), height, weight, signs and symptoms and vital signs (i.e., heart rate, temperature, blood pressure, respiratory rate, and O_2 saturation by pulse oximetry)

3.5.2.2 Laboratory Evaluations

- Hematology: Complete blood count with differential and platelets.
- Chemistries: Electrolytes (including sodium, potassium, chloride, CO_2), calcium, phosphorus, magnesium, creatinine, BUN, glucose, AST, ALT, bilirubin (total and direct), total protein, albumin, and amylase and lipase.
- Coagulation: PT/INR and PTT
- Urine or serum pregnancy test for all females of childbearing potential.

3.5.3 Imaging Studies

- Gadolinium-Enhanced Brain MRI (T1 pre- and post-contrast, T2, FLAIR, performed in at least 2 planes; GRE) that be completed within 14 days of protocol enrollment
- Additional brain MRI sequences: DCE-MRI perfusion and diffusion-weighted imaging, when possible

3.5.4 Other Assessments and Procedures

- EKG/Echo: Electrocardiogram and echocardiogram to be performed within 14 days prior to enrollment on the trial.

3.5.5 Screen Failures

Screen failures are defined as participants who consent to participate in the clinical trial but are not subsequently assigned to the study intervention or entered in the study. A minimal set of screen failure information is required to ensure transparent reporting of screen failure participants, to meet the Consolidated Standards of Reporting Trials (CONSORT) publishing requirements and to respond to queries from regulatory authorities. Minimal information includes demography, screen failure details, eligibility criteria, and any serious adverse event (SAE).

4 PARTICIPANT REGISTRATION AND STATUS UPDATE PROCEDURES

4.1 INVESTIGATOR AND RESEARCH ASSOCIATE REGISTRATION WITH CTEP

Food and Drug Administration (FDA) regulations require sponsors to select qualified investigators. National Cancer Institute (NCI) policy requires all individuals contributing to NCI-sponsored trials to register with their qualifications and credentials and to renew their registration annually. To register, all individuals must obtain a Cancer Therapy Evaluation Program (CTEP) Identity and Access Management (IAM) account at https://ctepcore.nci.nih.gov/iam. Investigators and clinical site staff who are significant contributors to research must register in the Registration

and Credential Repository (RCR). The RCR is a self-service online person registration application with electronic signature and document submission capability.

RCR utilizes five person registration types.

- Investigator (IVR): MD, DO, or international equivalent,
- Non Physician Investigator (NPIVR): advanced practice providers (*e.g.*, NP or PA) or graduate level researchers (*e.g.*, PhD),
- Associate Plus (AP): clinical site staff (*e.g.*, RN or CRA) with data entry access to CTSU applications, such as the Roster Update Management System (RUMS), OPEN, Rave, acting as a primary site contact, or with consenting privileges,
- Associate (A): other clinical site staff involved in the conduct of NCI-sponsored trials, and
- Associate Basic (AB): individuals (*e.g.*, pharmaceutical company employees) with limited access to NCI-supported systems.

RCR requires the following registration documents:

**Documentation Required**	**IVR**	**NPIVR**	**AP**	**A**	**AB**
FDA Form 1572	✓	✓			
Financial Disclosure Form	✓	✓	✓		
NCI Biosketch (education, training, employment, license, and certification)	✓	✓	✓		
GCP training	✓	✓	✓		
Agent Shipment Form (if applicable)	✓				
CV (optional)	✓	✓	✓		

An active CTEP-IAM user account with a linked ID.me account (the latter required immediately for new CTEP-IAM accounts, and by July 1, 2023 for all users) is required to access all CTEP and Cancer Trials Support Unit (CTSU) websites and applications. In addition, IVRs and NPIVRs must list all clinical practice sites and Institutional Review Boards (IRBs) covering their practice sites on the FDA Form 1572 in RCR to allow the following:

- Addition to a site roster,
- Selection as the treating, credit, or drug shipment investigator or consenting person in OPEN,
- Ability to be named as the site-protocol Principal Investigator (PI) on the IRB approval, and
- Assignment of the Clinical Investigator (CI) task on the Delegation of Tasks Log (DTL).

In addition, all investigators act as the Site-Protocol PI (Investigator listed on the IRB approval), consenting/treating/drug shipment investigator in OPEN, or as the Clinical Investigator (CI) on the DTL must be rostered at the enrolling site with a participating organization.

Additional information is located on the CTEP website at https://ctep.cancer.gov/investigatorResources/default.htm. For questions, please contact the **RCR Help Desk** by email at RCRHelpDesk@nih.gov.

4.2 Site Registration

This study is supported by the NCI Cancer Trials Support Unit (CTSU).

IRB Approval

As of March 1, 2019, all U.S.-based sites must be members of the NCI Central Institutional Review Board (NCI CIRB) in order to participate in Cancer Therapy Evaluation Program (CTEP) and Division of Cancer Prevention (DCP) studies open to the National Clinical Trials Network (NCTN) and NCI Community Oncology Research Program (NCORP) Research Bases. In addition, U.S.-based sites must accept the NCI CIRB review to activate new studies at the site after March 1, 2019. Local IRB review will continue to be accepted for studies that are not reviewed by the CIRB, or if the study was previously open at the site under the local IRB. International sites should continue to submit Research Ethics Board (REB) approval to the CTSU Regulatory Office following country-specific regulations.

Sites participating with the NCI CIRB must submit the Study Specific Worksheet for Local Context (SSW) to the CIRB using IRBManager to indicate their intent to open the study locally. The NCI CIRB's approval of the SSW is automatically communicated to the CTSU Regulatory Office, but sites are required to contact the CTSU Regulatory Office at CTSURegPref@ctsu.coccg.org to establish site preferences for applying NCI CIRB approvals across their Signatory Network. Site preferences can be set at the network or protocol level. Questions about establishing site preferences can be addressed to the CTSU Regulatory Office by email or calling 1-888-651-CTSU (2878).

Sites using their local IRB or REB, must submit their approval to the CTSU Regulatory Office using the Regulatory Submission Portal located in the Regulatory section of the CTSU website. Acceptable documentation of local IRB/REB approval includes:

- Local IRB documentation;
- IRB-signed CTSU IRB Certification Form; and/or
- Protocol of Human Subjects Assurance Identification/IRB Certification/Declaration of Exemption Form.

Additional Requirements

Additional requirements to obtain an approved site registration status include:

- An active Federal Wide Assurance (FWA) number;
- An active roster affiliation with the Lead Protocol Organization (LPO) or a Participating Organization (PO); and
- Compliance with all applicable protocol-specific requirements (PSRs).

4.2.1 Protocol Specific Requirements For 10444 Site Registration

Upon site registration approval in RSS, the enrolling site may access OPEN to complete enrollments. The enrolling site will select their credentialed provider treating the participant in the

OPEN credentialing screen, and may need to answer additional questions related to treatment in the eligibility checklist.

4.2.2 Submitting Regulatory Documents

Submit required forms and documents to the CTSU Regulatory Office using the Regulatory Submission Portal on the CTSU website.

To access the Regulatory Submission Portal log in to the CTSU members' website, go to the Regulatory section and select Regulatory Submission.

Institutions with participants waiting that are unable to use the Regulatory Submission Portal should alert the CTSU Regulatory Office immediately at 1-866-651-2878 in order to receive further instruction and support.

Delegation of Tasks Log (DTL)
Each site must complete a protocol-specific DTL using the DTL application in the Delegation Log section on the CTSU members' website. The Clinical Investigator (CI) is required to review and electronically sign the DTL prior to the site receiving an Approved site registration status and enrolling participants to the study. To maintain an approved site registration status the CI must re-sign the DTL at least annually and when a new version of the DTL is released; and activate new task assignments requiring CI sign-off. Any individual at the enrolling site on a participating roster may initiate the site DTL. Once the DTL is submitted for CI approval, only the designated DTL Administrators or the CI may update the DTL. Instructions on completing the DTL are available in the Help Topics button in the DTL application and describe DTL task assignments, CI signature, and CTEP registration requirements, as well as include a Master Task List.

The individual initiating the DTL for the site should upload the above listed training documentation when making the task assignment. The designated reviewer will accept or reject the documentation. A note regarding rejection of any training documents will display on the Site DTL Browser next to the task assignment. The DTL cannot be submitted for CI sign-off until the minimum number of individuals is assigned to the task and have met the training requirements.

4.2.3 Checking Site's Registration Status
Site's registration status on the CTSU website.

- Log on to the CTSU members' website
- Click on *Regulatory* at the top of your screen
- Click on *Site Registration,* and
- Enter your site's 5-character CTEP Institution Code and click on Go
    - Additional filters are available to sort by Protocol, Registration Status, Protocol Status, and/or IRB Type.

Note: The status shown only reflects institutional compliance with site registration requirements as outlined above. It does not reflect compliance with protocol requirements for individuals participating on the protocol or the enrolling investigator's status with the NCI or their affiliated networks.

4.3 PARTICIPANT ENROLLMENT

The Oncology Patient Enrollment Network (OPEN) is a web-based registration system available on a 24/7 basis. OPEN is integrated with CTSU regulatory and roster data and with the LPOs

registration/randomization systems or the Theradex Interactive Web Response System (IWRS) for retrieval of participant registration/randomization assignment. OPEN or IWRS will populate the participant enrollment data in NCI's clinical data management system, Medidata Rave.

Requirements for OPEN access:

- A valid CTEP-IAM account and linked ID.me account (ID.me accounts are required for all newly created CTEP-IAM accounts and by July 1, 2023 for all users);
- To perform enrollments or request slot reservations: Must be on an LPO roster, ETCTN corresponding roster, or participating organization roster with the role of Registrar. Registrars must hold a minimum of an Associate Plus (AP) registration type;
- If a DTL is required for the study, the registrars must hold the OPEN Registrar task on the DTL for the site; and
- Have an approved site registration for the protocol prior to participant enrollment.

To assign an Investigator (IVR) or Non-Physician Investigator (NPIVR) as the treating, crediting, consenting, drug shipment (IVR only), or receiving investigator for a participant transfer in OPEN, the IVR or NPIVR must list the IRB number used on the site's IRB approval on their Form FDA 1572 in RCR. If a DTL is required for the study, the IVR or NPIVR must be assigned the appropriate OPEN-related tasks on the DTL.

Prior to accessing OPEN, site staff should verify the following:

- Participant has met all eligibility criteria within the protocol stated timeframes; and
- All participants have signed an appropriate consent form and HIPAA authorization form (if applicable).

Note: The OPEN system will provide the site with a printable confirmation of registration and treatment information. IWRS system also sends an email confirmation of the registration. You may print this confirmation for your records.

Access OPEN at https://open.ctsu.org or from the OPEN link on the CTSU members' website. Further instructional information is in the OPEN section of the CTSU website at https://www.ctsu.org or https://open.ctsu.org. For any additional questions, contact the CTSU Help Desk at 1-888-823-5923 or ctsucontact@westat.com.

4.4 Participant Registration

To register a participant, the following documents should be completed by the research nurse or data manager and sent via secure email to the Study Coordinator (gillesan@mail.nih.gov):

- Copy of required laboratory tests
- Signed participant consent form

4.4.1 Additional registration requirements

Registration and status updates (e.g., when a participant is taken off protocol therapy and when a participant is taken off-study) will take place per CCR SOP ADCR-2, CCR Participant Registration & Status Updates found at: https://ccrod.cancer.gov/confluence/pages/viewpage.action?pageId=73203825.

4.5 General Guidelines

Following registration, participants should begin protocol treatment within *14* days. Issues that would cause treatment delays should be discussed with the Principal Investigator. If a participant does not receive protocol therapy following registration, the participant's registration on the study may be canceled. The Study Coordinator should be notified of cancellations as soon as possible.

4.6 Treatment Assignment Procedures

Cohorts

Number	Name	Description
1	Cortical high grade glioma	Participants with recurrent high grade glioma (2 participants)
2	Diffuse midline glioma	Participants with recurrent diffuse midline glioma (3 participants)

Arms

Number	Name	Description
1	Abemaciclib and microdialysis monitoring	Abemaciclib orally BID for 4.5 days followed by resection or biopsy and microdialysis catheter placement with continuous monitoring for 48 hours post-operative

Arm Assignment

This is an open-label non-randomized study. Participants in Cohort 1 and 2 will be directly assigned to Arm 1.

5 BIOMARKER, CORRELATIVE, AND SPECIAL STUDIES

5.1 Summary Table for Specimen Collection

Time Point	Specimen[3]	Send Specimens To:
**Archival**	• Formalin-fixed paraffin-embedded (FFPE) tumor tissue - block (preferred)[1]<br><br>If a block is not available, then submit:<br>• 1 H&E stained slide (3-5 µm)<br>• 30-50 unstained, uncharged, air-dried slides (10 µm)	NIH Laboratory of Pathology or Clinical Service Program (CSP)
**Surgical Resection/ biopsy** [2]	• Formalin-fixed paraffin-embedded (FFPE) tumor tissue block for Pharmacodynamic staining - RB phosphorylation, topoisomerase II alpha (TopoIIα, specific for S phase) (mandatory) – block (preferred)[1]<br><br>• Minimum 2-3 cores (3.2-4.8mm^3) of tissue or 10 unstained slides for Whole exome sequencing (mandatory)	NIH Laboratory of Pathology<br>Stephen Hewitt<br>hewitts@mail.nih.gov<br><br>Ashion Analytics Laboratory<br>Steve Mastrian<br>smastrian@ashion.com

	• Minimum 2-3 cores (3.2-4.8mm^3) of fresh frozen tissue for Intratumoral concentration of abemaciclib and active metabolites M2, M18 and M20 comparison to systemic concentration (mandatory)	Figg Lab, GMB/NCI<br>William Figg,<br>figgw@mail.nih.gov
	• Blood, 4mL, EDTA purple top tubes for Evaluation of actionable mutations for future treatment options (mandatory)	Ashion Analytics Laboratory<br>Steve Mastrian<br>smastrian@ashion.com
	• Blood, 3mL, K2EDTA (lavender top) tube for pre-surgical systemic concentration of abemaciclib and active metabolites M2, M18 and M20 comparison to tumoral concentration (mandatory)	Figg Lab, GMB/NCI<br>William Figg,<br>figgw@mail.nih.gov
	• Minimum 1-2 cores (1.6-3.2mm^3) of tissue for PDX modeling: 2x10^6 cells will be injected into the brainstem of immunodeficient mice (5uL) using stereotaxic frame set-up	Pediatric Oncology Branch,<br>Arnulfo Mendoza,<br>mendozaa@mail.nih.gov
Timed retrievals	• 1-2µL of brain dialysate fluid in microvials for pharmacokinetic studies of abemaciclib+M2+M20 and dexamethasone (mandatory)- Timed retrievals: approximately 2, 6, 10, 14, 18, 22, 26, 30, 34, 38, 40, 44, and 48 hours post catheter insertion	Figg Lab, GMB/NCI<br>William Figg,<br>figgw@mail.nih.gov
	• Blood, 3mL, K2EDTA (lavender top) tube for systemic concentration of abemaciclib and active metabolites M2, M18 and M20 comparison to tumoral concentration (mandatory) - Timed retrievals: 1, 2, 4, 6, 8, 10, and 24, 48, 72 hours after catheter insertion; Day 5 of every other cycle of abemaciclib+temozolomide maintenance therapy<br>• Blood, 3mL sodium heparin (green top) tube for systemic concentration of temozolomide – Day 5 of every other cycle of abemaciclib+temozolomide maintenance therapy	Figg Lab, GMB/NCI<br>William Figg,<br>figgw@mail.nih.gov
	• Blood, 1mL, K2EDTA (lavender top) tube and 1-2µL of brain dialysate fluid in microvials for urea concentration at 2, 6, 10, 14, 18, 22, 26, 30, 34, 38, 40, 44, and 48 hours post catheter insertion (mandatory)	Jackson, NINDS DTAP lab<br>Sadhana Jackson<br>Sadhana.jackson@nih.gov

[1]For archival tissue, a copy of the corresponding anatomic pathology report must be sent with the tissue and uploaded to Rave. If submitting slides, then slides must be processed in order, and numbered sequentially (*e.g.*, H&E stained slide is created first and labeled 1, unstained slides are then created and numbered 2 – 51).

[2]For new biopsies, the Tissue Biopsy Verification Form (**APPENDIX C**) and a copy of the radiology and/or operative reports from the tissue removal procedure *and* the diagnostic anatomic pathology report must be sent with the tissue to the NIH Pathology Dept.

[3]Tubes and media may be substituted based on availability with the permission of the PI or laboratory investigator.

5.2 Summary Table for Research Biopsies

**Biopsy #:** 1				
**Trial Time Point:** Surgical biopsy/resection				
**IR Biopsy Definition:** Research – No Clinical Impact (All cores from a single biopsy procedure impact research goals, but do not directly impact participant care or benefit the participant.)				
**Core Priority**	**Use in the Trial**	**Biomarker Name(s)**	**Tumor Content Required**	**Post-Biopsy Processing**
4	Exploratory	Tissue for PDX modeling	>50%	Place tumor tissue on wet ice for processing into single cells for intracranial injection

5.3 Specimen Procurement Kits and Scheduling

5.3.1 Specimen Procurement Kits

N/A

5.4 Specimen Storage, Tracking and Disposition

Samples will be ordered in CRIS and tracked through a Clinical Trial Data Management system. Should a CRIS screen not be available, the CRIS downtime procedures will be followed. Samples will not be sent outside NIH without appropriate approvals and/or agreements, if required

All specimens obtained in the protocol are used as defined in the protocol. Any specimens that are remaining at the completion of the protocol will be stored in the conditions described below. The study will remain open so long as sample or data analysis continues. Samples from consenting participants will be stored until they are no longer of scientific value or if a participant withdraws consent for their continued use, at which time they will be destroyed.

If the participant withdraws consent his/her data will be excluded from future distributions, but data that have already been distributed for approved research use will not be able to be retrieved. The PI will record any loss or unanticipated destruction of samples as a deviation. Reporting will be per the requirements in Section **14.2**.

5.5 SPECIMEN TACKING SYSTEM INSTRUCTIONS

5.5.1 Storage of Tissue Specimens in the Laboratory of Pathology

Tissues designated for clinical diagnostics are transported to the Laboratory of Pathology (LP) where they are examined grossly and relevant portions are fixed, embedded in paraffin and sectioned and stained for diagnostic interpretation. Unutilized excess tissue that is not placed in paraffin blocks is stored in formalin for up to three months, in accordance with College of American Pathologists/Joint Commission on Accreditation of Healthcare Organizations (CAP/JCAHO) guidelines, and then discarded. Following completion of the diagnostic workup, the slides and tissue blocks are stored indefinitely in the LP's clinical archives. All specimens are catalogued and retrieved utilizing the clinical laboratory information systems, in accordance with CAP/JCAHO regulations. The use of any stored specimens for research purposes is only allowed when the appropriate IRB approval has been obtained. In some cases, this approval has been obtained via the original protocol on which the participant was enrolled.

5.5.2 Clinical Pharmacology Program (Figg Lab)

5.5.2.1 Sample Data Collection

All samples sent to the Blood Processing Core (BPC) will be barcoded, with data entered and stored in the Labmatrix (aka LabSamples) utilized by the BPC. This is a secure program, with access to Labmatrix limited to defined Figg lab personnel, who are issued individual user accounts. Installation of Labmatrix is limited to computers specified by Dr. Figg. These computers all have a password restricted login screen. All Figg lab personnel with access to participant information are required to complete the Human Subjects Research course.

Labmatrix creates a unique barcode ID for every sample and sample box, which cannot be traced back to participants without Labmatrix access. The data recorded for each sample includes the participant ID, name, trial name/protocol number, time drawn, cycle time point, dose, material type, as well as box and freezer location. Participant demographics associated with the clinical center participant number are provided in the system. For each sample, there are notes associated with the processing method (delay in sample processing, storage conditions on the ward, etc.).

5.5.2.2 Sample Storage and Destruction

Barcoded samples are stored in barcoded boxes in a locked freezer at either -20 or -80°C according to stability requirements. These freezers are located onsite in the BPC and offsite at NCI Frederick Central Repository Services in Frederick, MD. Visitors to the laboratory are required to be accompanied by laboratory staff at all times.

Access to stored clinical samples is restricted. Samples will be stored until requested by a researcher named on the protocol. All requests are monitored and tracked in LabSamples. All researchers are required to sign a form stating that the samples are only to be used for research purposes associated with this trial (as per the IRB approved protocol) and that any unused samples must be returned to the BPC. It is the responsibility of the NCI Principal Investigator to ensure that the samples requested are being used in a manner consistent with IRB approval. Following completion of this study, samples will remain in storage as detailed above. Access to these samples will only be granted following IRB approval of an additional protocol, granting the rights to use the material.

If, at any time, a participant withdraws from the study and does not wish for their existing samples to be utilized, the individual must provide a written request. Following receipt of this request, the samples will be destroyed (or returned to the participant, if so requested), and reported as such to the IRB. The PI will record any loss or unanticipated destruction of samples as a deviation. Reporting will be per the requirements of section **14.2.**

Sample barcodes are linked to participant demographics and limited clinical information. This information will only be provided to investigators listed on this protocol, via registered use of the LabSamples. It is critical that the sample remains linked to participant information such as race, age, dates of diagnosis and death, and histological information about the tumor, in order to correlate genotype with these variables.

5.5.3 Jackson Lab

Study subject samples, collected for the purpose of research under this protocol, may be archived in the Jackson Laboratory. All data associated with archived clinical research samples will be entered into the web-based NCI Labmatrix database, a centralized system with access controlled via centralized login. Access to this database is limited to Dr. Jackson and her research staff, and requires individual logins and passwords. Relevant staff in Dr. Jackson's laboratory will receive annually updated NIH/CIT training and maintain standards of computer security.

The data recorded for each sample may include the subject ID, trial name/protocol number, date drawn/collected, treatment cycle time point, cell source (e.g. peripheral blood, tissue) as well as box and freezer location. All received samples will receive a unique bar code number, which will be added to the sample NCI Labmatrix database. Only this bar code will be recorded on the sample vial and the vials will not be traceable back to subjects without authorized access to the NCI Labmatrix database.

Samples will be stored in freezers at -80°C (sera, plasma, tissue samples) or under liquid nitrogen (cells), according to stability requirements. These freezers are located onsite in Dr. Jackson's laboratory. Access to samples from a protocol for research purposes will be by permission of the Principal Investigator.

For sample pickup, page 102-12077. For immediate help, call 301-385-8054 (Dr. Sadhana Jackson cell). For questions regarding sample pick-up, please email sadhana.jackson@nih.gov. Sample tubes will be provided by the Jackson Lab. Tubes should be labeled with the date/time of sampling, the protocol, and the subject identifier. Dr. Jackson should be notified of when the samples should be picked up.

5.5.4 Clinical Service Program(CSP)

- The Clinical Support Laboratory, Leidos Biomedical Research, Inc.-Frederick, of the Clinical Service Program, processes and cryopreserves samples in support of IRB-approved, NCI clinical trials. All laboratory personnel with access to patient information annually complete the NIH online course in Protection of Human Subjects. The laboratory is CLIA certified for anti-IL15 and certain cytokine measurements, and all laboratory areas operate under a Quality Assurance Plan with documented Standard Operating Procedures that are reviewed annually. Laboratory personnel are assessed for competency prior to being permitted to work with patient samples. Efforts to ensure protection of patient information include:

- The laboratory is located in a controlled access building and laboratory doors are kept locked at all times. Visitors to the laboratory are required to be accompanied by laboratory staff at all times.
- Hard copy records or electronic copies of documents containing patient information are kept in the locked laboratory or other controlled access locations.
- An electronic database is used to store information related to patient samples processed by the laboratory.
- The database resides on a dedicated program server that is kept in a central, locked computer facility.
- The facility is supported by two IT specialists who maintain up to date security features including virus and firewall protection.
- Program access is limited to specified computers as designated by the laboratory director. Each of these computers has a password restricted login screen.
- The database sample entry program itself is accessed through a password protected entry screen.
- The database program has different levels of access approval to limit unauthorized changes to specimen records and the program maintains a sample history.
- Upon specimen receipt each sample is assigned a unique, sequential laboratory accession ID number. All products generated by the laboratory that will be stored either in the laboratory freezers or at a central repository facility are identified by this accession ID.
- Inventory information will be stored at the vial level and each vial will be labeled with both a sample ID and a vial sequence number.
- Vial labels do not contain any personal identifier information.
- Samples are stored inventoried in locked laboratory freezers and are routinely transferred to the NCI-Frederick repository facilities for long term storage.

- Access to stored clinical samples is restricted. Investigators establish sample collections under "Source Codes" and the investigator responsible for the collections, the protocol Principal Investigator, specifies who has access to the collection. Specific permissions will be required to view, input or withdraw samples from a collection.
- Sample withdrawal requests submitted to approved laboratory staff by anyone other than the repository source code owner are submitted to the source code owner for approval. The repository facility will also notify the Source Code holder of any submitted requests for sample withdrawal.
  - It is the responsibility of the Source Code holder (the NCI Principal Investigator) to ensure that samples requested and approved for withdrawal are being used in a manner consistent with IRB approval.
  - The Clinical Support Laboratory does perform testing services that may be requested by clinical investigators including, but not limited to, immunophenotyping by flow cytometry and cytokine testing using ELISA or multiplex platforms.
- When requests are submitted by the NCI investigator for shipment of samples outside of the NIH it is the policy of the laboratory to request documentation that a Material Transfer Agreement is in place that covers the specimen transfer. At a minimum, the lab needs

confirmation that one has been executed or an exception was granted from an office authorized to make such exceptions, e.g. NCI Technical Transfer Center. The laboratory does not provide patient identifier information as part of the transfer process but may, at the discretion of the NCI investigator, group samples from individual patients when that is critical to the testing process.

- The NCI investigator responsible for the sample collection is responsible for ensuring appropriate IRB approvals are in place and that a Material Transfer Agreement has been executed prior to requesting the laboratory to ship samples outside of the NIH.

5.6 SPECIMEN COLLECTION

Additional information regarding sample processing will be included in the Study Procedure Manual.

5.6.1 Blood Collection

- Blood samples for Ashion Analytics Platform will be collected by Dr. Sadhana Jackson's team and sent to Ashion Analytics Lab for GEM ExTra testing. See Section **5.7** for shipping details. For sample pickup, page 102-12077. For immediate help, call 301-385-8054 (Dr. Sadhana Jackson cell). For questions regarding sample pick-up, please email sadhana.jackson@nih.gov.

- Blood samples for PK will be processed and stored in Dr. Figg's Clinical Pharmacology Program (CPP) for sample processing per established techniques. For details regarding the timing of sample collection see Section **6.1.4**. Please e-mail NCIBloodcore@mail.nih.gov at least 24 hours before transporting samples (the Friday before is preferred). For sample pickup, page 102-11964. For immediate help, call 240-760-6180 (main blood processing core number) or, if no answer, 240-760-6190 (main clinical pharmacology lab number). For questions regarding sample processing, contact NCIBloodcore@mail.nih.gov. The samples will be processed, barcoded, and stored in Dr. Figg's lab until requested by the investigator.

- Blood and brain dialysate samples for urea concentration will be processed by Dr. Jackson's Developmental Therapeutics and Pharmacology (DTAP), NINDS laboratory. For sample pickup, page 102-12077. For immediate help, call 301-385-8054 (Dr. Sadhana Jackson cell). For questions regarding sample pick-up, please email sadhana.jackson@nih.gov. For details regarding the timing of sample collection see Section **6.1.4**. Bioassays Systems QuantiChrom™ Urea Assay Kit will be used to for both blood and dialysate sample evaluation of microdialysis catheter performance.(51, 52)

The blood drawing limits for research purposes are as follows:

- For adult participants: The amount of blood that may be drawn from adult participants for research purposes shall not exceed 10.5 mL/kg or 550 mL, whichever is smaller, over any eight-week period.

5.6.2 Tumor Sample Collection

See Section **6.1.3** for details regarding biopsy/resection collection.

- Tissue samples will be sent to and processed by the NIH Laboratory of Pathology. Pathology analysis and IHC studies and PD studies will be performed by the NIH Lab of Pathology. Stephen Hewitt, NCI Pathology will be leading studies evaluating p-Rb and

topoisomerase II alpha expression in biopsied tumor samples. Known positive control tissue staining of breast carcinoma tissue and malignant glioma tissue will be used to develop a scoring system for high, moderate, low and negative expression.

- Tumor specimen for genomic studies will be sent to the Ashion Analytics Lab for GEM ExTra Platform. Genomic DNA will be extracted from both tumor tissue and blood for library preparation. Tumor specimen will be a minimum of 1.6mm^{3}2- 3 cores (3.2-4.8mm^3) in size. All specimens will be sent overnight at room temperature, unless samples are obtained on a Friday (then they will be kept at 4°C for shipping Monday). Samples will be shipped to the address listed in Section **5.7**.
- Tumor tissue and dialysate samples for PK studies will be sent to Figg Lab

5.7 Shipping of Specimens from Clinical Site to Other Laboratories

5.7.1 Shipping of Specimens to Ashion Analytics

Blood and tumor tissue will be sent overnight at room temperature, unless samples are obtained on a Friday (then they will be kept at 4°C for shipping Monday). Samples will be shipped to:

Ashion Analytics
445 NORTH 5TH STREET
PHOENIX, AZ 85004
Phone number: 844-539-3309

5.8 BIOMARKER PLAN

Priority	Biomarker Name	Assay (CLIA: Y/N)	Use in the Trial (Integral, Integrated, or Exploratory) AND Purpose	Specimens Tested	Collection Time Points	Manda-tory or Optional	Assay Laboratory and Lab PI
**Tissue-based Biomarkers**							
1	Pathology analysis	Immunohistochemistry<br>CLIA: N	Integrated<br>Histology/malignancy detection	FFPE<br>Tumor tissue	Time of tumor resection or biopsy	M	Immunohistochemistry<br>NIH Laboratory of Pathology<br>or<br>Clinical Services Program (CSP)<br>abhik.ray- chaudhury@nih.gov
2	Abemaciclib pharmacodynamic testing	RB phosphorylation, topoisomerase II alpha (TopoIIα, specific for S phase)<br>CLIA: N	Exploratory<br>Decreased downstream expression after CDK inhibition	FFPE<br>Tumor tissue	Time of tumor resection or biopsy	M	NIH Laboratory of Pathology<br>hewitts@mail.nih. gov
3	Ashion Analytics GEM ExTra Platform	Whole exome sequencing<br>CLIA: Y	Exploratory<br>Evaluation of actionable mutations for future treatment options	Fresh frozen<br>Tumor tissue	Time of tumor resection or biopsy<br>(approximately 2-3 cores or 10 unstained slides)	M	Ashion Analytics Laboratory<br>Steve Mastrian<br>smastrian@ashio n.com
4	Abemaciclib concentration	LC/MSCLIA:Y	Integrated<br>Intratumoral concentration of abemaciclib and active metabolites M2, M18 and M20 comparison to systemic concentration	Fresh frozen<br>Tumor tissue	Time of tumor resection or biopsy	M	Figg Lab, GMB.NCI<br>William Figg,<br>figgw@mail.nih.gov
5	PDX modeling	Tissue processing<br>CLIA: N	Exploratory<br>Continued translational studies	Fresh tumor tissue on ice	Time of tumor resection or biopsy	O	Pediatric Oncology Branch, Arnulfo Mendoza, mendozaa@mail.nih.gov
**Blood-based Biomarkers**							

Priority	Biomarker Name	Assay (CLIA: Y/N)	Use in the Trial (Integral, Integrated, or Exploratory) AND Purpose	Specimens Tested	Collection Time Points	Manda-tory or Optional	Assay Laboratory and Lab PI
1	Ashion Analytics GEM ExTra Platform	Whole exome sequencing<br><br>CLIA: Y	Exploratory<br><br>Evaluation of actionable mutations for future treatment options	Blood, 4mL, EDTA purple top tubes	Time of resection or biopsy	M	Ashion Analytics Laboratory<br><br>Steve Mastrian<br><br>smastrian@ashion.com
2	Abemaciclib concentration	LC/MS<br><br>CLIA: Y	Integrated<br><br>Systemic concentration of abemaciclib and active metabolites M2, M18 and M20 comparison to tumoral concentration	Blood, 3mL, K2EDTA (lavender top) tube	Pre-dose, timed retrievals: time of last dose prior to surgery (less than 14 hours prior to surgery), 1, 2, 4, 6, 8, 10, and 24, 48, 72 hours after catheter insertion, Day 5 of every other maintenance cycle of combination therapy	M	Figg Lab, GMB.NCI<br><br>William Figg,<br><br>figgw@mail.nih.gov
3	Temozolomide concentration	LC/MS<br><br>CLIA: Y	Integrated<br><br>Systemic concentration of temozolomide	Blood, 3mL, Sodium Heparin (green top) tube	Day 5 of every other maintenance cycle of combination therapy	M	Figg Lab, GMB.NCI<br><br>William Figg,<br><br>figgw@mail.nih.gov
4	Urea concentration	Colorimetric assay<br><br>CLIA: N	Exploratory<br><br>Evaluation of catheter performance and drug recovery	Blood, 1mL, K2EDTA (lavender top) tube	Timed retrievals: approximately 2, 6, 10, 14, 18, 22, 26, 30, 34, 38, 40, 44, and 48 hours post catheter insertion	M	Jackson, NINDS DTAP lab<br><br>Sadhana Jackson<br>Sadhana.jackson@nih.gov
**Brain Extracellular Fluid**							

Priority	Biomarker Name	Assay (CLIA: Y/N)	Use in the Trial (Integral, Integrated, or Exploratory) AND Purpose	Specimens Tested	Collection Time Points	Manda-tory or Optional	Assay Laboratory and Lab PI
1	Abemaciclib and dexamethasone concentration and active metabolites M2 and M20 in dialysate	LC/MS<br>CLIA: Y	Integral<br>Evaluation of abemaciclib concentration and active metabolites M2, M18 and M20 in tumor extracellular fluid to establish CNS drug entry	Brain dialysate fluid	Timed retrievals: approximately 2, 6, 10, 14, 18, 22, 26, 30, 34, 38, 40, 44, and 48 hours post catheter insertion	M	Figg Lab, GMB.NCI<br>William Figg,<br>figgw@mail.nih.gov
2	Urea concentration in dialysate	Colorimetric assay<br>CLIA: N	Exploratory<br>Evaluation of catheter performance and drug recovery	Brain dialysate fluid	Timed retrievals: approximately 2, 6, 10, 14, 18, 22, 26, 30, 34, 38, 40, 44, and 48 hours post catheter insertion	M	Jackson, NINDS DTAP lab<br>Sadhana Jackson<br>Sadhana.jackson@nih.gov

5.9 INTEGRAL LABORATORY OR IMAGING STUDIES

5.9.1 Abemaciclib and dexamethasone concentration in dialysate

5.9.1.1 Specimen(s) Receipt and Processing: Clinical Pharmacology Program (Figg Lab)

5.9.1.2 Site(s) Performing Correlative Study: NIH

5.10 INVESTIGATIONAL DEVICE INFORMATION

5.10.1 Cerebral Fluid Dialysate Collection (NSR Device)

Cerebral fluid dialysate shall be collected with device components purchased from CMA Micro Dialysis AB, Sweden (US sales office in Chelmsford, MA) and shall be utilized as investigational use only. MicroDialysis AB distributes the CMA Cerebral Tissue Monitoring System with a 510(k)/device number of K060554 with an intended use to measure intracranial glucose, lactate, and pyruvate. Components of the CMA Cerebral Tissue Monitoring System include the 70 Brain Microdialysis Catheter, 106 Microdialysis Pump and artificial Perfusion Fluid CNS, and microvials. This study will replace the 106 Microdialysis Pump with a fixed flow rate (ref. no. P000003) with the 107 Microdialysis Pump (ref. no. P000127) with an adjustable flow rate. The investigational use of the device components within this protocol is to obtain brain dialysate fluid for the measurement of urea, abemaciclib and dexamethasone. Both the catheter and pump are CE Marked according to the Medical Device Directive (MDD 93/42/EEC).

The use of the microdialysis catheter is an FDA-approved method to sample brain fluid; however, it is not FDA approved to measure urea, abemaciclib and dexamethasone levels, and therefore is considered experimental in this study (Investigational Device).

Significant Risk Criteria	Applicable to current study	Justification
Is an implant	No*	The device is introduced into the participant for less than 72 hours<br>*FDA Definition: Implant is a device that is placed into a surgically or naturally formed cavity of the human body and is intended to remain there for a period of 30 days or more. In order to protect public health, FDA may determine that devices placed in subjects for shorter periods are also implants.<br><br>As stated at the FDA Website-IDE Definitions and Acronyms
Is used in supporting or sustaining human life	No	The device is for measuring intracranial concentration.
Is of substantial importance in diagnosing mitigating or treating disease or preventing impairment of human health	No	The device is not used for diagnostic purposes, and only as an indication that the drug can pass the blood brain barrier, which is critical for identifying effective treatments for diffuse midline glioma, which has a dismal prognosis

Significant Risk Criteria	Applicable to current study	Justification
		with a 2-year survival rate of <10% despite multimodality therapies. No chemotherapeutic agents have demonstrated significant efficacy against diffuse midline glioma in part because the blood brain barrier limits permeability of varied agents. The use of this device to evaluate drug permeability is intended to improve treatment options for individuals with this incurable tumor.
Otherwise poses a risk	Non-Significant	The study is conducted in the participants diagnosed with diffuse midline glioma undergoing surgical biopsy.

5.10.2 ASHION ANALYTICS GEM EXTRA (NSR Device)

Genomic screening will be performed in the CLIA-certified Ashion Analytics TGEN Clinical laboratory using the Ashion Analytics GEM ExTra test. The platform is a proprietary exome platform covering the exons of all known human genes and select promoters and introns relevant to cancer (e.g., TERT promoter, introns that participate in oncogenic fusions). The test also includes whole-transcriptome RNA profiling for fusions and transcriptional variants known to be relevant to cancer (e.g., EGFR vIII). Whole **Ex**ome sequencing identifies point mutations, amplifications and deletions, and translocations/structural variants in the full set of protein-coding genes and in therapeutically actionable non-coding regions. Whole **Tra**nscriptome sequencing identifies the expression of RNA transcripts that may guide therapy (e.g., targetable gene fusions). Tumor/normal exome subtraction helps to clearly distinguish somatic mutation from benign background variation. Results are presented in single, integrated report with a cover page summarizing potentially clinically actionable findings, including on- and off-label therapeutic options, contra-indicated drugs, clinical trial options and markers to help guide checkpoint inhibitor therapy (MSI, TMB, and optional PD-L1 IHC). The sequencing method is aimed at targeted enrichment followed by sequencing on a NovaSeq 6000 (Illumina, Inc.). Methods include analysis of single nucleotide variants, insertions/deletions, structural variants, focal and whole genome copy number. Assay sensitivity is 99.3%, assay specificity is >99%, assay PPV is 99.5%, assay Precision is >95.7% and clinical result turn-around time is 14 days.

Ashion Analytics GEM ExTra test is not FDA approved for this purpose; however, it is being used as an in-vitro device to assess actionable mutations. According to 21 CFR 812.3(m), a significant risk device presents a potential for serious risk to the health, safety and welfare of a participant and meets the significant risk criteria listed in the table below along with the sponsor's conclusions with regard to the applicability of these criteria to the current study. The device has been assessed by the sponsor as non-significant risk per the below.

Significant Risk Criteria	Applicable to current study	Justification
Is an implant	No	The device is not introduced into the participant.
Is used in supporting or sustaining human life	No	The device is used to identify genetic mutations for the purpose of identifying targeted therapies.
Is of substantial importance in diagnosing mitigating or treating	No	While the device is diagnostic, we do not believe in presents a potential for serious risk to the

disease or preventing impairment of human health		health and welfare of the participant. The assessment of gene mutations is only used to help identify potential targeted therapies from which participants enrolling on the study might derive benefit.
Otherwise poses a risk	No	There are no other risks.

5.11 INTEGRATED CORRELATIVE STUDIES

5.11.1 Pathology Analysis, IHC

5.11.1.1 Specimen(s) Receipt and Processing: NIH Laboratory of Pathology or Clinical Services Program (CSP)

5.11.1.2 Site(s) Performing Correlative Study: NIH

5.11.2 Tissue Abemaciclib Concentration (Intratumoral vs. Systemic)

5.11.2.1 Specimen(s) Receipt and Processing: Clinical Pharmacology Program (Figg Lab)

5.11.2.2 Site(s) Performing Correlative Study: NIH

5.11.3 Blood Abemaciclib Concentration (Intratumoral vs. Systemic)

5.11.3.1 Specimen(s) Receipt and Processing: Clinical Pharmacology Program (Figg Lab)

5.11.3.2 Site(s) Performing Correlative Study: NIH

5.11.4 Systemic concentration of temozolomide concentration (blood) LC/MS

5.11.4.1 Specimen(s) Receipt and Processing: Clinical Pharmacology Program (Figg Lab)

5.11.4.2 Site(s) Performing Correlative Study: NIH

5.12 EXPLORATORY/ANCILLARY CORRELATIVE STUDIES

5.12.1 Abemaciclib pharmacodynamic testing (RB phosphorylation, topoisomerase II alpha)

5.12.1.1 Specimen(s) Receipt and Processing: NIH Laboratory of Pathology

5.12.1.2 Site(s) Performing Correlative Study: NIH

5.12.2 Ashion Analytics GEM ExTra Platform- whole exome sequencing; tissue and blood

5.12.2.1 Specimen(s) Receipt and Processing: Ashion Analytics Laboratory

5.12.2.2 Site(s) Performing Correlative Study: NIH

5.12.3 Urea concentration in blood and dialysate

5.12.3.1 Specimen(s) Receipt and Processing: Jackson, NINDS DTAP lab

5.12.3.2 Site(s) Performing Correlative Study: NIH

6 TREATMENT PLAN

This is a safety and feasibility study to evaluate pharmacokinetic and pharmacodynamic effects post abemaciclib administration in recurrent midline glioma participants. All participants will take abemaciclib pre-operatively for 4.5 days (9 total doses of abemaciclib) at twice daily dosing. The

last oral dose will be taken the morning of surgery (less than 14 hours from surgery)- to ensure steady state of drug prior to intracerebral microdialysate sampling. Participants will then undergo a stereotactic needle biopsy (approximately 6 cores to be obtained at a content of $1.6mm^3$ each) or maximally safe surgical resection in the operating room (OR) at the NIH Clinical Center. Microdialysis insertion will be performed post-biopsy in the OR and placement will be verified by brain CT.

While the microdialysis catheter is in place, artificial CSF® (M dialysis, Massachusetts) will be infused and continuous microdialysis sampling will be obtained over the course of 48 hours while in the intensive care unit, after catheter placement. Nursing staff within the ICU will assist with timed blood draws and changing of dialysate microvials. Completed microvials removed from catheter tubing will be placed on dry ice at the bedside for later storage and batched analyses. Microdialysis catheter will be removed at the bedside after approximately 48 hours from insertion; by the neurosurgical team. After removal, the participant will be observed for at least one more day inpatient (can be transferred out of the ICU if clinically stable).

After discharge from NIH inpatient, PK and PD findings will assist in determination of whether the participant will continue to receive maintenance therapy on study. Brain and plasma abemaciclib concentrations in the first 2 participants enrolled on study will have samples analyzed in real-time to ensure feasibility in detecting drug entry. For all participants, results from PK testing (Figg lab) and molecular testing (Ashion Analytics) will be provided to the participant's home oncologist for assistance with directed therapy decisions (outside of this investigational trial). For those participants with intratumoral or PK brain dialysate sampling concentrations >10nmol/L or PD findings suggesting CDK inhibition (decreased expression of Rb and/or topoIIα), maintenance therapy will be initiated, post resection or biopsy after required 2-4 weeks post-surgical healing period. No therapy will be permitted during post-surgical healing. Maintenance therapy will be abemaciclib 150mg po BID x 28 days together with temozolomide $200mg/m^2$ po daily x 5 days in 28 day cycles (temozolomide $150mg/m^2$ po daily x 5 days for cycle 1).(41, 43) Additional plasma pharmacology studies will be performed on day 5 of every other cycle to evaluate effect of combination therapy compared to abemaciclib alone and temozolomide only (historical controls). After every 3 cycles, repeat brain MRI's will be obtained to evaluate treatment response and disease progression. If a participant starts to exhibit signs of clinical deterioration or radiographic progression, the participant will discontinue use of study treatment.

The study team will maintain active contact via phone (every six months) with participants who do not go onto maintenance therapy, or with their home clinical team as part of long-term follow-up to collect information on his/her management of disease and survival post-trial. Once participants on maintenance therapy exhibit disease progression, they will continue long-term follow-up via phone (every six months) as part of long-term follow-up for survival.

6.1 Agent Administration

6.1.1 Abemaciclib

Treatment will be administered on an outpatient basis. Reported adverse events and potential risks are described in Section **11**. Appropriate dose modifications are described in Section **7**. No investigational or commercial agents or therapies other than those described below may be administered with the intent to treat the participant's malignancy.

Abemaciclib will be supplied in 50 milligram (mg) white hypromellose tablets. Abemaciclib will be administered orally twice daily (approximately every 12 hours) continuously for 4.5 days (9 total abemaciclib doses) at a dose of 200mg. During maintenance therapy, abemaciclib will be administered orally twice daily (approximately every 12 hours) in 28-day cycles, at a dose of 150mg. Abemaciclib may be taken with or without food. Participants will be instructed to swallow abemaciclib tablets. Participants will be instructed to take their doses of abemaciclib at approximately the same times every day. The first dose will be taken in the CCR Day Hospital and monitored for toleration up to 2 hours after ingestion. If the participant vomits, the participant will be instructed to take the next dose at its scheduled time. Regarding missed doses, participants will receive instruction that a missed dose may be administered if taken within 2 hours of the scheduled time. If more than one (1) dose is missed pre-operatively, participant will be removed from the study.

All participants will return their blisterpacks of tablets for tablet count to monitor for missed doses per the Study Calendar (**APPENDIX E**). If capsule count indicates that the administered dose deviates by ≥ 10% from the prescribed dose during maintenance treatment, the study staff will review adherence with the participant and initiate interventions to improve adherence.

6.1.1.1 Supportive Care- Abemaciclib

Clinical trial data indicates the majority of participants who receive abemaciclib will develop diarrhea. Therefore, all participants will receive instructions on the prompt management of diarrhea. In the event of diarrhea, supportive care measure should be initiated as early as possible. These include the following:

- At the first sign of loose stools, the participant should initiate antidiarrheal therapy (e.g., loperamide) and notify the investigators for further instructions and appropriate follow-up.
- Participants should also be encouraged to drink fluids (e.g., 8-10 glasses of clear liquids per day).
- Site personnel should assess response of antidiarrheal therapy within 24 hours.
- See Section **7.1** for additional information for diarrhea management and dose modification

6.1.2 Temozolomide

Treatment will be administered on an outpatient basis. Appropriate dose modifications are described in Section **7**. No investigational or commercial agents or therapies other than those described below may be administered with the intent to treat the participant's malignancy.

Participants on maintenance therapy will receive temozolomide capsules to be taken orally (PO) once daily at a dose of 200mg/m^2 on Day 1-5 of each 28-day cycle (temozolomide 150mg/m2 po daily x 5 days for cycle 1). Dosing will be rounded to the nearest 5mg. Participants will be instructed to take temozolomide at approximately the same time each day and to swallow capsules whole and not to open, split, or chew capsules. To lessen nausea, temozolomide should be taken with a glass of water on an empty stomach or at bedtime. If the participant vomits, the participant will be instructed to take the next dose at its scheduled time. Regarding missed doses, participants will receive instruction that a missed dose may be administered if taken within 2 hours of the scheduled time. If more than three (3) doses are missed, participant will be removed from the study.

Participants will be asked to turn in their empty pill bottles/blisterpacks to the medical team after each cycle during maintenance therapy. The empty bottles/blisterpacks will be used to maintain a capsule/tablet count of abemaciclib and temozolomide doses taken. If capsule count indicates that the administered dose deviates by ≥ 10% from the prescribed dose, the study staff will review adherence with the participant and initiate interventions to improve adherence.

6.1.2.1 Supportive care-Temozolomide

Participants treated with temozolomide may experience myelosuppression, including prolonged pancytopenia, which may result in aplastic anemia, which in some cases has resulted in a fatal outcome. In some cases, exposure to concomitant medications associated with aplastic anemia, including carbamazepine, phenytoin, and sulfamethoxazole/trimethoprim, complicates assessment. Prior to dosing each cycle, participants must have an absolute neutrophil count (ANC) greater than or equal to 1.5 x 10^9/L and a platelet count greater than or equal to 100 x 10^9/L. See Section **7.4** for details.

6.1.3 Biopsy

All participants will be co-enrolled in the 03-N-0164: *Evaluation and Treatment of Neurosurgical Disorders* protocol for tissue banking related to surgical resection. This study offers evaluation and treatment of participants with a variety of neurological disorders that may require surgery.

A stereotactic needle biopsy will be performed at the NIH (after 4 days of abemaciclib). At the time of biopsy, approximately 6 small cores (approximately 1.6mm^3 each) will be obtained for further studies (pathology, molecular analysis, and pharmacology). Priority for tissue studies 1) pathology, 2) molecular profiling (2-3 cores or 10 unstained slides needed), 3) pharmacology, 4) PDX modeling. A minimum of 4 cores will be needed to proceed with microdialysis placement.

Participants will be offered (optional) co-enrollment on protocol 10-C-0086: *Comprehensive Omics Analysis of Pediatric Solid Tumors and Establishment of a Repository for Related Biological Studies*, on which additional studies can be performed.

6.1.4 Microdialysis Catheter Insertion and Removal

A CMA 70 Microdialysis Brain Catheter (ref. no. P000050, MDialysis, Chelmsford, MA) with a membrane length of 10mm, shaft length 100mm will be inserted into Microdialysis post-biopsy in the OR; noted potential risks include: bleeding, infection, and tissue scarring. The tip of the Catheter contains a gold thread, which makes it visible on a CT-scan in order to locate the position of the catheter in the brain. After CT imaging confirms proper placement of the microdialysis catheter, the inlet tubing of the catheter will be connected to a portable syringe pump to perfuse artificial CSF (Perfusion Fluid CNS, ref. no. P000151, MDialysis, Chelmsford, MA) at a rate of 0.5 µl/min. The inlet tubing of the catheter will be connected to a CMA 107 Microdialysis Pump (ref. no. P000127, MDialysis, Chelmsford, MA). A microvial will be connected at the end of the outlet tubing to collect perfusate. This microvial can hold up to 200µL of fluid. Dialysate samples for pharmacokinetic and metabolite (urea) analysis will be collected approximately 2, 6, 10, 14, 18, 22, 26, 30, 34, 38, 40, 44, and 48 hours post catheter insertion. ICU nursing will assist with changing of microvials at timed collections. Comparison of urea concentrations systemic vs. intracerebral have been shown are a dependable means to control for the stability of microdialysis catheter function.(51, 52) Along with abemaciclib, dexamethasone (standard post-surgical agent) concentrations will be to evaluate passive diffusion of a known blood-brain barrier (BBB) permeable compound. The microdialysis catheter will be removed at the bedside after approximately 48hours from catheter insertion. Microvials containing the dialysate samples will be placed in a holding rack and put on dry ice within a secure container at the bedside. Thereafter, the dialysate samples should be stored in an ultralow temperature freezer (≤-80°C) until they can be

analyzed by liquid chromatography tandem mass spectrometry (LC/MS/MS) for concentrations of abemaciclib and its metabolites.

Whole blood for pharmacokinetic abemaciclib analysis will be obtained pre-dose, time of last dose pre-surgery (less than 14 hours from time of surgery), and then approximately 1, 2, 4, 6, 8, 10, 24, 48 and 72 hours post catheter insertion. Whole blood for urea analysis will be obtained with PK studies at approximately 2, 6, 10, 14, 18, 22, 26, 30, 34, 38, 40, 44, and 48 hours post catheter insertion. At each sample time point a discard blood volume appropriate for the IV access device must be drawn prior to the sample. Blood (3 mL) will be drawn from a peripheral vein in the participant's arm and collected in green top tubes containing sodium heparin anticoagulant. Tubes will be promptly mixed by gently inverting 6-times, then placed on wet ice, until centrifuged at 1,300 x g for 10 min at 4°C. Samples will be centrifuged for harvesting plasma within 2 hours. Upon centrifugation, the plasma will be separated from the blood cells using a pipette and transferred into an appropriately labeled polypropylene freezer vial. The samples should be processed to plasma within 30 minutes from centrifugation. Plasma will then be stored frozen at -80°C until subsequent batch analysis (**APPENDIX C**). Urea concentration will be evaluated on both blood and dialysate collections (1-2µL/sample minimum). Jackson and Figg labs will be responsible for retrieval of blood and dialysate samples for pharmacokinetic and metabolite analysis per Section **5.6**.

6.1.4.1 Inpatient hospitalization

Close monitoring including q2hr vital signs and neurochecks will be evaluated for up to 24 hours after catheter insertion and then every 4 hours while awake; while participant remains in the intensive care unit for approximately 48 hours. While being monitored as an inpatient, participants will be specifically evaluated by the medical and nursing staff for symptoms and signs of 1) focal neurologic dysfunction, such as weakness, sensory loss, and aphasia; 2) general neurologic dysfunction, such as somnolence; 3) increased intracranial pressure; and 4) infection of the wound, brain, or meninges. During the period of collection, the participant may be mobile within the confines of the collection system. For example, he/she may move from bed to chair or commode and back. Following completion of perfusate collection, the infusion pump use will cease and the microdialysis catheter will be removed percutaneously at the bedside by the neurosurgical team. The entry site of the catheter will be closed with a suture or steristrip as necessary and a clean dressing applied. The participant will be kept in the hospital for observation at least one additional day, until discharged

6.2 General Concomitant Medication and Supportive Care

Participants are recommended to avoid concomitant use of strong CYP3A inhibitors (e.g., voriconazole) and use caution with co-administered moderate (e.g., ciprofloxacin) or weak (e.g., ranitidine) CYP3A inhibitors. If coadministration with a strong CYP3A inhibitor is unavoidable, reduce the abemaciclib dose to 100 mg twice daily or, in the case of ketoconazole, reduce the abemaciclib dose to 50 mg twice daily. In patients who have had a dose reduction to 100 mg twice daily due to adverse reactions, further reduce the abemaciclib dose to 50 mg twice daily. If a participant taking abemaciclib discontinues a strong CYP3A inhibitor, increase the abemaciclib dose (after 3-5 half-lives of the inhibitor) to the dose that was used before starting the strong inhibitor. Avoid grapefruit or grapefruit juice.

Avoid concomitant use of strong CYP3A inducers. Consider alternative agents without CYP3A induction.

6.3 Duration of Therapy/Off treatment Criteria

In the absence of treatment delays due to adverse event(s), treatment may continue until evidence of disease progression during maintenance therapy and completion of the follow-up period.

Off Treatment Criteria:

- Completion of protocol therapy and completed follow-up period
- Intercurrent illness that prevents further administration of treatment
- Unacceptable adverse event(s) (see Section 7)
- Noncompliance with trial treatment or procedure requirements in the opinion of the investigator; such a decision/rationale will be clearly noted in the medical record
- Pregnancy
- Participant decision
- Investigator's decision to withdraw the participant
- Study is discontinued

6.4 Duration of Follow-Up

6.4.1 Safety Follow-Up Visit

The safety follow-up visit (in-person or tele-health) should occur 30 days (+7 days) after last dose of study drug. See Study Calendar (**APPENDIX E**) for a list of assessments to be performed. A comparable evaluation by the participant's primary care physician, with standard safety laboratory studies obtained, will be deemed acceptable to fulfill this required safety follow-up visit, if it is not feasible for the participant to return to the NIH Clinical Center for this visit.

All AEs that occur prior to the Safety Follow-Up Visit should be recorded. Participants with an ongoing, treatment-related AE of Grade >1 will be followed until the resolution of the AE to Grade 0-1, stabilization of the AE in the opinion of the investigator, or until the beginning of a new anti-neoplastic therapy, whichever occurs first. SAEs that occur within 30 days of the end of treatment or before initiation of a new anti-cancer treatment should also be followed and recorded.

6.4.2 Relay of pharmacology and genomic findings

Approximately 4 weeks after surgical biopsy, results from pharmacokinetic testing and genomic profiling will be relayed to each participant's home oncologist. Specifically, Figg lab will evaluate abemaciclib concentration within brain tissue, dialysate fluid and plasma in a CLIA certified lab. If intratumoral or PK brain dialysate sampling concentrations are >10nmol/or PD findings suggesting CDK inhibition, then the participant will restart abemaciclib and start temozolomide therapy for maintenance treatment of 28 day cycles.(41) On the 5th day of every other cycle of combination maintenance therapy, whole blood will be obtained to assess concentrations of abemaciclib and temozolomide (comparing combined abemaciclib and temozolomide vs. abemaciclib alone vs. historical controls of temozolomide only). Additionally, genomic sequencing of biopsied tumor tissue will also be relayed to each participant's home oncologist within 2-4 weeks after surgical biopsy; with a detailed assessment similar to the example in **APPENDIX D.**

6.4.3 Long-Term Follow-Up

Continued long-term follow-up will occur for all participants regardless of whether they go on to receive maintenance combination therapy or not. After completion of trial therapy, a participant

or their local health care provider will be contacted via phone every 6 months (+/- 14 days) until death to obtain clinical information as outlined in the Study Calendar **APPENDIX E**.

7 DOSING DELAY/DOSE MODIFICATIONS

The pre-operative dose of abemaciclib will not be adjusted for lower dosing for drug-related toxicities on this study due to the short duration of therapy (detailed PK findings of abemaciclib based on standard dosing). Any grade 3 or higher toxicity that is deemed attributable to abemaciclib within the first 4.5 days will require drug discontinuation and removal from study. Those participants will be replaced. Any other toxicities seen prior to surgery that make surgery not feasible or unsafe will also require discontinuation of therapy, removal from study and replacement. If grade 3 or higher toxicity is noted during maintenance therapy (abemaciclib combined with temozolomide), then both therapies will be held until symptoms resolve to grade 1-2. After resolution, will restart temozolomide dosing at 150mg/m^2 (or 100mg/m^2 if during cycle 1) with no change in abemaciclib dosing. The participant will be taken off treatment and followed until death if they experience any two Grade 4 adverse events or one Grade 5 adverse event that can be attributed to abemaciclib.

If the toxicity is likely or potentially related to abemaciclib, the appropriate dose reductions for abemaciclib must occur. If it is likely or potentially related to temozolomide, then appropriate dose reductions for temozolomide should be made. If it is not possible to attribute the toxicity to either drug, consider reducing both. If a participant must stop one of the study drugs, then they will be taken off treatment and followed until death.

Management of drug holds, and supportive care will be at the discretion of the PI. If the participant requires discontinuation of the drug due to prolonged grade 3 or higher toxicity, they will be removed from the study protocol directed therapy and monitored closely until toxicities resolve; and then every 6 months until death.

7.1 GASTROINTESTINAL TOXICITY

Clinical trial data indicates the majority of participants who receive abemaciclib will develop diarrhea. Participants will receive instructions at enrollment on the prompt management of diarrhea (see Section **6.1.1.1**). At the first sign of loose stools, start treatment with antidiarrheal agents, such as loperamide (per clinical dosing guidelines). If a participant experiences diarrhea during maintenance therapy that is unrelieved by antidiarrheal therapy, adjust abemaciclib according to the following table (**Table 1**):

Table 1: Dose Modification and Management – Diarrhea

CTCAE Grade	Abemaciclib Maintenance Therapy Dose Modifications
Grade 1	No dose modification is required.
Grade 2	If toxicity does not resolve within 24 hours to ≤Grade 1, suspend dose until resolution. Dose reduction is not required
Grade 2 that persists or recurs after resuming the same dose despite maximal supportive measures	Suspend dose until toxicity resolves to ≤Grade 1. Resume at next lower dose.
Grade 3 or 4 or requires hospitalization	

Dose level reductions should be made in 50 mg increments. For example, if the starting dose of abemaciclib for a study is 150 mg q12 hours, dose reduction 1 would be 100 mg q12 hours, dose reduction 2 would be 50 mg q12 hours.

7.2 HEPATOTOXICITY

Alanine aminotransferase (ALT) and aspartate transaminase (AST) elevation is considered an adverse drug reaction with the use of abemaciclib. ALT/AST will be monitored prior to the start of abemaciclib therapy, approximately 7 days after starting therapy (>48hours post-operative) and 30 (±7) days after last dose of abemaciclib (to be obtained by home institution) and as clinically indicated. ALT/AST will be monitored during maintenance therapy prior to the start of every cycle. If a participant experiences ALT/AST elevation during maintenance therapy, adjust abemaciclib dosing according to the following table (**Table 2**):

Table 2: Dose Modification and Management – Increased ALT/AST

CTCAE Grade	Abemaciclib Maintenance Therapy Dose Modifications
Grade 1 (>ULN-3.0 x ULN)<br>Grade 2 (>3.0-5.0 x ULN)	No dose modification is required.
Persistent or recurrent Grade 2, or Grade 3 (>5.0-20.0 x ULN)	Suspend dose until toxicity resolves to baseline or Grade 1.<br>Resume at next lower dose.
≥Grade 2 (>3.0 x ULN) with total bilirubin >2 x ULN, in the absence of cholestasis	Discontinue abemaciclib
Grade 4 (>20.0 x ULN)	Discontinue abemaciclib

Close Hepatic Monitoring and Evaluation

Liver testing including ALT, AST, alkaline phosphatase (ALP), total bilirubin (TBL), direct bilirubin (D. Bil), and creatine kinase (CK), should be repeated within 2 to 4 days to confirm the abnormality and to determine if it is increasing or decreasing if one 1 or more of the following conditions occur:

If a participant with baseline results of...	develops the following elevations:
ALT or AST <1.5×ULN	ALT or AST ≥5×ULN or<br>ALT or AST ≥3×ULN concurrent with TBL ≥2×ULN
ALT or AST ≥1.5×ULN	ALT or AST ≥3× baseline or

	ALT or AST ≥2× baseline concurrent with TBL ≥2×ULN

If the abnormality persists or worsens, clinical and laboratory monitoring and evaluation for possible causes of abnormal liver tests, should be initiated by the investigator. At a minimum, this evaluation should include physical examination and a thorough medical history, including symptoms, recent illnesses (for example, heart failure, systemic infection, hypotension, or seizures), history of concomitant medications (including over-the-counter, herbal and dietary supplements, history of alcohol drinking and other substance abuse). In addition, the evaluation should include a blood test for prothrombin time (PT-INR); serological tests for viral hepatitis A, B, C, E, autoimmune hepatitis; and an abdominal imaging study (for example, ultrasound or CT scan)

Based on the patient's history and initial evaluation results, further testing should be considered, including tests for hepatitis D virus (HDV), cytomegalovirus (CMV), Epstein-Barr virus (EBV), acetaminophen levels, acetaminophen protein adducts, urine toxicology screen, Wilson's disease, blood alcohol levels, urinary ethyl glucuronide, and blood phosphatidylethanol. Based on the circumstances and the investigator's assessment of the participant's clinical condition, the investigator should consider referring the participant for a hepatologist or gastroenterologist consultation, magnetic resonance cholangiopancreatography (MRCP), endoscopic retrograde cholangiopancreatography (ERCP), cardiac echocardiogram, and/or a liver biopsy.

Additional Hepatic Safety Data Collection

Additional safety data (Table 5) should be collected via the CRF if one or more of the following conditions occur:

In participants enrolled with baseline ALT or AST <1.5×ULN

- Elevation of serum ALT or AST to ≥5×ULN on 2 or more consecutive blood tests
- The combination of elevated ALT or AST ≥3×ULN and elevated TBL ≥2×ULN

In participants enrolled with baseline ALT or AST ≥1.5×ULN

- Elevated ALT or AST ≥3× baseline on 2 or more consecutive tests
- The combination of elevated ALT or AST ≥2× baseline and elevated TBL ≥2×ULN

In all study participants

- Discontinuation from study intervention due to a hepatic event or abnormality of liver tests should be reported as an SAE

Table 3: Hepatic Monitoring Tests for Hepatic Treatment Emergent Abnormality

**Hematology**	**Clinical Chemistry**
Hemoglobin	Total bilirubin
Hematocrit	Direct bilirubin
Erythrocytes (RBCs - red blood cells)	Alkaline phosphatase (ALP)
Leukocytes (WBCs - white blood cells)	Alanine aminotransferase (ALT)
Differential:	Aspartate aminotransferase (AST)
Neutrophils, segmented	Gamma-glutamyl transferase (GGT)
Lymphocytes	Creatine kinase (CK)
Monocytes	Other Chemistry
Basophils	Acetaminophen
Eosinophils	Acetaminophen protein adducts
Platelets	Alkaline phosphatase isoenzymes
Cell morphology (RBC and WBC)	Ceruloplasmin
Coagulation	Copper
Prothrombin time, INR (PT-INR)	Ethyl alcohol (EtOH)
Serology	Haptoglobin
Hepatitis A virus (HAV) testing:	Immunoglobulin IgA (quantitative)
HAV total antibody	Immunoglobulin IgG (quantitative)
HAV IgM antibody	Immunoglobulin IgM (quantitative)
Hepatis B virus (HBV) testing:	Phosphatidylethanol (PEth)
Hepatitis B surface antigen (HBsAg)	Urine Chemistry
Hepatitis B surface antibody (anti-HBs)	Drug screen
Hepatitis B core total antibody (anti-HBc)	Ethyl glucuronide (EtG)
Hepatitis B core IgM antibody	Other Serology
Hepatitis B core IgG antibody	Anti-nuclear antibody (ANA)
HBV DNA [c]	Anti-smooth muscle antibody (ASMA) [a]
Hepatis C virus (HCV) testing:	Anti-actin antibody [b]
HCV antibody	Epstein-Barr virus (EBV) testing:
HCV RNA [c]	EBV antibody
Hepatitis D virus (HDV) testing:	EBV DNA [c]
HDV antibody	Cytomegalovirus (CMV) testing:
Hepatitis E virus (HEV) testing:	CMV antibody
HEV IgG antibody	CMV DNA [c]
HEV IgM antibody	Herpes simplex virus (HSV) testing:
HEV RNA [c]	HSV (Type 1 and 2) antibody
Microbiology	HSV (Type 1 and 2) DNA [c]
Culture:	Liver kidney microsomal type 1 (LKM-1) antibody
Blood	
Urine	

Abbreviations: ALT = alanine aminotransferase; AST = aspartate aminotransferase; CPK = creatine phosphokinase; GGT = gamma-glutamyl transferase; Ig = immunoglobulin; INR = international normalized ratio; RBC = red blood cells; WBC = white blood cells.
[a] Not required if anti-actin antibody is tested.
[b] Not required if anti-smooth muscle antibody (ASMA) is tested.
c Reflex/confirmation dependent on regulatory requirements, testing availability, or both.

Dose level reductions should be made in 50 mg increments. For example, if the starting dose of abemaciclib for a study is 150 mg q12 hours, dose reduction 1 would be 100 mg q12 hours, dose reduction 2 would be 50 mg q12 hours.

7.3 Renal Toxicity

Elevation of serum creatinine is observed with abemaciclib, and is due to a pharmacological inhibitory effect of abemaciclib on renal tubular transporters without affecting glomerular function. The rise in serum creatinine (mean increase, 0.2 mg/dL) occurs within the first 28-day cycle of abemaciclib, and remains elevated but stable throughout the treatment period, and were reversible upon treatment discontinuation. Alternative markers (such as BUN, cystatin C level, or cystatin C calculated GFR) which are not based on creatinine, may be considered to determine whether renal function is impaired.

7.4 Hematologic Toxicity

Hematologic toxicities including neutropenia, leukopenia, anemia, and thrombocytopenia have been observed in participants treated with abemaciclib, and causality has been established. Severe (Grade 3 and 4) neutropenia was observed in participants receiving abemaciclib. Accordingly, participants should be monitored closely for signs of infection, anemia, and bleeding within the first 4.5 days of therapy. Accordingly, participants will be monitored with complete blood counts prior to the start of abemaciclib therapy, day 6, day 7 from surgery, 30 days (+/- 7 days) after last dose of abemaciclib (to be obtained by home institution) and as clinically indicated.

During maintenance therapy, participants may experience myelosuppression with temozolomide administrations. Participants will be monitored with complete blood counts prior to the start of every cycle. Repeat CBC will be obtained on Day 22 (21 days after first dose) or within 48 hours of that day after temozolomide administration and weekly until the ANC is above 1.5 x 10^9/L and platelet count exceeds 100 x 10^9/L. Adjust abemaciclib dosing according to the following table (**Table 4**):

Table 4: Dose Modification and Management – Hematologic Toxicities

CTCAE Grade	Maintenance Therapy Dose Modifications
Grade 1 or 2	No dose modification is required.
Persistent or recurrent Grade 2 or Grade 3	Suspend dose until toxicity resolves to baseline or Grade 1. Resume at next lower dose.
Grade 3, recurrent, or Grade 4	Discontinue temozolomide and abemaciclib protocol directed therapy and monitor until toxicities resolve

CTCAE Grade	Maintenance Therapy Dose Modifications
Participant requires administration of a blood cell growth factor	Discontinue temozolomide and abemaciclib protocol directed therapy and monitor until toxicities resolve

CTCAE Grade	Temozolomide Dose Modifications
Grade 1 or 2	No dose modification is required.
Persistent or recurrent Grade 2 or Grade 3	Suspend temozolomide and monitor until toxicities resolve to at least Grade 1, restart temozolomide therapy at 150mg/m^2 or 100mg/m^2 if during cycle 1.
Grade 3, recurrent, or Grade 4	Discontinue temozolomide and abemaciclib protocol directed therapy and monitor until toxicities resolve
Participant requires administration of a blood cell growth factor	Discontinue temozolomide and abemaciclib protocol directed therapy and monitor until toxicities resolve

If the toxicity is likely or potentially related to abemaciclib, the appropriate dose reductions for abemaciclib must occur. If it is likely or potentially related to temozolomide, then appropriate dose reductions for temozolomide should be made. If it is not possible to attribute the toxicity to either drug, consider reducing both. Dose level reductions should be made in 50 mg increments. For example, if the starting dose of abemaciclib for a study is 150 mg q12 hours, dose reduction 1 would be 100 mg q12 hours, dose reduction 2 would be 50 mg q12 hours.

7.5 NONHEMATOLOGIC TOXICITIES

Table 5: Dose Modification and Management – Nonhematologic Toxicities Excluding Diarrhea, ALT/AST Increased, and ILD/Pneumonitis

CTCAE Grade	Abemaciclib Dose Modifications
Grade 1 or 2	No dose modification is required.
Persistent or recurrent Grade 2 toxicity that does not resolve with maximal supportive measures within 7 days to baseline or Grade 1	Suspend dose until toxicity resolves to baseline or Grade 1. Resume at next lower dose.
Grade 3 or 4	

7.6 INTERSTITIAL LUNG DISEASE/ PNEUMONITIS EVENTS

Interstitial lung disease (ILD) / pneumonitis has been identified as an adverse drug reaction for abemaciclib. Adverse events reported included events such as interstitial lung disease, pneumonitis, obliterative bronchiolitis, organizing pneumonia, pulmonary fibrosis. The majority of events were Grade 1 or Grade 2 with serious cases and fatal events reported.

Participants will be monitored for clinical symptoms or radiological changes indicative of ILD/pneumonitis within the first 4.5 days of therapy and will be instructed to report any new or worsening pulmonary symptoms. Symptoms may include hypoxia, cough, dyspnea, or interstitial

infiltrates on radiologic exams; and will be treated as per local clinical practice and/or guidelines (including corticosteroids as appropriate). Infectious, neoplastic, and other causes for such symptoms should be excluded by means of appropriate investigations. Investigations may include imaging such as high resolution computer tomography (HRCT), bronchioalveolar lavage (BAL), and biopsy as clinically indicated. Abemaciclib dosing will not be reduced during maintenance therapy for ILD/pneumonitis events. For persistent or recurrent Grade 2 toxicity, during maintenance therapy, that does not resolve with maximal supportive measures within 7 days to baseline or Grade 1, abemaciblib dosing will be suspended until toxicity resolves to baseline or Grade 1 and then resumed at next lower dose. The following table for abemaciclib dose modifications applies to both pre-op and maintenance therapy (**Table**):

Table 6: Dose Modifications and Management- Interstitial Lung Disease/Pneumonitis

CTCAE Grade	Abemaciclib Dose Modifications
Grade 1 or 2	No dose modification is required.
Persistent or recurrent Grade 2 toxicity that does not resolve with maximal supportive measures within 7 days to baseline or Grade 1	Pre-operative: Discontinue abemaciclib and remove from study.<br>Maintenance: Suspend until toxicity resolves to baseline or grade 1 and then resume at next lower dose.
Grade 3 or 4	Discontinue abemaciclib.

Dose level reductions should be made in 50 mg increments. For example, if the starting dose of abemaciclib for a study is 150 mg q12 hours, dose reduction 1 would be 100 mg q12 hours, dose reduction 2 would be 50 mg q12 hours.

7.7 Venous Thromboembolic Events

Venous thromboembolic events were reported in participants receiving abemaciclib plus fulvestrant or aromatase inhibitors in breast cancer studies. Monitor participants for signs and symptoms of deep vein thrombosis (DVT) and pulmonary embolism (PE) and treat as medically appropriate

7.8 Cost and Compensation

7.8.1 Costs

NIH does not bill health insurance companies or participants for any research or related clinical care that participants receive at the NIH Clinical Center. If some tests and procedures are performed outside the NIH Clinical Center, participants may have to pay for these costs.

7.8.2 Compensation

Participants will not be compensated on this study.

7.8.3 Reimbursement

The NCI will cover the cost of some expenses associated with protocol participation. Some of these costs may be paid directly by the NIH and some may be reimbursed to the participant/guardian as appropriate. The amount and form of these payments are determined by the NCI Travel and Lodging Reimbursement Policy.

8 CRITERIA FOR REMOVAL FROM PROTOCOL THERAPY AND OFF STUDY CRITERIA

Prior to removal from study, effort must be made to have all participants complete a safety visit approximately 30 days following the last dose of study therapy

8.1.1 Criteria for removal from protocol therapy

See Section **6.3**

8.1.2 Off-Study Criteria

Participants who meet the following criteria should be discontinued from the study:

- Inability to maintain eligibility post-enrollment and prior to start of therapy, in the opinion of the Principal Investigator (see Section **3.5**)
- Screen failure
- Participant requests to be withdrawn from study
- Death
- Study is discontinued
- Lost to follow-up

8.2 LOST TO FOLLOW-UP

A participant will be considered lost to follow-up if he or she fails to return for 4 consecutive scheduled visits and is unable to be contacted by the study site staff.

The following actions must be taken if a participant fails to return to the clinic for a required study visit:

- The site will attempt to contact the participant and reschedule the missed visit within 2 weeks and counsel the participant on the importance of maintaining the assigned visit schedule and ascertain if the participant wishes to and/or should continue in the study.
- Before a participant is deemed lost to follow-up, the investigator or designee will make every effort to regain contact with the participant (where possible, 3 telephone calls and, if necessary, a certified letter to the participant's last known mailing address or local equivalent methods). These contact attempts should be documented in the participant's medical record or study file.
- Should the participant continue to be unreachable, he or she will be considered to have withdrawn from the study with a primary reason of lost to follow-up.

9 PHARMACEUTICAL INFORMATION

A list of the adverse events and potential risks associated with the investigational agent administered in this study can be found in Section **11.1**

9.1 CTEP IND AGENT(S)

9.1.1 CTEP IND Agent: Abemaciclib (NSC 783671)

Chemical Name: N-(5-((4-ethylpiperazin-1-yl)methyl)pyridin-2-yl)-5-fluoro-4-(4-fluoro-1-isopropyl-2-methyl-1H-benzo[d]imidazol-6-yl)pyrimidin-2-amine

Other names: LY2835219, Verzenio®

CAS number: 1231929-97-7

Classification: Oral inhibitor of cyclin-dependent kinases 4 and 6 (CKD4/6)

Molecular Formula: $C_{27}H_{32}F_2N_8$ **M.W.:** 506.60

Description: Abemaciclib drug substance is practically white to yellow solid.

Approximate Solubility: Abemaciclib is soluble in water at 0.001 mg/mL and soluble in ethanol at 22.313 mg/mL.

Mode of Action: CDK4 and CDK6 complex with D-type cyclins to initiate the transition from G1 to S phase of the cell cycle by phosphorylation and inactivation of the retinoblastoma (Rb) tumor-suppressor protein. Alterations in this pathway occur frequently in human cancers and involve either the loss of CDK inhibitors by mutation or epigenetic silencing, mutation/overexpression of either CDK4/CDK6 or cyclin D, or inactivation of Rb. These alterations render cells less dependent on mitogenic signaling for proliferation and cancers that arise with these alterations are potentially sensitive to pharmacologic inhibition of CDK4 and CDK6. Abemaciclib prevents Rb phosphorylation, leading to suppression of tumor growth.

How Supplied: Lilly supplies and the Pharmaceutical Management Branch, CTEP, DCTD, NCI distributes abemaciclib as commercially-labeled blister packs containing a modified oval, immediate-release, film-coated 50 mg tablet. Each blister pack contains 14 tablets.

Each tablet contains 50 mg of abemaciclib and the following excipients: microcrystalline cellulose, lactose monohydrate, croscarmellose sodium, silicon dioxide, and sodium stearyl fumarate. The film-coated beige color mixture consists of polyvinyl alcohol, titanium dioxide, polyethylene glycol, talc, iron oxide yellow and iron oxide red.

Storage: Store at 20° C to 25° C (68° F to 77° F); excursions permitted to 15° C to 30° C (59° F to 86° F)

If a storage temperature excursion is identified, promptly return abemaciclib to room temperature and quarantine the supplies. Provide a detailed report of the excursion (including documentation of temperature monitoring and duration of the excursion) to PMBAfterHours@mail.nih.gov for determination of suitability.

Stability: Dispense intact blister packs. Each blister pack will contain a labeled expiration date.

Route and Method of Administration: Oral. Take abemaciclib with or without food. Instruct participants to swallow abemaciclib tablets whole and not to chew, crush, or split tablets before swallowing.

If the participant vomits or misses a dose of abemaciclib, instruct the participant to take the next dose at its scheduled time.

Potential Drug Interactions: In vitro and vivo studies show that abemaciclib is metabolized to several oxidative metabolites primarily by cytochrome CYP3A, with formation of active metabolites M2, M18 and M20 being further eliminated by metabolism and biliary excretion. Co-administration with a strong inducer, like rifampin, reduces abemaciclib activity by 77%. Avoid co-administering strong inducers in participants while taking abemaciclib. Strong CYP3A4 inhibitors significantly increase the AUC of abemaciclib and its metabolites. Avoid co-administering strong CYP3A4 inhibitors and use caution with co-administered moderate or weak CYP3A4 inhibitors. Refer to the protocol document for specific dosing instructions if participants cannot be switched to non-CYP3A4 inhibitors.

No clinically relevant effect on the PK of abemaciclib was observed when it was given in combination with fulvestrant, anastrozole, letrozole, tamoxifen, exemestane, everolimus, trastuzumab, pemetrexed, gemcitabine, LY3023414, galunisertib, ramucirumab, caffeine, dextromethorphan, midazolam, loperamide and warfarin.

Abemaciclib and its active metabolites are substrates for both P-gp and BCRP. In vivo data indicate that the likelihood of drug interactions is low when abemaciclib is co-administered with specific inhibitors or inducers of P-gp and BCRP. In vitro, abemaciclib is not a substrate of hepatic uptake transporters, OATP1B1, OATP1B3, and OCT1 making it unlikely that inhibitors or inducers of these transporters will affect abemaciclib exposure.

In vitro studies showed downregulation of messenger ribonucleic acid of CYP 1A2, 2B6, 2C8, 2C9, 2D6, and 3A by abemaciclib and its active metabolites over a concentration range of 0.05 to 10 μM. The clinical relevance of this down regulation was investigated which found no clinically meaningful effect of abemaciclib on the PK of CYP substrates. Abemaciclib can be safely co-administered with drugs which are substrates of CYP enzymes.

In vitro, abemaciclib and its active metabolites did not induce CYP 1A2, 2B6 or 3A at clinically relevant concentrations.

Abemaciclib and its active metabolites inhibit renal transporters OCT2, MATE1, and MATE2-K at clinically relevant concentrations, suggesting that abemaciclib may reduce the renal clearance of substrates of these transporters, such as metformin. The clinical implications of the change in metformin exposure is unknown.

Inhibition of these transporters by abemaciclib and its active metabolites may also reduce renal clearance of creatinine, resulting in a concomitant rise in serum creatinine levels due to inhibition of creatinine tubular secretion via OCT2, MATE1, and MATE2-K. The serum creatinine increases observed in abemaciclib clinical studies are likely due to the inhibition of creatinine tubular secretion via OCT2, MATE1, and MATE2-K, and may not be due to decline in renal function.

The results of in vitro studies show that abemaciclib inhibits hepatic uptake transporter, OCT1, and efflux transporters, P-gp and BCRP.

Avoid concomitant use of narrow-therapeutic substrates of P-gp, such as digoxin and narrow therapeutic substrates of OCT1 and BCRP. Abemaciclib and its active metabolites did not significantly inhibit OATP1B1, OATP1B3, OAT1 and OAT3 at clinically relevant concentrations making drug interactions with substrates of these transporters unlikely.

Participant Care Implications: Individuals should not breastfeed during treatment with abemaciclib. Females of reproductive potential should use highly effective contraception prior to the study, for the duration of study treatment, and for 3 weeks after the last dose abemaciclib.

Based on findings in animals, abemaciclib may impair fertility in males of reproductive potential. Advise males with female partners of reproductive potential to use condoms during treatment with abemaciclib.

9.1.2 Availability

Abemaciclib is an investigational agent supplied to investigators by the Division of Cancer Treatment and Diagnosis (DCTD), NCI.

Abemaciclib is provided to the NCI under a Collaborative Agreement between the Pharmaceutical Collaborator and the DCTD, NCI (see Section **15.5**).

9.1.3 Agent Ordering and Agent Accountability

NCI-supplied agents may be requested by eligible participating Investigators (or their authorized designee) at each participating institution. The CTEP-assigned protocol number must be used for ordering all CTEP-supplied investigational agents. The eligible participating investigators at each participating institution must be registered with CTEP, DCTD through an annual submission of FDA Form 1572 (Statement of Investigator), NCI Biosketch, Agent Shipment Form, and Financial Disclosure Form (FDF). If there are several participating investigators at one institution, CTEP-supplied investigational agents for the study should be ordered under the name of one lead participating investigator at that institution.

Starter supplies will not be provided. Participants must be registered prior to agent ordering. Sites may request expedited orders Monday-Thursday when they provide courier information.

Submit agent requests through the PMB Online Agent Order Processing (OAOP) application. Access to OAOP requires the establishment of a CTEP Identity and Access Management (IAM) account and the maintenance of an “active” account status, a “current” password, and active person registration status. For questions about drug orders, transfers, returns, or accountability, call or email PMB any time. Refer to the PMB’s website for specific policies and guidelines related to agent management.

9.1.3.1 Agent Inventory Records

The investigator, or a responsible party designated by the investigator, must maintain a careful record of the receipt, dispensing and final disposition of all agents received from the PMB using the appropriate NCI Investigational Agent (Drug) Accountability Record (DARF) available on the CTEP forms page. Store and maintain separate NCI Investigational Agent Accountability Records for each agent, strength, formulation and ordering investigator on this protocol.

9.1.4 Investigator Brochure Availability

The current versions of the IBs for the agents will be accessible to site investigators and research staff through the PMB OAOP application. Access to OAOP requires the establishment of a CTEP IAM account and the maintenance of an “active” account status, a “current” password and active person registration status. Questions about IB access may be directed to the PMB IB Coordinator via email.

9.1.5 Useful Links and Contacts

- CTEP Forms, Templates, Documents: http://ctep.cancer.gov/forms/
- NCI CTEP Investigator Registration: RCRHelpDesk@nih.gov
- PMB policies and guidelines: http://ctep.cancer.gov/branches/pmb/agent_management.htm
- PMB Online Agent Order Processing (OAOP) application: https://ctepcore.nci.nih.gov/OAOP
- CTEP Identity and Access Management (IAM) account: https://ctepcore.nci.nih.gov/iam/
- CTEP IAM account help: ctepreghelp@ctep.nci.nih.gov

- IB Coordinator: IBCoordinator@mail.nih.gov
- PMB email: PMBAfterHours@mail.nih.gov
- PMB phone and hours of service: (240) 276-6575 Monday through Friday between 8:30 am and 4:30 pm (ET)

9.2 COMMERCIAL AGENT

9.2.1 Temozolomide

9.2.1.1 Product Description

Temozolomide is commercially available. Refer to the FDA approved package insert for complete product information. Capsules are supplied in child-resistant sachets. Repackaging is not allowed and capsules must be dispensed in the original container. If exact quantity must be dispensed, then extra capsules should be removed, documented as waste and destroyed immediately.

9.2.1.2 Solution Preparation

Refer to the package insert for standard preparation instructions.

9.2.1.3 Route of Administration

See Section **6.1.2.**

There are no dietary restrictions with temozolomide. To reduce nausea and vomiting, temozolomide should be taken on an empty stomach. Bedtime administration may be advised. Antiemetic therapy may be administered prior to and/or following administration of temozolomide. Capsules should not be opened or chewed. They should be swallowed whole with a glass of water. If capsules are accidentally opened or damaged, precautions should be taken to avoid inhalation or contact with the skin or mucous membranes.

9.2.1.4 Agent Ordering

Temozolomide is commercially available and will be purchased by the NIH Clinical Center Pharmacy Department. Refer to the FDA approved package insert for complete product information.

10 STATISTICAL CONSIDERATIONS

10.1 STATISTICAL DESIGN/ENDPOINTS

10.1.1 Primary Endpoints

- To evaluate safety and feasibility of intratumoral microdialysis placement post high grade glioma resection or midline glioma biopsy
- To evaluate safety and feasibility of brain interstitial dialysate sampling in glioma participants post abemaciclib administration
- To measure intratumoral vs. systemic concentrations of abemaciclib in glioma participants post abemaciclib administration

10.1.2 Secondary Endpoints

- To determine the impact of results from abemaciclib PK and PD studies on subsequent treatment and participant outcomes

10.1.3 General Approach

The fraction of participants who experience any adverse event /complication will be reported, along with the grades and types of adverse events. The fraction of participants who have adequate intra-tumoral sampling will be reported

10.1.4 Analysis of the Primary Endpoints

The fraction of participants who experience any adverse event /complication will be reported, along with the grades and types of adverse events. The fraction of participants who have adequate intra-tumoral sampling will be reported, along with 80% and 95% confidence intervals.

Intratumoral vs. systemic concentrations of abemaciclib in high grade glioma resection or midline glioma participants post abemaciclib administration will be determined with results reported using descriptive statistics.

10.1.5 Analysis of the Secondary Endpoints

To determine the impact of results from PK and PD studies conducted on the trial on subsequent treatment and outcome, descriptive statistics may be reported.

10.1.6 Safety Analyses

All participants who receive at least one dose of abemaciclib or undergo the procedure to place the catheter will have their safety information reported, by type and grade of complication or adverse event.

10.1.7 Population for Analysis

Intention to treat: any participants who enroll onto the trial and provide consent and who receive a procedure to place the catheter will be included in safety analysis. If at least one intra-tumoral sample can be obtained, the participant will be included in efficacy analyses.

10.1.8 Exploratory Analyses

- Measure abemaciclib phosphorylated RB (pRB) and topoisomerase II alpha (TopoIIα, specific for S phase) pharmacodynamic assays to assess CDK4/6 inhibition and cell cycle progression in biopsied tumor tissue
- Measure dexamethasone concentrations in brain interstitial fluid to evaluate passive diffusion of known blood-brain barrier (BBB) permeable compound
- Measure urea concentrations in blood and brain interstitial fluid to evaluate in microdialysis catheter performance
- Conduct genomic sequencing of biopsied tumor tissue identifying driver mutations linked to targeted therapies
- Establish participant derived xenograft modeling in rodents using biopsied tumor tissue
- Evaluate median progression free survival with continued abemaciclib with temozolomide therapy
- Evaluate standard pharmacokinetic parameters comparing combined abemaciclib and temozolomide vs. abemaciclib alone vs. historical controls of temozolomide only

Any of these exploratory evaluations which generate quantitative measures will be done using descriptive statistics including confidence intervals when appropriate. Any statistical tests performed for evaluation of exploratory objectives will be done without formal adjustment for multiple comparisons, but in the context of the number of tests performed.

10.2 SAMPLE SIZE/ACCRUAL RATE

The primary aim of this study is to assess the safety and feasibility of dialysis catheter placement and use. This is intended to be a preliminary trial to investigate safety and feasibility. As such, this trial will initially be limited to 5 evaluable participants, with possible expansion by a later amendment. Initially 2 participants with recurrent cortical high grade glioma will be enrolled on study to assess safety and feasibility of microdialysis placement and evaluation with real-time pharmacokinetic measurements conducted. Then 3 participants with recurrent diffuse midline glioma will be enrolled on study.

For purposes of this preliminary evaluation, the intra-tumoral microdialysis placement will be considered feasible for a given participant if we are able to place the catheter and obtain analyzable dialysate samples in at least 7 but preferably 10 (80%) or more of the 13 intended time points to evaluate the concentration of agents (participants who are not evaluable by having only 6 or fewer analyzable samples, or for other reasons, will be replaced) with no participants experiencing serious complications with microdialysis insertion/maintenance (no Grade 3 or higher bleeding, infection or neurologic adverse events in any participant). The timing of the usable dialysate samples will also determine if the results are adequately spread out over the intended time interval to demonstrate a feasible outcome for that participant. If so, this will be considered sufficient for purposes of this preliminary, exploratory study. If the placement was done with no serious complications in any participant, and if adequate, usable samples are able to be obtained from the microdialysis catheter in at least 4 of the 5 participants, then further use of this device may be considered in this trial by an amendment, or in a subsequent study, either of which would include a formal power analysis to justify the number of participants to enroll on the basis of a defined primary endpoint.

Another primary aim is to measure intratumoral vs. systemic concentrations of abemaciclib in glioma participants post abemaciclib administration.

Exploratory aims of the study are to conduct genomic analysis from tissue and pharmacokinetic studies during maintenance therapy. Descriptive statistics will be obtained (no interventions yielding comparison of participant groups). As such, no formal power analysis will be performed related to the exploratory endpoints. Priority for tissue studies 1) pathology, 2) molecular profiling, 3) pharmacology, 4) PDX modeling. Given the small study population, clinical data will be presented as median with range and categorical variables presented as proportions (%). All PK and PD samples will be batched until further analysis.

It is expected that up to 1 year may be required to enroll up to 5 evaluable participants. To allow for a small number of inevaluable participants to be replaced, the accrual ceiling will be set at 7 participants.

**PLANNED ENROLLMENT REPORT**					
**Racial Categories**	**Ethnic Categories**				**Total**
	**Not Hispanic or Latino**		**Hispanic or Latino**		
	**Female**	**Male**	**Female**	**Male**	

PLANNED ENROLLMENT REPORT					
American Indian/ Alaska Native	0	0	0	0	0
Asian	0	0	0	0	0
Native Hawaiian or Other Pacific Islander	0	0	0	0	0
Black or African American	0	1	0	0	1
White	3	3	0	0	6
More Than One Race	0	0	0	0	0
**Total**	3	4	0	0	7

11 ADVERSE EVENTS: LIST AND REPORTING REQUIREMENTS

Adverse event (AE) monitoring and reporting is a routine part of every clinical trial. The following list of AEs (Section 11.1) and the characteristics of an observed AE will determine whether the event requires expedited reporting via the CTEP Adverse Event Reporting System (CTEP-AERS) **in addition** to routine reporting.

11.1 COMPREHENSIVE ADVERSE EVENTS AND POTENTIAL RISKS LISTS(S) (CAEPRS)

11.1.1 CAEPRs for CTEP IND Agent

11.1.1.1 CAEPR for Abemaciclib

Comprehensive Adverse Events and Potential Risks list (CAEPR)
for
Abemaciclib (LY2835219, NSC 783671)

The Comprehensive Adverse Events and Potential Risks list (CAEPR) provides a single list of reported and/or potential adverse events (AE) associated with an agent using a uniform presentation of events by body system. In addition to the comprehensive list, a subset, the Specific Protocol Exceptions to Expedited Reporting (SPEER), appears in a separate column and is identified with bold and italicized text. This subset of AEs (SPEER) is a list of events that are protocol specific exceptions to expedited reporting to NCI (except as noted below). Refer to the 'CTEP, NCI Guidelines: Adverse Event Reporting Requirements' http://ctep.cancer.gov/protocolDevelopment/electronic_applications/docs/aeguidelines.pdf for further clarification. *Frequency is provided based on 950 patients.* Below is the CAEPR for Abemaciclib (LY2835219).

> **NOTE**: Report AEs on the SPEER **ONLY IF** they exceed the grade noted in parentheses next to the AE in the SPEER. If this CAEPR is part of a combination protocol using multiple investigational agents and has an AE listed on different SPEERs, use the lower of the grades to determine if expedited reporting is required.

Version 2.0, October 3, 2019[1]

Adverse Events with Possible Relationship to Abemaciclib (LY2835219) (CTCAE 5.0 Term) [n= 950]			Specific Protocol Exceptions to Expedited Reporting (SPEER)
**Likely (>20%)**	**Less Likely (<=20%)**	**Rare but Serious (<3%)**	
BLOOD AND LYMPHATIC SYSTEM DISORDERS			
Anemia			***Anemia (Gr 2)***
GASTROINTESTINAL DISORDERS			
	Abdominal pain		
Diarrhea			***Diarrhea (Gr 2)***
	Dry mouth		
	Mucositis oral		
Nausea			***Nausea (Gr 2)***
Vomiting			***Vomiting (Gr 2)***
GENERAL DISORDERS AND ADMINISTRATION SITE CONDITIONS			
Fatigue			***Fatigue (Gr 2)***
INVESTIGATIONS			
		Alanine aminotransferase increased	
		Aspartate aminotransferase increased	
	Creatinine increased		
	Lymphocyte count decreased		
Neutrophil count decreased			***Neutrophil count decreased (Gr 2)***
Platelet count decreased			***Platelet count decreased (Gr 2)***
	White blood cell decreased		
METABOLISM AND NUTRITION DISORDERS			
Anorexia			***Anorexia (Gr 2)***
NERVOUS SYSTEM DISORDERS			
	Dysgeusia		
	Headache		
RESPIRATORY, THORACIC AND MEDIASTINAL DISORDERS			
		Pneumonitis	
SKIN AND SUBCUTANEOUS TISSUE DISORDERS			
	Alopecia		
VASCULAR DISORDERS			
		Thromboembolic event	

[1]This table will be updated as the toxicity profile of the agent is revised. Updates will be distributed to all Principal Investigators at the time of revision. The current version can be obtained by contacting PIO@CTEP.NCI.NIH.GOV. Your name, the name of the investigator, the protocol and the agent should be included in the e-mail.

Adverse events reported on abemaciclib (LY2835219) trials, but for which there is insufficient evidence to suggest that there was a reasonable possibility that abemaciclib (LY2835219) caused the adverse event:

EYE DISORDERS - Watering eyes
GASTROINTESTINAL DISORDERS - Constipation; Dyspepsia
GENERAL DISORDERS AND ADMINISTRATION SITE CONDITIONS - Edema limbs; Fever; Pain

INFECTIONS AND INFESTATIONS - Lung infection; Sepsis; Upper respiratory infection; Urinary tract infection
INVESTIGATIONS - Blood bilirubin increased; GGT increased; Weight loss
METABOLISM AND NUTRITION DISORDERS - Dehydration; Hypertriglyceridemia; Hypoalbuminemia; Hypokalemia; Hyponatremia
MUSCULOSKELETAL AND CONNECTIVE TISSUE DISORDERS - Arthralgia; Back pain; Bone pain; Generalized muscle weakness; Myalgia
NERVOUS SYSTEM DISORDERS - Somnolence
RENAL AND URINARY DISORDERS - Acute kidney injury
RESPIRATORY, THORACIC AND MEDIASTINAL DISORDERS - Cough; Dyspnea
SKIN AND SUBCUTANEOUS TISSUE DISORDERS - Pruritus
VASCULAR DISORDERS - Lymph leakage

Note: Abemaciclib (LY2835219) in combination with other agents could cause an exacerbation of any adverse event currently known to be caused by the other agent, or the combination may result in events never previously associated with either agent.

11.1.2 Adverse Event List for Commercial Agent

11.1.2.1 Temozolomide

The most common adverse reactions (≥10% incidence) are alopecia, fatigue, nausea, vomiting, headache, constipation, anorexia, convulsions, rash, hemiparesis, diarrhea, asthenia, fever, dizziness, coordination abnormal, viral infection, amnesia, and insomnia. The most common Grade 3 to 4 hematologic laboratory abnormalities (≥10% incidence) that have developed during treatment with temozolomide are: lymphopenia, thrombocytopenia, neutropenia, and leukopenia. Allergic reactions have also been reported. See package insert for full details.

12 STUDY CALENDAR

See **APPENDIX E** for a detailed schedule of assessments.

13 MEASUREMENT OF EFFECT

Disease response assessments will not be performed on this protocol.

13.1 Toxicity Criteria

The following adverse event management guidelines are intended to ensure the safety of each participant while on the study. The descriptions and grading scales found in the revised NCI Common Terminology Criteria for Adverse Events (CTCAE) version 5.0 will be utilized for AE reporting. All appropriate treatment areas should have access to a copy of the CTCAE version 5.0. A copy of the CTCAE version 5.0 can be downloaded from the CTEP web site (http://ctep.cancer.gov/protocolDevelopment/electronic_applications/ctc.htm).

14 NIH REPORTING REQUIREMENTS/DATA AND SAFETY MONITORING PLAN

14.1 Definitions

Please refer to definitions provided in Policy 801: Reporting Research Events found at: https://irbo.nih.gov/confluence/pages/viewpage.action?pageId=36241835#HRPPPolicies-800Series-ComplianceandResearchEventReportingRequirements.

14.2 OHSRP Office of Compliance and Training/ IRB Reporting

14.2.1 Expedited Reporting

Please refer to the reporting requirements in Policy 801: Reporting Research Events and Policy 802 Non-Compliance Human Subjects Research found at: https://irbo.nih.gov/confluence/pages/viewpage.action?pageId=36241835#HRPPPolicies-800Series-ComplianceandResearchEventReportingRequirements. Note: Only IND Safety Reports that meet the definition of an unanticipated problem will need to be reported per these policies.

14.2.2 IRB Requirements for PI Reporting at Continuing Review

Please refer to the reporting requirements in Policy 801: Reporting Research Events found at: https://irbo.nih.gov/confluence/pages/viewpage.action?pageId=36241835#HRPPPolicies-800Series-ComplianceandResearchEventReportingRequirements.

14.3 NCI Clinical Director Reporting

Problems expeditiously reviewed by the OHSRP/IRB in the NIH eIRB system will also be reported to the NCI Clinical Director/designee; therefore, a separate submission for these reports is not necessary.

In addition to those reports, all deaths that occur within 30 days after receiving a research intervention should be reported via email unless they are due to progressive disease.

To report these deaths, please send an email describing the circumstances of the death to NCICCRQA@mail.nih.gov within one business day of learning of the death.

15 STUDY OVERSIGHT AND DATA REPORTING/REGULATORY REQUIREMENTS

Adverse event lists, guidelines, and instructions for AE reporting can be found in Section **11**

15.1 Study Oversight

This protocol is monitored at several levels, as described elsewhere in this section. The Protocol Principal Investigator is responsible for monitoring the conduct and progress of the clinical trial, including the ongoing review of accrual, participant-specific clinical and laboratory data, and routine and serious adverse events; reporting of expedited adverse events; and accumulation of reported adverse events from other trials testing the same drug(s). The Protocol Principal Investigator and statistician have access to the data at all times.

All Study Investigators at participating sites who register/enroll participants on a given protocol are responsible for timely submission of data via the mechanism described elsewhere in this section. All studies are also reviewed in accordance with the enrolling institution's data safety monitoring plan.

15.2 Data Reporting

Medidata Rave is the clinical data management system being used for data collection for this trial/study. Access to the trial in Rave is controlled through the CTEP-IAM system and role assignments.

Requirements to access Rave via iMedidata:

- A valid CTEP-IAM account and linked ID.me account (ID.me accounts are required for all newly created CTEP-IAM accounts and by July 1, 2023 for all users); and
- Assigned a Rave role on the LPO or PO roster at the enrolling site of: Rave CRA, Rave Read Only, Rave CRA (LabAdmin), Rave SLA, or Rave Investigator.

  Rave role requirements:

  - Rave CRA or Rave CRA (Lab Admin) role, must have a minimum of an Associate Plus (AP) registration type,
  - Rave Investigator role, must be registered as a Non-Physician Investigator (NPIVR) or Investigator (IVR), and
  - Rave Read Only or Rave SLA role must have at a minimum an Associate (A) registration type.
- Refer to https://ctep.cancer.gov/investigatorResources/default.htm for registration types and documentation required.

This study has a Delegation of Tasks Log (DTL). Therefore, those requiring write access to Rave must also be assigned the appropriate Rave tasks on the DTL.Upon initial site registration approval for the study in the Regulatory application, all persons with Rave roles assigned on the appropriate roster will be sent a study invitation e-mail from iMedidata. To accept the invitation, site staff must log in to the Select Login (https://login.imedidata.com/selectlogin) using their CTEP-IAM username and password, and click on the *accept* link in the upper right-corner of the iMedidata page. Site staff will not be able to access the study in Rave until all required Medidata and study specific trainings are completed. Trainings will be in the form of electronic learnings (eLearnings) and can be accessed by clicking on the link in the upper right pane of the iMedidata screen. If an eLearning is required and has not yet been taken, the link to the eLearning will appear under the study name in iMedidata instead of the *Rave EDC* link; once the successful completion of the eLearning has been recorded, access to the study in Rave will be granted, and a *Rave EDC* link will display under the study name.

Site staff who have not previously activated their iMedidata/Rave account at the time of initial site registration approval for the study in the Regulatory application will receive a separate invitation from iMedidata to activate their account. Account activation instructions are located on the CTSU website in the Data Management section under the Rave resource materials (Medidata Account Activation and Study Invitation Acceptance). Additional information on iMedidata/Rave is available on the CTSU members' website in the Data Management > Rave section or by contacting the CTSU Help Desk at 1-888-823-5923 or by e-mail at ctsucontact@westat.com.

15.2.1 Method

This study will be monitored by the Clinical Trials Monitoring Service (CTMS). Data will be submitted to CTMS at least once every two weeks via Medidata Rave (or other modality if approved by CTEP). Information on CTMS reporting is available at http://www.theradex.com/clinicalTechnologies/?National-Cancer-Institute-NCI-11. On-site audits will be conducted three times annually (one annual site visit and two data audits). For CTMS monitored studies, after users have activated their accounts, please contact the Theradex

Help Desk at (609) 619-7862 or by email at CTMSSupport@theradex.com for additional support with Rave and completion of CRFs.

15.2.2 Responsibility for Data Submission

Data are to be submitted via Medidata Rave to CTMS on a real-time basis, but no less than once every 2 weeks. The timeliness of data submissions and timeliness in resolving data queries will be tracked by CTMS. Metrics for timeliness will be followed and assessed on a quarterly basis. For the purpose of Institutional Performance Monitoring, data will be considered delinquent if it is greater than 4 weeks past due.

Data from Medidata Rave and CTEP-AERS is reviewed by the CTMS on an ongoing basis as data is received. Queries will be issued by CTMS directly within Rave. The queries will appear on the Task Summary Tab within Rave for the CRA at the site to resolve. Monthly web-based reports are posted for review by the Drug Monitors in the IDB, CTEP. Onsite audits will be conducted by the CTMS to ensure compliance with regulatory requirements, GCP, and NCI policies and procedures with the overarching goal of ensuring the integrity of data generated from NCI-sponsored clinical trials, as described in the Program Guidelines, which may be found on the CTEP (http://ctep.cancer.gov/protocolDevelopment/electronic_applications/adverse_events.htm).

CTMS will utilize a core set of eCRFs that are Cancer Data Standards Registry and Repository (caDSR) compliant (http://cbiit.nci.nih.gov/ncip/biomedical-informatics-resources/interoperability-and-semantics/metadata-and-models). Customized eCRFs will be included when appropriate to meet unique study requirements. The PI is encouraged to review the eCRFs, working closely with CTMS to ensure prospectively that all required items are appropriately captured in the eCRFs prior to study activation. CTMS will prepare the eCRFs with built-in edit checks to the extent possible to promote data integrity.

CDUS data submissions for trials activated after March 1, 2014, will be carried out by the CTMS contractor, Theradex. CDUS submissions are performed by Theradex on a monthly basis. The trial's lead institution is responsible for timely submission to CTMS via Rave, as above.

Further information on data submission procedures can be found in the Program Guidelines (http://ctep.cancer.gov/protocolDevelopment/electronic_applications/adverse_events.htm).

15.3 DATA QUALITY PORTAL

The Data Quality Portal (DQP) provides a central location for site staff to manage unanswered queries and form delinquencies, monitor data quality and timeliness, generate reports, and review metrics.

The DQP is located on the CTSU members' website under Data Management. The Rave Home section displays a table providing summary counts of Total Delinquencies and Total Queries. DQP Queries, DQP Delinquent Forms, and the DQP Reports modules are available to access details and reports of unanswered queries, delinquent forms, and timeliness reports. Site staff should review the DQP modules on a regular basis to manage specified queries and delinquent forms.

The DQP is accessible by site staff who are rostered to a site and have access to the CTSU website. Staff who have Rave study access can access the Rave study data via direct links available in the DQP modules.

CTSU Delinquency Notification emails are sent to primary contacts at sites twice a month. These notifications serve as alerts that queries and/or delinquent forms require site review, providing a summary count of queries and delinquent forms for each Rave study that a site is participating in. Additional site staff can subscribe and unsubscribe to these notifications using the CTSU Report and Information Subscription Portal on the CTSU members' website.

To learn more about DQP use and access, click on the Help Topics button displayed on the Rave Home, DQP Queries, and DQP Delinquent Forms modules.

Note: Some Rave protocols may not have delinquent form details or reports specified on the DQP. A protocol must have the Calendar functionality implemented in Rave by the Lead Protocol Organization (LPO) for delinquent form details and reports to be available on the DQP. Site staff should contact the LPO Data Manager for their protocol regarding questions about Rave Calendaring functionality.

15.4 NIH Required Data and Safety Monitoring Plan

15.4.1 Principal Investigator/Research Team

The clinical research team will meet on a regular basis (approximately weekly) when participants are being actively treated on the trial to discuss each participant. Decisions about dose level enrollment and dose escalation if applicable will be made based on the toxicity data from prior participants. Adverse events, deviations, response evaluations, and all other study related issues will be reviewed at this time.

All data will be collected in a timely manner and reviewed by the principal investigator or a lead associate investigator. Events meeting the requirements for expedited reporting as described in Section **14.2.1** will be submitted within the appropriate timelines.

The principal investigator will review adverse event and response data on each participant to ensure safety and data accuracy. The principal investigator will personally conduct or supervise the investigation and provide appropriate delegation of responsibilities to other members of the research staff.

15.5 Collaborative Agreements Language

The agent(s) supplied by CTEP, DCTD, NCI used in this protocol is/are provided to the NCI under a Collaborative Agreement (CRADA) between the Pharmaceutical Company(ies) (hereinafter referred to as "Collaborator(s)") and the NCI Division of Cancer Treatment and Diagnosis. Therefore, the following obligations/guidelines, in addition to the provisions in the "Intellectual Property Option to Collaborator" (http://ctep.cancer.gov/industryCollaborations2/intellectual_property.htm) contained within the terms of award, apply to the use of the Agent(s) in this study:

1. Abemaciclib may not be used for any purpose outside the scope of this protocol, nor can abemaciclib be transferred or licensed to any party not participating in the clinical study. Collaborator data for abemaciclib are confidential and proprietary to Collaborator and shall be maintained as such by the investigators. The protocol documents for studies utilizing Agents contain confidential information and should not be shared or distributed without the permission of the NCI. If a copy of this protocol is requested by a participant or participant's family member participating on the study, the individual should sign a confidentiality agreement. A suitable model agreement can be downloaded from: http://ctep.cancer.gov.

2. For a clinical protocol where there is an investigational Agent used in combination with (an)other Agent(s), each the subject of different Collaborative Agreements, the access to and use of data by each Collaborator shall be as follows (data pertaining to such combination use shall hereinafter be referred to as "Multi-Party Data”):

    a. NCI will provide all Collaborators with prior written notice regarding the existence and nature of any agreements governing their collaboration with NCI, the design of the proposed combination protocol, and the existence of any obligations that would tend to restrict NCI's participation in the proposed combination protocol.

    b. Each Collaborator shall agree to permit use of the Multi-Party Data from the clinical trial by any other Collaborator solely to the extent necessary to allow said other Collaborator to develop, obtain regulatory approval or commercialize its own Agent.

    c. Any Collaborator having the right to use the Multi-Party Data from these trials must agree in writing prior to the commencement of the trials that it will use the Multi-Party Data solely for development, regulatory approval, and commercialization of its own Agent.

3. Clinical Trial Data and Results and Raw Data developed under a Collaborative Agreement will be made available to Collaborator(s), the NCI, and the FDA, as appropriate and unless additional disclosure is required by law or court order as described in the IP Option to Collaborator (http://ctep.cancer.gov/industryCollaborations2/intellectual_property.htm). Additionally, all Clinical Data and Results and Raw Data will be collected, used and disclosed consistent with all applicable federal statutes and regulations for the protection of human subjects, including, if applicable, the *Standards for Privacy of Individually Identifiable Health Information* set forth in 45 C.F.R. Part 164.

4. When a Collaborator wishes to initiate a data request, the request should first be sent to the NCI, who will then notify the appropriate investigators (Group Chair for Cooperative Group studies, or PI for other studies) of Collaborator's wish to contact them.

5. Any data provided to Collaborator(s) for Phase 3 studies must be in accordance with the guidelines and policies of the responsible Data Monitoring Committee (DMC), if there is a DMC for this clinical trial.

6. Any manuscripts reporting the results of this clinical trial must be provided to CTEP by the Group office for Cooperative Group studies or by the principal investigator for non-Cooperative Group studies for immediate delivery to Collaborator(s) for advisory review and comment prior to submission for publication. Collaborator(s) will have 30 days from the date of receipt for review. Collaborator shall have the right to request that publication be delayed for up to an additional 30 days in order to ensure that Collaborator’s confidential and proprietary data, in addition to Collaborator(s)’s intellectual property rights, are protected. Copies of abstracts must be provided to CTEP for forwarding to Collaborator(s) for courtesy review as soon as possible and preferably at least three (3) days prior to submission, but in any case, prior to presentation at the meeting or publication in the proceedings. Press releases and

other media presentations must also be forwarded to CTEP prior to release. Copies of any manuscript, abstract and/or press release/ media presentation should be sent to:

Email: ncicteppubs@mail.nih.gov

The Regulatory Affairs Branch will then distribute them to Collaborator(s). No publication, manuscript or other form of public disclosure shall contain any of Collaborator's confidential/ proprietary information.

16 DATA COLLECTION AND EVALUATION

16.1 DATA COLLECTION

The PI will be responsible for overseeing entry of data into a password protected electronic system and ensuring data accuracy, consistency and timeliness. The principal investigator, associate investigators/research nurses and/or a contracted data manager will assist with the data management efforts. All data obtained during the conduct of the protocol will be kept in secure network drives or in approved alternative sites that comply with NIH security standards. Primary and final analyzed data will have identifiers so that research data can be attributed to an individual human subject participant.

All adverse events, including clinically significant abnormal findings on laboratory evaluations, regardless of severity, will be followed until return to baseline or stabilization of event. Document AEs from the first study intervention, Study Day 1 through 30 days after the last intervention. Beyond 30 days after the last intervention, only adverse events which are serious and related to the study intervention need to be recorded.

Additional information:

- Case Report Forms: Data entry will be completed within 2 weeks of each required evaluation. Protocol required appendices will be completed within 2 weeks of each required evaluation.
- Pharmacokinetic studies: Blood samples and corresponding PK worksheet will be sent to the Figg lab (see Section **5.6.1**).

End of study procedures: Data will be stored according to HHS, FDA regulations and NIH Intramural Records Retention Schedule as applicable.

Loss or destruction of data: Should we become aware that a major breach in our plan to protect participant confidentiality and trial data has occurred, this will be reported expeditiously per requirements in Section **14.2.1**.

16.1.1 Data Collection and Recording Exceptions

16.1.1.1 Abnormal Laboratory Values

An abnormal laboratory value will be recorded in the database as an AE **only** if the laboratory abnormality is characterized by any of the following:

- Results in discontinuation from the study
- Is associated with clinical signs or symptoms
- Requires treatment or any other therapeutic intervention
- Is associated with death or another serious adverse event, including hospitalization.

- Is judged by the Investigator to be of significant clinical impact
- If any abnormal laboratory result is considered clinically significant, the investigator will provide details about the action taken with respect to the test drug and about the participant's outcome.

16.2 Genomic Data Sharing Plan

Unlinked genomic data will be deposited in public genomic databases such as dbGaP in compliance with the NIH Genomic Data Sharing Policy.

16.3 Incidental/Secondary Findings Disclosure Procedure

Participants will be contacted if a clinically actionable gene variant is discovered. Clinically actionable findings for this study are defined as disorders appearing in the American College of Medical Genetics and Genomics recommendations for the return of incidental findings that is current at the time of primary analysis. (A list of current guidelines is maintained on the CCR intranet: https://ccrod.cancer.gov/confluence/display/CCRCRO/Incidental+Findings+Lists).

If the research findings are verified in the CLIA certified lab, the participant will be offered the opportunity to come to NIH to have genetic education and counseling to explain this result; at the time of any such event(s), these activities will be funded by the NCI/CCR in consideration of the specific circumstances. If the participant does not want to come to NIH, a referral to a local genetic healthcare provider will be provided (at their expense).

This is the only time during the course of the study that incidental findings will be returned. No interrogations regarding clinically actionable findings will be made after the primary analysis.

16.4 Adverse Event Characteristics

- **CTCAE term (AE description) and grade:** The descriptions and grading scales found in the revised NCI Common Terminology Criteria for Adverse Events (CTCAE) version 5.0 will be utilized for AE reporting. All appropriate treatment areas should have access to a copy of the CTCAE version 5.0. A copy of the CTCAE version 5.0 can be downloaded from the CTEP website http://ctep.cancer.gov/protocolDevelopment/electronic_applications/ctc.htm.
- **For expedited reporting purposes only:**
    - AEs for the agent that are ***bold and italicized*** in the CAEPR (*i.e.*, those listed in the SPEER column, Section **11.1.1.1**) should be reported through CTEP-AERS only if the grade is above the grade provided in the SPEER.
- **Attribution** of the AE:
    - Definite – The AE *is clearly related* to the study treatment.
    - Probable – The AE *is likely related* to the study treatment.
    - Possible – The AE *may be related* to the study treatment.
    - Unlikely – The AE *is doubtfully related* to the study treatment.
    - Unrelated – The AE *is clearly NOT related* to the study treatment.

16.5 EXPEDITED ADVERSE EVENT REPORTING

16.5.1 Rave-CTEP-AERS Integration

The Rave Cancer Therapy Evaluation Program Adverse Event Reporting System (CTEP-AERS) integration enables evaluation of post-baseline AEs entered in Rave to determine whether they require expedited reporting and facilitates entry in CTEP-AERS for those AEs requiring expedited reporting.

All AEs that occur after baseline are collected in Medidata Rave using the Adverse Event form, which is available for entry at each treatment or reporting period and is used to collect AEs that start during the period or persist from the previous reporting period. CRA will enter AEs that occur prior to the start of treatment on a baseline form that is not included in the Rave-CTEP-AERS integration. AEs that occur prior to enrollment must begin and end on the baseline Adverse Event form and should not be included on the standard Adverse Events form that is available at treatment unless there has been an increase in grade.

Prior to sending AEs through the rules evaluation process, site staff should verify the following on the Adverse Event form in Rave:

- The reporting period (course/cycle) is correct, and
- AEs are recorded and complete (no missing fields) and the form is query-free.

The CRA reports AEs in Rave at the time the Investigator learns of the event. If the CRA modifies an AE, it must be re-submitted for rules evaluation.

Upon completion of AE entry in Medidata Rave, the CRA submits the AE for rules evaluation by completing the Expedited Reporting Evaluation form (i.e., checking the box Send All AEs for Evaluation and save the form). Both NCI and protocol-specific reporting rules evaluate the AEs submitted for expedited reporting. A report is initiated in CTEP-AERS using information entered in Medidata Rave for AEs that meet reporting requirements. The CRA completes the report by accessing CTEP-AERS via a direct link on the Medidata Rave Expedited Reporting Evaluation form.

In the rare occurrence that Internet connectivity is lost, a 24-hour notification is to be made to CTEP by telephone at 301-897-7497. Once internet connectivity is restored, the 24-hour notification that was phoned in must be entered immediately into CTEP-AERS using the direct link from Medidata Rave.

Additional information about the CTEP-AERS integration is available on the CTSU website:

- Study specific documents: Protocols > Documents > Education and Promotion, and
- Expedited Safety Reporting Rules Evaluation user guide: Resources > CTSU Operations Information > User Guides & Help Topics.

NCI requirements for SAE reporting are available on the CTEP website:

- NCI Guidelines for Investigators: Adverse Event Reporting Requirements is available at https://ctep.cancer.gov/protocolDevelopment/electronic_applications/docs/aeguidelines.pdf.

16.5.2 Distribution of Adverse Event Reports

CTEP-AERS is programmed for automatic electronic distribution of reports to the following individuals: Principal Investigator and Adverse Event Coordinator(s) (if applicable) of the Corresponding Organization or Lead Organization, the local treating physician, and the Reporter and Submitter. CTEP-AERS provides a copy feature for other e-mail recipients.

16.5.3 Expedited Reporting Guidelines

Use the NCI protocol number and the protocol-specific participant ID assigned during trial registration on all reports.

Note: A death on study requires <u>both</u> routine and expedited reporting, regardless of causality as long as the death occurred within 30 days after the last administration of the investigational agent. Attribution to treatment or other cause must be provided.

Death due to progressive disease should be reported as **Grade 5 "Disease progression"** in the system organ class (SOC) "General disorders and administration site conditions." Evidence that the death was a manifestation of underlying disease (*e.g.*, radiological changes suggesting tumor growth or progression: clinical deterioration associated with a disease process) should be submitted.

Phase 1 and Early Phase 2 Studies: Expedited Reporting Requirements for Adverse Events that Occur on Studies under an IND/IDE within 30 Days of the Last Administration of the Investigational Agent/Intervention [1, 2]

FDA REPORTING REQUIREMENTS FOR SERIOUS ADVERSE EVENTS (21 CFR Part 312)

NOTE: Investigators **<u>MUST</u>** immediately report to the sponsor (NCI) **<u>ANY</u>** Serious Adverse Events, whether or not they are considered related to the investigational agent(s)/intervention (21 CFR 312.64)

An adverse event is considered serious if it results in **<u>ANY</u>** of the following outcomes:

1) Death
2) A life-threatening adverse event
3) An adverse event that results in inpatient hospitalization or prolongation of existing hospitalization for ≥ 24 hours
4) A persistent or significant incapacity or substantial disruption of the ability to conduct normal life functions
5) A congenital anomaly/birth defect.
6) Important Medical Events (IME) that may not result in death, be life threatening, or require hospitalization may be considered serious when, based upon medical judgment, they may jeopardize the patient or subject and may require medical or surgical intervention to prevent one of the outcomes listed in this definition. (FDA, 21 CFR 312.32; ICH E2A and ICH E6).

<u>ALL</u> <u>SERIOUS</u> adverse events that meet the above criteria MUST be immediately reported to the NCI via electronic submission within the timeframes detailed in the table below.

Hospitalization	Grade 1 and Grade 2 Timeframes	Grade 3-5 Timeframes
Resulting in Hospitalization ≥ 24 hrs	10 Calendar Days	24-Hour 5 Calendar Days
Not resulting in Hospitalization ≥ 24 hrs	Not required	

**NOTE:** Protocol specific exceptions to expedited reporting of serious adverse events are found in the Specific Protocol Exceptions to Expedited Reporting (SPEER) portion of the CAEPR.<br><br>**Expedited AE reporting timelines are defined as:**<br>o "24-Hour; 5 Calendar Days" - The AE must initially be submitted electronically within 24 hours of learning of the AE, followed by a complete expedited report within 5 calendar days of the initial 24-hour report.<br>o "10 Calendar Days" - A complete expedited report on the AE must be submitted electronically within 10 calendar days of learning of the AE.
[1]Serious adverse events that occur more than 30 days after the last administration of investigational agent/intervention and have an attribution of possible, probable, or definite require reporting as follows:<br>**Expedited 24-hour notification followed by complete report within 5 calendar days for:**<br>• All Grade 3, 4, and Grade 5 AEs<br>**Expedited 10 calendar day reports for:**<br>• Grade 2 AEs resulting in hospitalization or prolongation of hospitalization<br><br>[2]For studies using PET or SPECT IND agents, the AE reporting period is limited to 10 radioactive half-lives, rounded UP to the nearest whole day, after the agent/intervention was last administered. Footnote "1" above applies after this reporting period.<br><br>Effective Date: May 5, 2011

16.6 Routine Adverse Reporting

All Adverse Events **must** be reported in routine study data submissions. **AEs reported expeditiously through CTEP-AERS must also be reported in routine study data submissions.**

Adverse event data collection and reporting, which are required as part of every clinical trial, are done to ensure the safety of participants enrolled in the studies as well as those who will enroll in future studies using similar agents. AEs are reported in a routine manner at scheduled times during the trial using Medidata Rave. For this trial the Adverse Event CRF is used for routine AE reporting in Rave.

16.7 Pregnancy

Although not an adverse event in and of itself, pregnancy as well as its outcome must be documented via **CTEP-AERS**. In addition, the ***Pregnancy Information Form*** included within the NCI Guidelines for Adverse Event Reporting Requirements must be completed and submitted to CTEP. Any pregnancy occurring in a participant or participant's partner from the time of consent to 90 days after the last dose of study drug must be reported and then followed for outcome. Newborn infants should be followed until 30 days old. Please see the "NCI Guidelines for Investigators: Adverse Event Reporting Requirements for DCTD (CTEP and CIP) and DCP INDs and IDEs" (at http://ctep.cancer.gov/protocolDevelopment/adverse_effects.htm) for more details on how to report pregnancy and its outcome to CTEP.

Pregnant partners may be offered the opportunity to participate in an institutional pregnancy registry protocol (e.g., the NIH IRP pregnancy registry study) to provide data about the outcome of the pregnancy for safety reporting purposes.

16.8 Secondary Malignancy

A *secondary malignancy* is a cancer caused by treatment for a previous malignancy (*e.g.*, treatment with investigational agent/intervention, radiation or chemotherapy). A secondary malignancy is not considered a metastasis of the initial neoplasm.

CTEP requires all secondary malignancies that occur following treatment with an agent under an NCI IND/IDE be reported expeditiously via CTEP-AERS. Three options are available to describe the event:

- Leukemia secondary to oncology chemotherapy (*e.g.*, acute myelocytic leukemia [AML])
- Myelodysplastic syndrome (MDS)
- Treatment-related secondary malignancy

Any malignancy possibly related to cancer treatment (including AML/MDS) should also be reported via the routine reporting mechanisms outlined in each protocol.

Please ensure that secondary malignancies are reported within 30 days with the pathology report.

16.9 Second Malignancy

A second malignancy is one unrelated to the treatment of a prior malignancy (and is **NOT** a metastasis from the initial malignancy). Second malignancies require **ONLY** routine AE reporting unless otherwise specified.

17 HUMAN SUBJECTS PROTECTIONS

17.1 Rationale For Participant Selection

Diffuse midline gliomas are the most aggressive brain tumors of child and adulthood. No groups, in regards to gender, and racial and ethnic groups, are being excluded from participation in the trial. Females who are pregnant or breastfeeding will not be eligible for the trial due to risks of fetal and teratogenic adverse events as seen animal studies.

17.2 Participation of Children

All participants enrolled on study will be ≥ 18 years old. The use of intracerebral microdialysis in children with brain tumors has never been performed or established as safe.

17.3 Participation of Participants Unable to Give Consent

Adults unable to consent may enroll in or be retained on this protocol because it is not uncommon for glioma patients to have have cognitive impairment to the degree that they lack capacity to give consent as a result of this neurological condition. Excluding these individuals from participation would significantly hinder the ability to recruit participants from the target patient population and meet study endpoints. All subjects ≥ age 18 who have capacity will be offered the opportunity to fill in their wishes for research and care, and assign a substitute decision maker on the "NIH Advance Directive for Health Care and Medical Research Participation" form so that another person can make decisions about their medical care in the event that they become incapacitated or cognitively impaired during the course of the study.

Note: The PI or AI will contact the NIH Ability to Consent Assessment Team (ACAT) for evaluation to assess ongoing capacity of the subjects and to identify an LAR, as needed.

Please see section **17.5.1** for consent procedure.

17.4 RISK/BENEFITS ASSESSMENT

17.4.1 Known Potential Risks

The primary risks to participants participating in this research study is from surgical biopsy, catheter placement, and toxicity of abemaciclib.

Risk of Biopsy:
Risks associated with cortical/midline biopsy include: bleeding, brain swelling, seizures, stroke, infection, blood clots, and reactions to anesthesia. Routine stereotactic needle biopsy will be performed, using neuronavigation. Over the past 5 years, surgical biopsies of midline gliomas were shown to be performed safely with acceptable risks.(53, 54) Additionally a recent study by Mueller et al, through the Pediatric Neuro-Oncology Consortium (PNOC003), examined whole exome sequencing and RNA sequencing in newly diagnosed DIPG participants to incorporate personalized treatments.(50) In their study of 17 DIPG participants receiving pontine biopsies, nine adverse events were reported to be related to surgery. Of these eight were grade 1 and one participant had worsening of his baseline grade 2 nystagmus to grade 3, that recovered back to baseline within 2 days from the biopsy.

Risk of Urine Collection:
There are no physical risks associated with urine collection.

Risk of EKG:
Side effects of EKG are skin irritation where EKG electrodes are placed.

Risks of Blood Sampling:
Side effects of blood draws include pain and bruising, lightheadedness, and rarely, fainting.

Risk of Microdialysis Catheter Placement:
Risks of microdialysis will be discussed with the participants, but the research literature indicates this procedure does not present more than minimal additional theoretical risk. Immediate theoretical risk of the surgery for placement would include bleeding, seizure, tissue scarring or infection. A recent study by Badie et al., with microdialysis catheters being implanted in the peritumoral area in brain tumor patients after resection, reported no morbidity from microdialysis catheter placement or sample collection.(55) A study of the use of the CMA 70 catheter in 174 patients revealed no incidents of hemorrhage or infection attributable to the microdialysis catheters. A study of microdialysis in recurrent glioblastoma patients prior to resection of their lesion also showed that in the 8 patients that had catheters implanted, there were no observed complications.(56) A recent article, using the MD 71 catheter, to investigate the cytokine changes in glioblastoma tissue and surrounding brain to radiotherapy.(57) In this study, 11 patients had biopsies, and then microdialysis catheters were placed directly into the tumor, and another catheter was placed 10mm away from the contrast-enhancing tissue. No morbidity was noted from placement of these catheters. In another study, 7 patients with malignant glioma or lymphoma had resections of their tumor, and then two MD 71 catheters placed – one at the tumor resection margin, and one 20mm away from the resection cavity.(58) No morbidity from placement of these catheters was noted in this study. The stereotactic biopsy is being done as a part of the biopsy and microdialysis implantation surgery, and it carries a risk of symptomatic hemorrhage of 1%.(59) Stereotactic biopsy with a frameless neuronavigation system carries a risk of about 1% of inaccurate biopsy.(60)

Risk of Abemaciclib:

The most common adverse reactions to abemaciclib (≥ 20%) in clinical studies were diarrhea, neutropenia, fatigue, infections, nausea, abdominal pain, anemia, vomiting, alopecia, decreased appetite and leukopenia. The protocol provides for detailed and careful monitoring of all participants to assess for toxicity and the dose escalation scheme is very conservative. Toxicity data from the current dose level will be collected and reviewed to ensure that there were no severe (dose-limiting) toxicities. Furthermore, by just entering the study, participants will be followed very carefully, in an organized coherent manner, which may also benefit their overall healthcare.

Risk of Temozolomide:

See Section **11.1.2.1**

Risk of anesthesia:
The duration of anesthesia for the research surgical procedure is expected to be approximately 120 minutes. The risks of anesthesia depend on the participant's age and concurrent medical conditions. More common side effects are temporary drowsiness, nausea, and vomiting following recovery from anesthesia. Rare side effects include aspiration, extreme changes in body temperature, hypertension or hypotension, changes in heart rate or rhythm, hypoxia, and allergic reactions to the anesthetic drugs. Some of these side effects may be life-threatening or lead to life-threatening conditions such as myocardial infarction or stroke. An anesthesiologist will evaluate participants for potential anesthesia risks prior to the procedure. To reduce the risk of aspiration, participants will not be allowed to eat or drink for 8 hours before the procedure, and until adequate recovery from anesthesia after the procedure. Some participants may also have an endotracheal tube inserted to reduce this risk and facilitate mechanical ventilation. Intubation is associated with a small risk of laryngospasm, bronchospasm, hypertension, damage to teeth and lips, swelling of the larynx, sore throat, and hoarseness due to injury or irritation of the larynx.

Risk of Microdialysis Catheter Sampling:
There is minimal risk to microdialysis sample retrieval. Microdialysis catheters have been used in clinical patient care for periods of time up to 10 days without increased risk of infection.(61) A recently completed study with the CMA 70 catheter in 12 patients with recurrent glioblastoma patients.(62) These patients received either a biopsy or a surgical resection, and then microdialysis catheters were placed into the wall of the resection cavity or tumor tissue. Levels of 5-FU, the expected metabolite of the initial drug, 5-FC, were measured by microdialysis for a minimum of 8 days, and these measurements continued up to 11 days. No infections (or any other morbidity) were reported in this trial. Given the evidence, we do not believe that there is an increased risk of infection for microdialysis sampling for 2 days.

Risk of Gadolinium-Enhanced Magnetic Resonance Imaging (MRI) and Computed tomography (CT) without contrast:
Study procedures including MRI/CT scans which require prolonged supine positioning, frequent blood draws which can cause bruising or bleeding at the site of the IV may cause participant discomfort. People with fear of confined spaces may become anxious during an MRI. The noise from the scanner is loud enough to damage hearing, especially in people who already have hearing loss. Symptoms from the gadolinium contrast infusion are usually mild and may include feeling hot, burning, or coldness in the arm during the injection, a metallic taste, headache, allergic reactions and nausea. Rarely, more severe symptoms have been reported including shortness of breath, wheezing, hives, and hypotension. People with kidney disease who receive gadolinium contrast are at risk for nephrogenic systemic fibrosis (NSF), a serious reaction that involves the

skin and can also involve the muscles, joints and internal organs and has resulted in a very small number of deaths.

Radiation Risk from Imaging:
The study will involve radiation from the following sources:

- Up to 1 CT (brain) scan
- Up to 1 chest X-ray

Participants in this study may be exposed to approximately 0.11 rem. This amount of radiation is less than the average yearly background radiation in the United States.

17.4.1.1 Non-Physical Risks of Genetic Research

Risk of receiving unwanted information:
Anxiety and stress may arise as a result of the anticipation that unwanted information regarding disease related DNA sequencing or disease tendencies, or misattributed paternity. Participants will be clearly informed that the data related to DNA sequencing and genetic analysis is coded, investigational and will not be shared with participants, family members or health care providers.

Risk related to possibility that information may be released:
This includes the risk that data related to genotype, DNA sequencing or risk for disease tendency or trait can be released to members of the public, insurers, employers, or law enforcement agencies. Although there are no plans to release results to the participants, family members or health care providers, this risk will be included in the informed consent document.

Risk to family or relatives:
Family members or relatives may or may not want to be aware of familial tendencies or genetic risks of disease which may cause anxiety about possible future health problems. As previously noted, participants will be notified of any medically significant and actionable incidental findings. Study results will not be shared with participants.

17.4.2 Known Potential Benefits
Previous studies evaluating overall response rates post treatment in brain stem gliomas have demonstrated some efficacy in inhibition of the CDK pathway. Abemaciclib (Verzenio®) is a CDK4/6 inhibitor drug that is FDA approved for advanced or metastatic breast cancer. Previous studies using glioblastoma xenograft models treated demonstrated the ability of abemaciclib to cross the blood-brain barrier, increase survival, and decrease tumor growth when given as a single agent or in combination with temozolomide. The potential benefits from this therapy are identification of targetable genetic alterations which can be matched with potential therapeutics for maintenance therapy post radiation. These therapies have the potential to stabilize or reduce tumor burden post radiation.

17.4.3 Assessment of Potential Risks and Benefits
There are risks associated with abemaciclib administration and surgical biopsy/resection and microdialysis placement, however we believe the potential benefits of treating targetable tumor PK and PD findings outweigh the risks. Adults who are unable to consent are included in this protocol because the protocol offers a prospect of direct benefit.

17.5 CONSENT PROCESS AND DOCUMENTATION

The informed consent document will be provided as a physical or electronic document to the participant or consent designee(s) as applicable for review prior to consenting. A designated study investigator will carefully explain the procedures and tests involved in this study, and the associated risks, discomforts and benefits. In order to minimize potential coercion, as much time as is needed to review the document will be given, including an opportunity to discuss it with friends, family members and/or other advisors, and to ask questions of any designated study investigator. A signed informed consent document will be obtained prior to entry onto the study.

The initial consent process as well as re-consent, when required, may take place in person or remotely (e.g., via telephone or other NIH approved remote platforms used in compliance with policy, including HRPP Policy 303) per discretion of the designated study investigator and with the agreement of the participant/consent designee(s). Whether in person or remote, the privacy of the participant will be maintained. Consenting investigators (and participant/consent designee, when in person) will be located in a private area (e.g., clinic consult room). When consent is conducted remotely, the participant/consent designee will be informed of the private nature of the discussion and will be encouraged to relocate to a more private setting if needed.

Note: When required, witness signature will be obtained similarly as described for the investigator and participant as described below.

Consent will be documented with required signatures on the physical document (which includes the printout of an electronic document sent to participant) or as described below, with a manual (non-electronic) signature on the electronic document. When required, witness signature will be obtained similarly as described for the investigator and participant.

Manual (non-electronic) signature on electronic document:

When a manual signature on an electronic document is used for the documentation of consent at the NIH Clinical Center, this study will use the following to obtain the required signatures:

- Adobe platform (which is not 21 CFR Part 11 compliant); or,
- iMedConsent platform (which is 21 CFR Part 11 compliant)

During the consent process, participants and investigators will view individual copies of the approved consent document on screens at their respective locations (if remote consent); the same screen may be used when in the same location, but is not required.

Both the investigator and the participant will sign the document using a finger, stylus or mouse. Note: Refer to the CCR SOP PM-2, Obtaining and Documenting the Informed Consent Process for additional information (e.g., verification of participant identity when obtaining consent remotely) found at: https://ccrod.cancer.gov/confluence/pages/viewpage.action?pageId=73203825.

17.5.1 Consent Process for Adults Who Lack Capacity to Research Participation

For participants addressed in section **17.3**, an LAR will be identified consistent with Policy 403 and informed consent obtained from the LAR, as described in Section **17.5**

18 REGULATORY AND OPERATIONAL CONIDERATIONS

18.1 STUDY DISCONTINUATION AND CLOSURE

This study may be temporarily suspended or prematurely terminated if there is sufficient reasonable cause. Written notification, documenting the reason for study suspension or termination, will be provided by the suspending or terminating party to study participants, investigator, funding agency, the Investigational New Drug (IND) or Investigational Device Exemption (IDE) sponsor and regulatory authorities. If the study is prematurely terminated or suspended, the Principal Investigator (PI) will promptly inform study participants, the Institutional Review Board (IRB), and sponsor and will provide the reason(s) for the termination or suspension. Study participants will be contacted, as applicable, and be informed of changes to study visit schedule.

Circumstances that may warrant termination or suspension include, but are not limited to:

- Determination of unexpected, significant, or unacceptable risk to participants
- Demonstration of efficacy that would warrant stopping
- Insufficient compliance to protocol requirements
- Data that are not sufficiently complete and/or evaluable
- Determination that the primary endpoint has been met
- Determination of futility

Study may resume once concerns about safety, protocol compliance, and data quality are addressed, and satisfy the sponsor, IRB and as applicable, Food and Drug Administration (FDA).

18.2 QUALITY ASSURANCE AND QUALITY CONTROL

The clinical site will perform internal quality management of study conduct, data and biological specimen collection, documentation and completion. An individualized quality management plan will be developed to describe a site's quality management.

Quality control (QC) procedures will be implemented beginning with the data entry system and data QC checks that will be run on the database will be generated. Any missing data or data anomalies will be communicated to the site(s) for clarification/resolution.

Following written Standard Operating Procedures (SOPs), the monitors will verify that the clinical trial is conducted and data are generated and biological specimens are collected, documented (recorded), and reported in compliance with the protocol, International Conference on Harmonisation Good Clinical Practice (ICH GCP), and applicable regulatory requirements (e.g., Good Laboratory Practices (GLP), Good Manufacturing Practices (GMP)).

The investigational site will provide direct access to all trial related sites, source data/documents, and reports for the purpose of monitoring and auditing by the sponsor, and inspection by local and regulatory authorities.

18.3 CONFLICT OF INTEREST POLICY

The independence of this study from any actual or perceived influence, such as by the pharmaceutical industry, is critical. Therefore, any actual conflict of interest of persons who have a role in the design, conduct, analysis, publication, or any aspect of this trial will be disclosed and managed. Furthermore, persons who have a perceived conflict of interest will be required to have such conflicts managed in a way that is appropriate to their participation in the design and conduct of this trial. The study leadership in conjunction with the National Cancer Institute has established

policies and procedures for all study group members to disclose all conflicts of interest and will establish a mechanism for the management of all reported dualities of interest.

18.4 Confidentiality and Privacy

Participant confidentiality and privacy is strictly held in trust by the participating investigators, their staff, and the sponsor(s). This confidentiality is extended to cover testing of biological samples and genetic tests in addition to the clinical information relating to participants. Therefore, the study protocol, documentation, data, and all other information generated will be held in strict confidence. No information concerning the study or the data will be released to any unauthorized third party without prior written approval of the sponsor.

All research activities will be conducted in as private a setting as possible.

The study monitor, other authorized representatives of the sponsor, representatives of the Institutional Review Board (IRB), and/or regulatory agencies may inspect all documents and records required to be maintained by the investigator, including but not limited to, medical records (office, clinic, or hospital) and pharmacy records for the participants in this study. The clinical study site will permit access to such records.

The study participant's contact information will be securely stored at the/each clinical site for internal use during the study. At the end of the study, all records will continue to be kept in a secure location for as long a period as dictated by the reviewing IRB, Institutional policies, or sponsor requirements.

Study participant research data, which is for purposes of statistical analysis and scientific reporting, will be transmitted to and stored at CTEP. This will not include the participant's contact or identifying information. Rather, individual participants and their research data will be identified by a unique study identification number. The study data entry and study management systems used by the clinical site(s) and by CTEP research staff will be secured and password protected. At the end of the study, all study databases will be archived by CTEP.

To further protect the privacy of study participants, a Certificate of Confidentiality has been issued by the National Institutes of Health (NIH). This certificate protects identifiable research information from forced disclosure. It allows the investigator and others who have access to research records to refuse to disclose identifying information on research participation in any civil, criminal, administrative, legislative, or other proceeding, whether at the federal, state, or local level. By protecting researchers and institutions from being compelled to disclose information that would identify research participants, Certificates of Confidentiality help achieve the research objectives and promote participation in studies by helping assure confidentiality and privacy to participants.

19 REFERENCES

1. Enomoto T, Aoki M, Hamasaki M, Abe H, Nonaka M, Inoue T, et al. Midline Glioma in Adults: Clinicopathological, Genetic, and Epigenetic Analysis. Neurol Med Chir (Tokyo). 2020;60(3):136-46.

2. Jiang H, Yang K, Ren X, Cui Y, Li M, Lei Y, et al. Diffuse midline glioma with an H3 K27M mutation: A comparison integrating the clinical, radiological, molecular features between adult and pediatric patients. Neuro Oncol. 2019.

3. Manjunath N, Jha P, Singh J, Raheja A, Kaur K, Suri A, et al. Clinico-pathological and molecular characterization of diffuse midline gliomas: is there a prognostic significance? Neurological sciences : official journal of the Italian Neurological Society and of the Italian Society of Clinical Neurophysiology. 2020.

4. Meyronet D, Esteban-Mader M, Bonnet C, Joly MO, Uro-Coste E, Amiel-Benouaich A, et al. Characteristics of H3 K27M-mutant gliomas in adults. Neuro Oncol. 2017;19(8):1127-34.

5. Alli S, Figueiredo CA, Golbourn B, Sabha N, Wu MY, Bondoc A, et al. Brainstem blood brain barrier disruption using focused ultrasound: A demonstration of feasibility and enhanced doxorubicin delivery. J Control Release. 2018;281:29-41.

6. Saunders NR, Habgood MD, Møllgård K, Dziegielewska KM. The biological significance of brain barrier mechanisms: help or hindrance in drug delivery to the central nervous system? F1000Res. 2016;5:F1000 Faculty Rev-313.

7. Subashi E, Cordero FJ, Halvorson KG, Qi Y, Nouls JC, Becher OJ, et al. Tumor location, but not H3.3K27M, significantly influences the blood-brain-barrier permeability in a genetic mouse model of pediatric high-grade glioma. Journal of neuro-oncology. 2016;126(2):243-51.

8. William GBS, Alison SB, Max W, David J, Owen L, Marcella JW, et al. The distribution, clearance, and brainstem toxicity of panobinostat administered by convection-enhanced delivery. Journal of Neurosurgery: Pediatrics PED. 2018;22(3):288-96.

9. Bailey CP, Figueroa M, Mohiuddin S, Zaky W, Chandra J. Cutting Edge Therapeutic Insights Derived from Molecular Biology of Pediatric High-Grade Glioma and Diffuse Intrinsic Pontine Glioma (DIPG). Bioengineering (Basel). 2018;5(4):88.

10. Gallitto M, Lazarev S, Wasserman I, Stafford JM, Wolden SL, Terezakis SA, et al. Role of Radiation Therapy in the Management of Diffuse Intrinsic Pontine Glioma: A Systematic Review. Adv Radiat Oncol. 2019;4(3):520-31.

11. Blakeley JO, Olson J, Grossman SA, He X, Weingart J, Supko JG, et al. Effect of blood brain barrier permeability in recurrent high grade gliomas on the intratumoral pharmacokinetics of methotrexate: a microdialysis study. Journal of neuro-oncology. 2009;91(1):51-8.

12. Elmquist WF, Sawchuk RJ. Application of Microdialysis in Pharmacokinetic Studies. Pharmaceutical Research. 1997;14(3):267-88.

13. Jackson S, Weingart J, Nduom EK, Harfi TT, George RT, McAreavey D, et al. The effect of an adenosine A(2A) agonist on intra-tumoral concentrations of temozolomide in patients with recurrent glioblastoma. Fluids Barriers CNS. 2018;15(1):2-.

14. Ketharanathan N, Yamamoto Y, Rohlwink UK, Wildschut ED, Mathôt RAA, de Lange ECM, et al. Combining Brain Microdialysis and Translational Pharmacokinetic Modeling to Predict Drug Concentrations in Pediatric Severe Traumatic Brain Injury: The Next Step Toward Evidence-Based Pharmacotherapy? Journal of Neurotrauma. 2018;36(1):111-7.

15. Kett-White R, Hutchinson PJ, Czosnyka M, Boniface S, Pickard JD, Kirkpatrick PJ. Multi-Modal Monitoring of Acute Brain Injury. In: Pickard JD, Dolenc VV, Antunes JL, Reulen HJ, Sindou M, Strong AJ, et al., editors. Advances and Technical Standards in Neurosurgery. Vienna: Springer Vienna; 2002. p. 87-134.

16. Portnow J, Badie B, Chen M, Liu A, Blanchard S, Synold TW. The neuropharmacokinetics of temozolomide in patients with resectable brain tumors: potential implications for the current approach to chemoradiation. Clin Cancer Res. 2009;15(22):7092-8.

17. Shannon RJ, Carpenter KLH, Guilfoyle MR, Helmy A, Hutchinson PJ. Cerebral microdialysis in clinical studies of drugs: pharmacokinetic applications. J Pharmacokinet Pharmacodyn. 2013;40(3):343-58.

18. Thelin EP, Carpenter KLH, Hutchinson PJ, Helmy A. Microdialysis Monitoring in Clinical Traumatic Brain Injury and Its Role in Neuroprotective Drug Development. The AAPS Journal. 2017;19(2):367-76.

19. Ungerstedt U. Microdialysis—principles and applications for studies in animals and man. Journal of Internal Medicine. 1991;230(4):365-73.

20. Jackson S, Weingart J, Nduom EK, Harfi TT, George RT, McAreavey D, et al. The effect of an adenosine A2A agonist on intra-tumoral concentrations of temozolomide in patients with recurrent glioblastoma. Fluids and barriers of the CNS. 2018;15(1):2.

21. Lynes J, Jackson S, Sanchez V, Dominah G, Wang X, Kuek A, et al. Cytokine Microdialysis for Real-Time Immune Monitoring in Glioblastoma Patients Undergoing Checkpoint Blockade. Neurosurgery. 2019;84(4):945-53.

22. Enblad P, Valtysson J, Andersson J, Lilja A, Valind S, Antoni G, et al. Simultaneous intracerebral microdialysis and positron emission tomography in the detection of ischemia in patients with subarachnoid hemorrhage. J Cereb Blood Flow Metab. 1996;16(4):637-44.

23. Hillered L, Persson L. Neurochemical monitoring of the acutely injured human brain. Scandinavian journal of clinical and laboratory investigation Supplementum. 1999;229:9-18.

24. Ketharanathan N, Yamamoto Y, Rohlwink UK, Wildschut ED, Mathot RAA, de Lange ECM, et al. Combining Brain Microdialysis and Translational Pharmacokinetic Modeling to Predict Drug Concentrations in Pediatric Severe Traumatic Brain Injury: The Next Step Toward Evidence-Based Pharmacotherapy? Journal of neurotrauma. 2019;36(1):111-7.

25. Kett-White R, Hutchinson PJ, Czosnyka M, Boniface S, Pickard JD, Kirkpatrick PJ. Multi-modal monitoring of acute brain injury. Advances and technical standards in neurosurgery. 2002;27:87-134.

26. Patet C, Suys T, Carteron L, Oddo M. Cerebral Lactate Metabolism After Traumatic Brain Injury. Current neurology and neuroscience reports. 2016;16(4):31.

27. Persson L, Valtysson J, Enblad P, Warme PE, Cesarini K, Lewen A, et al. Neurochemical monitoring using intracerebral microdialysis in patients with subarachnoid hemorrhage. Journal of neurosurgery. 1996;84(4):606-16.

28. Thelin EP, Carpenter KL, Hutchinson PJ, Helmy A. Microdialysis Monitoring in Clinical Traumatic Brain Injury and Its Role in Neuroprotective Drug Development. The AAPS journal. 2017;19(2):367-76.

29. Ungerstedt U. Microdialysis--principles and applications for studies in animals and man. Journal of internal medicine. 1991;230(4):365-73.

30. Anderson RC, Kennedy B, Yanes CL, Garvin J, Needle M, Canoll P, et al. Convection-enhanced delivery of topotecan into diffuse intrinsic brainstem tumors in children. Journal of neurosurgery Pediatrics. 2013;11(3):289-95.

31. Heiss JD, Jamshidi A, Shah S, Martin S, Wolters PL, Argersinger DP, et al. Phase I trial of convection-enhanced delivery of IL13-Pseudomonas toxin in children with diffuse intrinsic pontine glioma. Journal of neurosurgery Pediatrics. 2018;23(3):333-42.

32. Souweidane MM, Kramer K, Pandit-Taskar N, Zhou Z, Haque S, Zanzonico P, et al. Convection-enhanced delivery for diffuse intrinsic pontine glioma: a single-centre, dose-escalation, phase 1 trial. Lancet Oncol. 2018;19(8):1040-50.

33. Zhou Z, Singh R, Souweidane MM. Convection-Enhanced Delivery for Diffuse Intrinsic Pontine Glioma Treatment. Current neuropharmacology. 2017;15(1):116-28.

34. Warren KE. Beyond the Blood:Brain Barrier: The Importance of Central Nervous System (CNS) Pharmacokinetics for the Treatment of CNS Tumors, Including Diffuse Intrinsic Pontine Glioma. Frontiers in oncology. 2018;8:239.

35. Liu L, Zhang X, Lou Y, Rao Y, Zhang X. Cerebral microdialysis in glioma studies, from theory to application. J Pharm Biomed Anal. 2014;96:77-89.

36. Raiteri M. Functional Pharmacology in Human Brain. Pharmacological Reviews. 2006;58(2):162-93.

37. Sun Y, Sun Y, Yan K, Li Z, Xu C, Geng Y, et al. Potent anti-tumor efficacy of palbociclib in treatment-naïve H3.3K27M-mutant diffuse intrinsic pontine glioma. EBioMedicine. 2019;43:171-9.

38. O'Leary B, Finn RS, Turner NC. Treating cancer with selective CDK4/6 inhibitors. Nat Rev Clin Oncol. 2016;13(7):417-30.

39. Lee KA, Shepherd STC, Johnston SRD. Abemaciclib, a potent cyclin-dependent kinase 4 and 6 inhibitor, for treatment of ER-positive metastatic breast cancer. Future Oncology. 2019.

40. Barroso-Sousa R, Shapiro GI, Tolaney SM. Clinical Development of the CDK4/6 Inhibitors Ribociclib and Abemaciclib in Breast Cancer. Breast care (Basel, Switzerland). 2016;11(3):167-73.

41. Patnaik A, Rosen LS, Tolaney SM, Tolcher AW, Goldman JW, Gandhi L, et al. Efficacy and Safety of Abemaciclib, an Inhibitor of CDK4 and CDK6, for Patients with Breast Cancer, Non-Small Cell Lung Cancer, and Other Solid Tumors. Cancer Discov. 2016;6(7):740-53.

42. Barroso-Sousa R, Shapiro GI, Tolaney SM. Clinical Development of the CDK4/6 Inhibitors Ribociclib and Abemaciclib in Breast Cancer. Breast care (Basel, Switzerland). 2016;11(3):167-73.

43. Raub TJ, Wishart GN, Kulanthaivel P, Staton BA, Ajamie RT, Sawada GA, et al. Brain Exposure of Two Selective Dual CDK4 and CDK6 Inhibitors and the Antitumor Activity of CDK4 and CDK6 Inhibition in Combination with Temozolomide in an Intracranial Glioblastoma Xenograft. Drug Metabolism and Disposition. 2015;43(9):1360.

44. Ortega S, Malumbres M, Barbacid M. Cyclin D-dependent kinases, INK4 inhibitors and cancer. Biochimica et biophysica acta. 2002;1602(1):73-87.

45. Sherr CJ. Cancer cell cycles. Science (New York, NY). 1996;274(5293):1672-7.

46. Christina H, Philipp K, Matthias F, Thorsten S, Maximilian IR. Update on the diagnostic value and safety of stereotactic biopsy for pediatric brainstem tumors: a systematic review and meta-analysis of 735 cases. Journal of Neurosurgery: Pediatrics PED. 2017;20(3):261-8.

47. Gupta N, Goumnerova LC, Manley P, Chi SN, Neuberg D, Puligandla M, et al. Prospective feasibility and safety assessment of surgical biopsy for patients with newly diagnosed diffuse intrinsic pontine glioma. Neuro-Oncology. 2018;20(11):1547-55.

48. Kline CN, Joseph NM, Grenert JP, van Ziffle J, Talevich E, Onodera C, et al. Targeted next-generation sequencing of pediatric neuro-oncology patients improves diagnosis, identifies pathogenic germline mutations, and directs targeted therapy. Neuro-oncology. 2017;19(5):699-709.

49. Mueller S, Jain P, Liang WS, Kilburn L, Kline C, Gupta N, et al. A pilot precision medicine trial for children with diffuse intrinsic pontine glioma—PNOC003: A report from the Pacific Pediatric Neuro-Oncology Consortium. International Journal of Cancer. 2019;145(7):1889-901.

50. Mueller S, Jain P, Liang WS, Kilburn L, Kline C, Gupta N, et al. A pilot precision medicine trial for children with diffuse intrinsic pontine glioma-PNOC003: A report from the Pacific Pediatric Neuro-Oncology Consortium. International journal of cancer. 2019.

51. Magnoni S, Esparza TJ, Conte V, Carbonara M, Carrabba G, Holtzman DM, et al. Tau elevations in the brain extracellular space correlate with reduced amyloid-β levels and predict adverse clinical outcomes after severe traumatic brain injury. Brain. 2012;135(Pt 4):1268-80.

52. Brody DL, Magnoni S, Schwetye KE, Spinner ML, Esparza TJ, Stocchetti N, et al. Amyloid-beta dynamics correlate with neurological status in the injured human brain. Science (New York, NY). 2008;321(5893):1221-4.

53. Cage TA, Samagh SP, Mueller S, Nicolaides T, Haas-Kogan D, Prados M, et al. Feasibility, safety, and indications for surgical biopsy of intrinsic brainstem tumors in children. Childs Nerv Syst. 2013;29(8):1313-9.

54. Puget S, Beccaria K, Blauwblomme T, Roujeau T, James S, Grill J, et al. Biopsy in a series of 130 pediatric diffuse intrinsic Pontine gliomas. Childs Nerv Syst. 2015;31(10):1773-80.

55. Portnow J, Badie B, Liu X, Frankel P, Mi S, Chen M, et al. A pilot microdialysis study in brain tumor patients to assess changes in intracerebral cytokine levels after craniotomy and in response to treatment with a targeted anti-cancer agent. Journal of neuro-oncology. 2014;118(1):169-77.

56. Blakeley JO, Olson J, Grossman SA, He X, Weingart J, Supko JG, et al. Effect of blood brain barrier permeability in recurrent high grade gliomas on the intratumoral pharmacokinetics of methotrexate: a microdialysis study. Journal of neuro-oncology. 2009;91(1):51-8.

57. Tabatabaei P, Visse E, Bergstrom P, Brannstrom T, Siesjo P, Bergenheim AT. Radiotherapy induces an immediate inflammatory reaction in malignant glioma: a clinical microdialysis study. Journal of neuro-oncology. 2017;131(1):83-92.

58. Marcus HJ, Carpenter KL, Price SJ, Hutchinson PJ. In vivo assessment of high-grade glioma biochemistry using microdialysis: a study of energy-related molecules, growth factors and cytokines. J Neurooncol. 2010;97(1):11-23.

59. Field M, Witham TF, Flickinger JC, Kondziolka D, Lunsford LD. Comprehensive assessment of hemorrhage risks and outcomes after stereotactic brain biopsy. Journal of neurosurgery. 2001;94(4):545-51.

60. Shooman D, Belli A, Grundy PL. Image-guided frameless stereotactic biopsy without intraoperative neuropathological examination. Journal of neurosurgery. 2010;113(2):170-8.

61. Thelin EP, Nelson DW, Ghatan PH, Bellander BM. Microdialysis Monitoring of CSF Parameters in Severe Traumatic Brain Injury Patients: A Novel Approach. Front Neurol. 2014;5:159.

62. Portnow J, Synold TW, Badie B, Tirughana R, Lacey SF, D'Apuzzo M, et al. Neural Stem Cell-Based Anticancer Gene Therapy: A First-in-Human Study in Recurrent High-Grade Glioma Patients. Clinical cancer research : an official journal of the American Association for Cancer Research. 2017;23(12):2951-60.

20 APPENDICES

20.1 APPENDIX A: PERFORMANCE STATUS CRITERIA

**ECOG Performance Status Scale**		**Karnofsky Performance Scale**	
Grade	Descriptions	Percent	Description
0	Normal activity. Fully active, able to carry on all pre-disease performance without restriction.	100	Normal, no complaints, no evidence of disease.
		90	Able to carry on normal activity; minor signs or symptoms of disease.
1	Symptoms, but ambulatory. Restricted in physically strenuous activity, but ambulatory and able to carry out work of a light or sedentary nature (e.g., light housework, office work).	80	Normal activity with effort; some signs or symptoms of disease.
		70	Cares for self, unable to carry on normal activity or to do active work.
2	In bed <50% of the time. Ambulatory and capable of all self-care, but unable to carry out any work activities. Up and about more than 50% of waking hours.	60	Requires occasional assistance but is able to care for most of his/her needs.
		50	Requires considerable assistance and frequent medical care.
3	In bed >50% of the time. Capable of only limited self-care, confined to bed or chair more than 50% of waking hours.	40	Disabled, requires special care and assistance.
		30	Severely disabled, hospitalization indicated. Death not imminent.
4	100% bedridden. Completely disabled. Cannot carry on any self-care. Totally confined to bed or chair.	20	Very sick, hospitalization indicated. Death not imminent.
		10	Moribund, fatal processes progressing rapidly.
5	Dead.	0	Dead.

20.2 APPENDIX B: PARTICIPANT CLINICAL TRIAL WALLET CARD

NIH NATIONAL CANCER INSTITUTE

CLINICAL TRIAL WALLET CARD

Show this card to all of your healthcare providers and keep it with you in case you go to the emergency room.

Patient Name:

Diagnosis:

Study Doctor:

Study Doctor Phone #:

NCI Trial #: 10444

Study Drug(S):
Abemaciclib
Temozolomide

For more information: 1-800-4-CANCER
cancer.gov | clinicaltrials.gov

20.3 APPENDIX C: PK COLLECTION FORM

Plasma PK:

Pre and post-surgery only- Abemaciclib only (**Table**): Collect samples per designated time points (pre-dose, time of last dose pre-surgery and then 1, 2, 4, 6, 8, 10, 24, 48 and 72 hours post catheter insertion). Collect 3mL of blood into 4ml (lavender top) K2EDTA collection tubes. Mix by gently inverting 6 times. Place the tubes immediately on wet ice-water bath after collection. Centrifuge samples within 2 hours after collection at 1300 x g for 10 minutes at 4°C. Separate the plasma from the blood into a labeled polypropylene freezer vial. The samples should be processed to plasma within 30 minutes from centrifugation. Plasma should then be stored frozen at -80°C until subsequent batch analysis.

Maintenance therapy- Abemaciclib and Temozolomide: Collect samples per designated time points on day 5 of every other cycle. Collect 3mL of blood into 4mL(lavender top) K2EDTA collection tubes for abemaciclib concentration. Collect 3mL of blood in 4mL (green top) Sodium heparin collection tubes for temozolomide concentration. Mix by gently inverting 6 times. Place the tubes immediately on wet ice-water bath after collection. Centrifuge samples within 2 hours after collection at 1300 x g for 10 minutes at 4°C. Place the centrifuged tubes on wet ice. Separate the plasma from the blood into a labeled polypropylene freezer vial. The samples should be processed to plasma within 30 minutes from centrifugation. Plasma should then be stored frozen at -80°C until subsequent batch analysis.

Dialysate PK:

Post-surgery only- Collect samples per designated time points (2, 6, 10, 14, 18, 22, 26, 30, 34, 38, 40, 44, and 48 hours post catheter insertion). Samples should be collected, labeled and placed in the -80°C freezer within 1 hour from collection.

Table 7:Pre-surgery and Post-surgery Abemaciclib Plasma and Dialysate Pharmacokinetic Collection Form

**Abemaciclib Concentration Data**	
Dose: _________mg	Last ingestion time: ___:____

Protocol sample time	Sample due date	Abemaciclib plasma PK sample time	Abemaciclib dialysate PK collection time	Tech/Nurse initials	Comments
Pre-ingestion					
Time of last dose pre-surgery					
1 hr post insertion					
2 hrs post insertion					

Protocol sample time	Sample due date	Abemaciclib plasma PK sample time	Abemaciclib dialysate PK collection time	Tech/Nurse initials	Comments
4 hrs post insertion					
6 hrs post insertion					
8 hrs post insertion					
10 hrs post insertion					
14 hrs post insertion					
18 hrs post insertion					
22 hrs post insertion					
24 hrs post insertion					
26 hrs post insertion					
30 hrs post insertion					
34 hrs post insertion					
38 hrs post insertion					
40 hrs post insertion					
44 hrs post insertion					
48 hrs post insertion					
72 hrs post insertion					

PLEASE CALL Clinical Pharmacology x102-11964 PRIOR TO EACH PLASMA COLLECTION

Print Name	Signature

If you have drawn a blood sample, please print and sign your name.

20.4 APPENDIX D: ASHION ANALYTICS SAMPLE GENOMICS REPORT

GEM ExTra™ Report

ASHION
A TGen® Clinical Laboratory

Report Date:

Patient:	Ordering Client:
Gender:	Specimen Type:
DOB:	Specimen Site:
Medical Record #:	Tumor Collection Date:
Client Accession #:	Normal Collection Date:
Ordering Physician:	Received Date:

Genomic Snapshot
- Analytes sequenced: DNA+RNA
- Actionable Targets: 4
- TMB: Low
- MSI: Stable
- Clinical Trials: Yes

Diagnosis: **Diffuse Midline Glioma**

TUMOR GENOMIC ALTERATIONS[1]				
H3F3A		PDGFB		TP53
GENOMIC TARGETS	FDA-APPROVED DRUGS -for patient's cancer	FDA-APPROVED DRUGS -for another cancer	DRUGS PREDICTED NON-BENEFICIAL	POTENTIAL CLINICAL TRIALS
4	0	5	0	Yes
H3F3A (K28M)		panobinostat, vorinostat		Yes
PDGFB (Amplification)		dasatinib, imatinib, lenvatinib		Yes
TP53 (C277F)				Yes

TUMOR MUTATION BURDEN (TMB)		
LOW (1 mut/Mb)		No

MICROSATELLITE STATUS (MSI)		
STABLE		No

ADDITIONAL SIGNIFICANT ALTERATIONS		
MYCN (Amplification)		No

***NOTE:** Certain drugs associated with variants detected in this case may not cross the blood-brain barrier; treating physician discretion is necessary.

****NOTE:** the tumor sample harbors a **focal gain of 4q12 region** that has previously been implicated in glioma (Holtkamp N et al., 2007; PMID: 17504929). The gene **PDGFRA** is located in this region and was **found to be amplified** slightly below our threshold of reporting. PDGFRA encodes the tyrosine kinase receptor human platelet-derived growth factor receptor alpha, also known as Pdgfr-alpha. PDGFR activation is also a common feature in pediatric high grade gliomas, including H3F3A K27-mutated diffuse midline gliomas, typically due to PDGFRA amplification or mutation but are spatially heterogeneous in

[1] Alterations with predictive value according to Ashion's database and/or clinical trials identified by Ashion. For a complete list of alterations, please see the VUS section near the end of the report.

ASHION
A TGen® Clinical Laboratory

nature (Hoffman LM et al., 2016; PMID: 26727948; Mackay A et al., 2017; PMID: 28966033). PDGFRA amplification, overexpression or activating mutation may predict sensitivity to small molecule tyrosine kinase inhibitors that target Pdgfrs and other kinases, as well as agents that target the PI3K-AKT-mTOR pathway such as imatinib, pazopanib, sorafenib, sunitinib, nilotinib, temsirolimus, regorafenib, ponatinib, dasatinib, nindetanib and everolimus. Pre-clinical data suggests that use of PDGFR inhibitors as a single agent may not be sufficient to cause regression of pediatric HGG, but could be a useful in combination (Paugh BS et al., 2013; PMID: 23970477). By Kaplan-Meier analysis, non-brainstem HGG with PDGFRA amplification carried a worse prognosis than non-brainstem HGG without PDGFRA amplification (P = 0.021). There were no pediatric patients with PDGFRA-amplified HGG that survived longer than two years (Koschmann C et al., 2016;PMID: 27582545). Certain clinical trials are enrolling pediatric patients with PDGFR inhibitors (NCT02389309, NCT01956669, NCT02747537, NCT02432274).

***NOTE:** This tumor sample demonstrates **loss on 17p**, a chromosomal alteration associated with shorter overall survival in pediatric high grade gliomas (Mackay A et al., 2017; PMID: 28966033).

****NOTE:** This tumor harbors **amplification on 22q13.1**, which contains several genes previously implicated in cancer, including PDGFB, CSNK1E and APOBEC3B. CSNK1E encodes casein kinase 1 epsilon, a serine/threonine protein kinase that has been linked to regulation of beta-catenin and MYC pathway activation in other tumor types (Toyoshima M et al., 2012; PMID: 22623531), and was recently shown to play a role in regulating glioblastoma proliferation and survival (Varghese R et al., 2018; PMID: 30206363). APOBEC3B encodes a DNA cytosine deaminase that is overexpressed in a variety of tumor types and has been associated with an APOBEC mutagenesis pattern (Burns M et al., 2013; PMID: 23389445, Burns M., et al., 2013; PMID: 23852168, Leonard B et al., 2013; PMID: 24154874, Boichard A et al., 2017; PMID: 28405512, Zou et al., 2017; PMID: 28572915). Evaluation of the potential association between APOBEC3 family members, APOBEC-related mutagenesis, and immunotherapy response is underway in other tumor types (Chen H et al., 2019; PMID: 30719225, Boichard A et al., 2018; PMID: 30723579, Wang S et al., 2018; PMID: 29695832). The role of APOBEC3B amplification in diffuse midline glioma remains to be determined.

20.5 APPENDIX E: STUDY CALENDAR

NOTE: Additional assessments may be done as clinically indicated See Section 6.1 for details on drug administration

**STUDIES TO BE OBTAINED**	**Screening**[1]	**Baseline**[2]	**Abemaciclib Administration**[3]	**Microdialysis Catheter in place**[4]		**Catheter Removal**[5]	**Maintenance Therapy**[6]	**Post- Treatment**	
			Day 1-5	Day 5	Day 6	Day 7 (>48 hours post-op)		Safety[7]	Long-Term Follow-Up[8]
History	X	X	X			X	X	X	X
Physical Exam, vital signs[9]	X	X	X	X	X	X	X	X	
Performance Status[10]	X	X				X		X	
Clinical Course									X[11]
Chest X-ray		X				As clinically indicated			
EKG, Echocardiogram	X	X				As clinically indicated			
CBC, differential, platelets	X	X		X[13]	X	X	X[12]	X	
PT/PTT and INR	X	X		X[13]		X			
Chemistries[14]	X	X		X[13]	X	X	X	X	
Total protein/albumin, amylase, lipase	X	X		X[13]	X	X		X	
ALT AST and bilirubin	X	X		X[13]		X		X	
Urinalysis	X	X				X		X	
Pregnancy Test (serum)	X	X[15]							
Brain MRI[16]	X[17]	X				As clinically indicated	X	As clinically indicated	
Brain CT				X[18]		As clinically indicated			
Tablet count monitoring[19]				X			X		
Biopsy/resection				X[20]					
PK studies- plasma		See Section 5 for timing and collection details on correlative studies							
PK studies- dialysate		See Section 5 for timing and collection details on correlative studies							

STUDIES TO BE OBTAINED	Screening[1]	Baseline[2]	Abemaciclib Administration[3]	Microdialysis Catheter in place[4]		Catheter Removal[5]	Maintenance Therapy[6]	Post- Treatment	
			Day 1-5	Day 5	Day 6	Day 7 (>48 hours post-op)		Safety[7]	Long-Term Follow-Up[8]
PD studies[21]		See Section 5 for timing and collection details on correlative studies							
Correlative studies on tumor and blood		See Section 5 for timing and collection details on correlative studies							

[1] Screening tests should be performed within 14 days prior to enrollment unless otherwise specified

[2] Screening tests performed within the specified time frame for baseline do not need to be repeated. Baseline evaluations to be performed within 14 days of first dose, unless otherwise noted

[3] The first dose will be taken in the CCR Day hospital and monitored for toleration up to 2 hours after ingestion. Other doses to be taken at home.

[4] Placed in OR after patient has been taking abemaciclib for 4.5 days (Day1-5)

[5] At bedside

[6] For patients that receive continued abemaciclib wih temozolomide treatment, assessments to be obtained before the start of every cycle with the exception of Brain MRI, which will be obtained after every 3 cycles, and PK studies, which will be ontained on Day 5 of every other cycle

[7] Should be conducted approximately 30 days (+/- 7 days) after the last dose of study drug (in-person or tele-health visit).

[8] A patient or their local health care provider will be contacted via phone every 6 months (+/- 14 days) until death per Section 6.4.3

[9] A complete standard physical examination including height weight and vitals will be performed at screening, baseline and at time of first dose of medication, subsequent physical exams (including height and weight) will be targeted based on signs and symptoms of presenting patient. Vital signs: heart rate, temperature, blood pressure, respiratory rate (to be obtained each visit) and O_2 saturation by pulse oximetry at screening and baseline and then as clinically indicated. Isolated missing vital signs will be noted, but not reported as a protocol deviation. Neurologic exams will be completed every 2 hours for the first 24hours while in the ICU, then q4h while awake for the remainder of the ICU monitoring period. Physical exam and labs will only be performed for in-person visits that occur approximately 30 days (+/- 7 days) after the last dose of study drug.

[10] Measured according to Karnofsky

[11] To include: Therapy received for their disease, dates of treatment and response to treatment, and tumor recurrence and/or progression, and survival status

[12] See Section 7.4 for monitoring during maintenance therapy with repeat CBC on Day 22 (21 days after first dose) or within 48 hours of that day after temozolomide administration and weekly until the ANC is above 1.5 x 10^9/L and platelet count exceeds 100 x 10^9/L.

[13] Prior to biopsy/resection (within 7 days)

[14] Chemistries: Electrolytes (including sodium, potassium, chloride, CO_2), calcium, phosphorus, magnesium, creatinine, BUN, glucose, AST, bilirubin (total and direct). See separate entry in study calendar for ALT monitoring parameters.

[15] Within 7 days of first dose of drug

[16] Gadolinium-Enhanced Brain MRI- T1 pre- and post-contrast, T2, FLAIR, performed in at least 2 planes; GRE. Additional brain MRI sequences: DCE-MRI perfusion and diffusion-weighted imaging, when possible; obtained pre-op and then every 3cycles during abemaciclib+temozolomide combination maintenance therapy until disease progression

[17] Recurrent diagnosis- no more than 14 days from recent scan showing disease recurrence/progression until first dose of study drug

[18] To be performed after the catheter is inserted.

[19] Tablet count monitoring for missed doses
[20] To be taken prior to microdialysis catheter placement in the OR, at least 6 small cores to be obtained.
[21] To be performed on biopsied tissue See Section **5** regarding details of pharmacodynamic studies.
